# Borane‐Catalyzed Water Dehydrogenation at Dioxaphospholes and ‐Phospholanes

**DOI:** 10.1002/anie.3462462

**Published:** 2026-09-26

**Authors:** Niklas Koch, Felix Wech, Tonin Leautier, Johanna Osterbrink, Urs Gellrich

**Affiliations:** ^1^ Fachgebiet Organische Chemie Universität Hohenheim Stuttgart Germany; ^2^ Organometallic Chemistry Group Okinawa Institute of Science and Technology Graduate University Okinawa Japan; ^3^ Institut Für Organische Chemie Universität Stuttgart Stuttgart Germany

**Keywords:** borane catalysis, dehydrogenation, density functional calculations, hydrogen, water activation

## Abstract

We report that water undergoes rapid oxidative addition to readily accessible dioxaphospholes and ‐phospholanes within minutes at room temperature. The resulting phenylphosphinate esters release dihydrogen under mild conditions (room temperature to 60°C) in a borane‐catalyzed process. Remarkably, a pinacol‐derived dioxaphospholane reacts spontaneously with atmospheric moisture under ambient conditions to form the corresponding phenylphosphinate ester, thereby enabling the direct utilization of water from air for catalytic hydrogen evolution. The mechanism of the borane‐catalyzed dehydrogenation was investigated by variable time normalization analysis, targeted synthesis of intermediates, and DFT calculations. These studies support a catalytic pathway initiated by hydrogen elimination from the borane adduct of the phosphinate ester, followed by retro‐hydroboration to regenerate the catalyst.

## Graphical abstract

The borane‐catalyzed dehydrogenation of phenylphosphinate esters, which are formed from the reaction of dioxaphospholes and ‐phospholanes with water or air moisture under mild conditions, is reported. The mechanism of this transformation was investigated by kinetic measurements and DFT calculations, which indicate hydrogen elimination from a phosphinate ester borane adduct and a retro‐hydroboration from the resulting phosphorane as key steps.

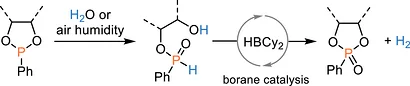

## 1 Introduction

The development of new methods for the dehydrogenation of water is relevant for the potential use of this abundant resource in energy storage applications and for transfer hydrogenations. At the molecular level, O─H bond activation is key to hydrogen release from water. Traditionally, bond activation processes are associated with oxidative addition at transition‐metal centers. However, the limited availability, high cost, and potential toxicity of precious transition metals motivate the development of alternative approaches based on more abundant main‐group elements [1]. Indeed, the first examples of O─H bond activation of alcohols via oxidative addition at a single phosphorus center date back to the 1970s [2, 3, 4]. Subsequently, Tangour et al. reported the activation of alcohols and amines by dioxaphospholes and ‐phospholanes (Scheme 1A) [5, 6]. In 2014, Radosevich and coworkers showed that geometric constraints activate phosphines toward reversible O─H and N─H oxidative additions [7, 8]. A similar strategy was used by Aldridge, Goicoechea, and coworkers for the activation of ammonia and water via E─H oxidative addition at a geometrically constrained phosphorus(III) center (Scheme 1B) [9]. However, dehydrogenation of water was not observed in these systems. The group of Dobrovetsky reported O─H and N─H splitting at a constrained phosphonium ion via a P‐center/ligand‐assisted pathway [10]. More recently, Alcarazo and coworkers described the E─H bond activation of amines and alcohols via oxidative addition at a P(III) compound with a pyrrolide pincer ligand [11]. Müller et al. reported water activation at phosphinines by phosphorus‐ligand cooperation leading to dihydrophosphinine oxides [12, 13]. Cooperative O─H bond activation through the interplay of Lewis basic and Lewis acidic centers was also recently used for the dehydrogenation of water via an elegant proton‐to‐hydride umpolung in a B─P─B Lewis pair (Scheme 1C) [14]. A similar intermolecular protocol for water dehydrogenation using an ortho‐phenylene‐bridged phosphinoborane was reported by Repo et al. [15]. We noted that oxidative addition of water to phosphorus(III) intrinsically involves a formal proton‐to‐hydride umpolung and could thus provide an entry point to water dehydrogenation on a main‐group platform. On this basis, we set out to investigate whether a frustrated Lewis pair (FLP) [16, 17, 18, 19, 20, 21, 22] can promote the dehydrogenation of phosphoranes formed upon water addition to readily accessible dioxaphospholes or ‐phospholanes. We hypothesized that an FLP comprising a sterically encumbered Lewis base and a Lewis acidic borane would enable this transformation via concerted proton and hydride abstraction (Scheme 1D).

**SCHEME 1:**
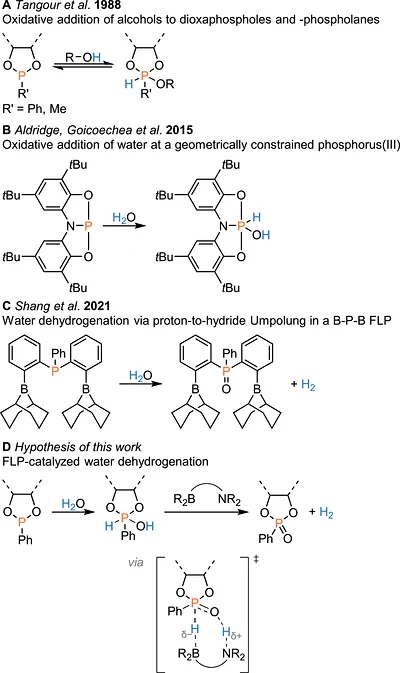
The context of this study: (A) Oxidative Addition of alcohols to dioxaphospholes and ‐phospholanes. (B) Oxidative addition of water at a geometrically constrained phosphorus(III) center. (C) Stoichiometric water dehydrogenation via proton‐to‐hydride Umpolung in a B─P─B FLP. (D) The hypothesis of this work: Oxidative addition of water to a phosphorus center and subsequent FLP‐catalyzed proton and hydride abstraction forming dihydrogen.

## 2 Results and Discussion

We commenced our study with the reactions of dioxaphosphole **1** and dioxaphospholane **2**, which were already proven to undergo oxidative addition of alcohols [5, 6], with one equivalent of water in acetonitrile. Within 15 min at room temperature, the phenylphosphinate esters **1a** and **2a** formed (Scheme 2) and were isolated in almost quantitative yield.

**SCHEME 2:**
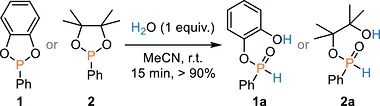
Formation of phenylphosphinate esters **1a** and **2a** from dioxaphosphole **1** and ‐phospholane **2** with water.

The presence of a P─H bond is evident from a P─H coupling with a large coupling constant (^1^ *J*_P─H_ = 585.3 and 559.5 Hz, respectively) in the ^1^H and ^31^P NMR spectra. Additionally, P─H‐bond vibrational frequencies at 2388 and 2358 cm^−1^, respectively, observed in the IR spectra of both compounds (Figures SI1 and SI3, respectively), and the molecular structures of **1a** and **2a** derived from single‐crystal x‐ray diffraction (SCXRD) support this assignment (Figure 1) [23]. In the solid‐state structures of **1a** and **2a**, intermolecular hydrogen bonding with O^…^O distances of 2.625 and 2.733 Å, respectively, is evident. A similar structural motif was reported for the product of the activation of water by a T‐shaped phosphine with an NNN‐pincer ligand [24].

**FIGURE 1:**
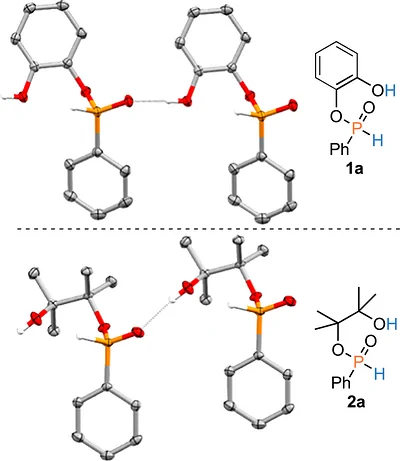
Molecular structures of the phenylphosphinate esters **1a** (top) and **2a** (bottom) derived from SCXRD measurements. Intermolecular hydrogen bonding is highlighted (gray: carbon, white: hydrogen, red: oxygen, orange: phosphorus. Ellipsoids are drawn at 50% probability. All C─H hydrogens were omitted for clarity).

When dioxaphospholane **2** was reacted with methanol instead of water under identical reaction conditions, the formation of the corresponding σ^5^‐phosphorane was observed (see Chapter S4.1), as described by Barrans et al. [25]. This indicates that formation of the phenylphosphinate esters occurs via an oxidative addition followed by a rapid isomerization. Density functional theory (DFT) calculations suggest that an oxidative addition of water to dioxaphosphole **1** and dioxaphospholane **2** to give the pentavalent phosphorus compounds 1a^*^ and 2a^*^, which structurally resemble the σ^5^‐phosphoranes formed by the addition of alcohols to **1** or **2**, is slightly endergonic (Scheme 3). However, isomerization to the phenylphosphinate esters **1a** and **2a** is exergonic, thus providing the driving force for the overall reaction. Oxidative addition to a cationic phosphorus(III) center and a subsequent isomerization to a phosphorus oxide upon ligand rearrangement was recently also reported by Dobrovetsky et al. [26].

**SCHEME 3:**
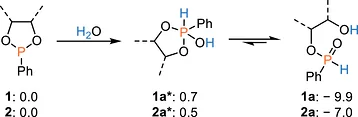
Calculated changes in Gibbs energy resulting from the addition of water to dioxaphosphole **1** and dioxaphospholane **2**. All calculations were performed within the framework of the density functional theory at the SMD(MeCN)‐*ω*B97M‐V/def2‐QZVPP//CPCM(MeCN)‐*r* ^2^SCAN‐3c level [27, 28, 29, 30, 31].

Notably, **1a** and **2a** also form upon exposure of the parent dioxaphosphole **1** and dioxaphospholane **2** to air. When samples of **1** and **2** were placed in a ventilated fumehood with approximately 35% humidity, complete formation of **1a** and **2a** was observed within 0.5 to 2 h, along with minor amounts of the corresponding phosphine oxides **1b** and **2b** (Scheme 4, see Chapter S3 for further details). While for **1**, longer exposure led to further decomposition into several new species, **2a** formed under atmospheric conditions is stable toward further decomposition.

**SCHEME 4:**
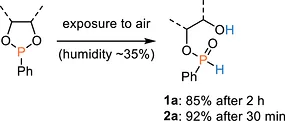
Activation of water from air by **1** and **2**.

As outlined in the introduction, the oxidative addition of water to phosphorus leads to a polarity reversal of one protic O─H hydrogen atom. We therefore hypothesized that the Lewis‐acidic side of an FLP could be used to abstract the slightly hydridic P─H hydrogen atom, while the Lewis‐basic side of the FLP could cause simultaneous deprotonation of the O─H group of the phenylphosphinate esters **1a** or **2a**. To test this hypothesis, **1a** was reacted with stoichiometric amounts of the pyridonate borane **3**, which resembles an intramolecular FLP capable of reversible hydrogen activation [32]. However, after 6 h at 80°C, conversion of **1a** back to the parent dioxaphosphole **1** was observed (Scheme 5, top). This indicates that the addition of water to **1** can be reversed. Unfortunately, this reaction also led to decomposition of the FLP **3**, as indicated by the formation of pentafluorobenzene (see Chapter S5.1). We therefore decided to replace the electron‐withdrawing pentafluorophenyl moieties at the boron center with cyclohexyl substituents. Upon reaction of **1a** with the new FLP **4**, a slow but clean formation of 2‐phenyl‐1,3,2‐benzodioxaphosphole 2‐oxide **1b** was observed within 6 h at room temperature (Scheme 5, bottom). Additionally, the formation of dihydrogen was evident from the ^1^H NMR spectrum. During this reaction, the pyridonate dicyclohexylborane **4** remained unchanged, which led us to the conclusion that **4** acts as a catalyst.

**SCHEME 5:**
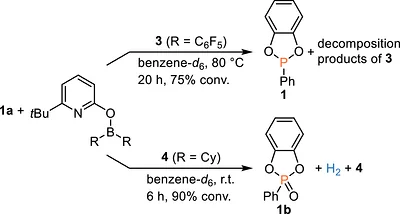
Attempts for the dehydrogenation of **1a**: With the pyridonate borane **3**, conversion back to dioxaphosphole **1** and decomposition of the FLP were observed (top). With **4**, liberation of dihydrogen and formation of phosphine oxide **1b** was achieved (bottom).

We therefore aimed to develop a protocol for a catalytic dehydrogenation of **1a** and indeed, with 10 mol% each of 6‐*tert*‐butylpyridone **5** and dicyclohexylborane **6** – both compounds of which the pyridonate borane **4** is composed of – a smooth dehydrogenation at 60°C in THF occurred within 6 h (Table 1). To our surprise, the reaction proceeds similarly in the absence of **5**, with borane **6** as the sole catalyst. **2a** can be dehydrogenated under similar conditions. Moreover, in the case of the dehydrogenation of **2a** with dicyclohexylborane **6** as catalyst, the reaction temperature could be lowered to room temperature. Without a catalyst, heating of a THF solution of **2a** led only to the formation of traces of **2b**.

**TABLE 1:** Summary of the FLP‐ or borane‐catalyzed dehydrogenation of **1a** and **2a**.

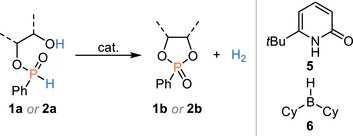			
Phosphinate ester	Catalyst	*T* / °C	Conversion
**1a**	**5 + 6**	60	quant.
**2a**	**5 + 6**	60	quant.
**1a**	**6**	60	quant.
**2a**	**6**	60	quant.
**2a**	**none**	60	traces
**1a**	**6**	25	traces
**2a**	**6**	25	quant.

Reaction conditions: 5 mL THF (0.1 M), 10 mol% catalyst, 6 h, conversion determined by ^31^P{^1^H} NMR.

We then attempted to transfer these results into a process on a scale of 1 mmol. Under optimized reaction conditions, both **1a** and **2a** could be converted to 2‐phenyl‐1,3,2‐benzodioxaphosphole 2‐oxide **1b** and 4,4,5,5‐tetramethyl‐2‐phenyl‐1,3,2‐dioxaphospholane 2‐oxide **2b**, respectively (Scheme 6). The formation of dihydrogen within these transformations was confirmed by GC analysis (see Chapter S5.5).

**SCHEME 6:**
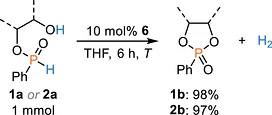
Dehydrogenation of **1a** and **2a** under optimized conditions on a preparative scale. *T*: 60°C for **1a**, rt for **2a**.

Since phenylphosphinate esters are also formed when **1** and **2** are exposed to air, we aimed to explore the possibility of using atmospheric moisture as a source of molecular hydrogen. Pleasingly, when dioxaphospholane **2** was exposed to air for 1 h and subsequently dissolved in THF and treated with 10 mol% of dicyclohexylborane **6**, complete conversion to oxide **2b** was observed (Scheme 7). Additionally, the formation of dihydrogen was confirmed by GC.

**SCHEME 7:**
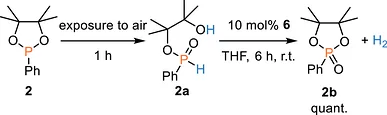
Activation of moisture from air and subsequent dehydrogenation in a one‐pot two‐step protocol.

With both the activation of water to form **1a** and **2a** and their subsequent dehydrogenation explored, we aimed to develop a procedure combining these two steps into a single one‐pot operation. Therefore, water was added to a solution of **1** or **2** in THF containing 10 mol% dicyclohexylborane **6**, and indeed, the formation of **1b** and **2b**, along with the evolution of dihydrogen, became evident (Scheme 8).

**SCHEME 8:**
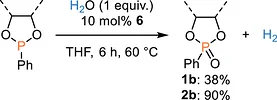
In situ water activation and dehydrogenation. Yields of phosphine oxides **1b** and **2b** are based on ^31^P{^1^H} NMR.

However, additional experiments revealed that also for **2**, this protocol required a reaction temperature of 60°C, whereas both individual steps already take place at room temperature. This finding is tentatively attributed to the deactivation of the borane catalyst by unbound water, which is only overcome at elevated temperatures. To verify this hypothesis, we tested whether small amounts of water altered the catalytic activity. Therefore, we conducted a series of experiments in which we added 5 or 10 mol% of water to the dehydrogenation reaction of **2a** catalyzed by 10 mol% dicyclohexylborane **6**. These reactions were stopped after 2 h at lower conversions to observe changes in the initial catalytic activity. Indeed, a significant decrease in the conversion to **2b** was observed (Table 2). This effect is more pronounced at room temperature than at 60°C.

**TABLE 2:** Inhibition of the reaction by small amounts of water.

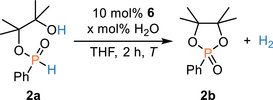			
Entries	mol% H_2_O	*T* / °C	Conversion / %
1	0	25	74
2	0	60	99
3	5	25	24
4	5	60	44
5	10	25	16
6	10	60	39

Reaction conditions: 1 mL THF (0.1 M), conversion determined by ^31^P{^1^H} NMR.

We aimed to explore the origin of the catalyst deactivation caused by water. Therefore, we reacted dicyclohexylborane **6** with water, which led to the formation of 1,1,3,3‐tetracyclohexylboroxane **7**, which we were able to isolate by crystallization (Scheme 9A). At 60°C, **7** itself catalyzes the dehydrogenation of phenylphosphinate esters **1a** and **2a** (Scheme 9B).

**SCHEME 9:**
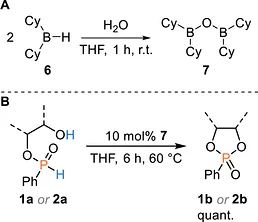
(A) Formation of 1,1,3,3‐tetracyclohexylboroxane **7** from dicyclohexylborane **6** and water. (B) Dehydrogenation of phenylphosphinate esters **1a** and **2a** catalyzed by **7**.

The finding that residual moisture slows down the catalytic reaction encouraged us to explore the intrinsic catalytic activity of our system under water‐free conditions. Therefore, a THF solution of **2a** was stored over molecular sieves for 24 h to remove traces of water. Indeed, with this carefully dried substrate, we observed 90% conversion to the corresponding oxide **2b** within 7 h at room temperature with a reduced catalyst load of only 3 mol% (Figure 2, left). With these results in hand, we aimed for a more detailed study of the catalytic activity. We carried out a Variable Time Normalization Analysis (VTNA) [33, 34, 35] to determine the reaction order with respect to the catalyst. Conversion curves were obtained for three different catalyst loadings. Analysis of the normalized data revealed a good overlap for a first‐order dependence on the catalyst concentration (Figure 2, right, see also Chapter S8.3).

**FIGURE 2:**
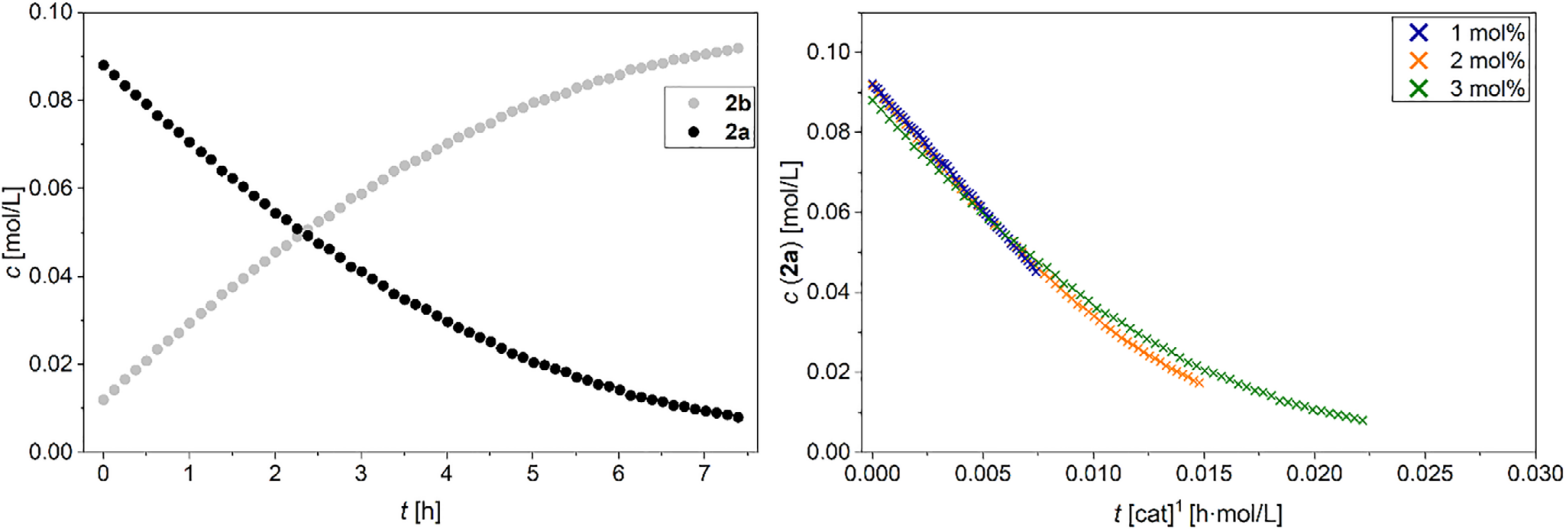
Left: Conversion of phenylphosphinate ester **2a** to phosphine oxide **2b** with 3 mol% 6 as catalyst monitored by NMR spectroscopy. Right: VTNA of the dehydrogenation of **2a** with three different catalyst loadings shows a first‐order dependence on the catalyst concentration. Measurement series with 60 data points were recorded for 1, 2, and 3 mol% catalyst loading.

To gain mechanistic insight into the experimentally observed higher reactivity of **2a** compared to **1a**, DFT calculations were performed to elucidate the key elementary steps of the catalytic cycle. According to the calculations, the initial coordination of HBCy_2_ to **1a** is slightly endergonic (Figure 3, top). Subsequent hydrogen elimination, involving the hydridic B─H bond and the protic O─H group of the phenylphosphinate ester, proceeds with an overall activation barrier of 17 kcal·mol^−^^1^. This step is exergonic. We further considered the possibility that the tetracoordinate boron intermediate **1e** is formed via the transition state **TS_1d/1e_**. The calculations indicate that the formation of this species is exergonic but reversible. The reaction then proceeds via **1d** through a step that can be interpreted as a retro‐hydroboration of a phosphine oxide (**TS_1d/1f_**). This step leads to the formation of the phosphine oxide–hydroborane adduct **1f**. This adduct subsequently dissociates into phosphine oxide **1b** and borane **6**, thereby regenerating the active catalyst and closing the catalytic cycle. The rate‐determining step is the transformation of the tetracoordinate intermediate **1e** through the transition state **TS_1d/1f_**. For the pinacol‐substituted phenylphosphinate ester **2a**, the initial coordination of HBCy_2_ to **2a** is slightly exergonic, indicating a thermodynamically favorable pre‐association (Figure 3, bottom). The corresponding hydrogen liberation exhibits an activation barrier of 18.1 kcal·mol^−^^1^. However, the formation of the tetracoordinate intermediate **2e** is significantly less exergonic. At the same time, the productive retro‐hydroboration step via **TS_2d/2f_** imparts a lower activation barrier. Consequently, the rate‐determining step shifts to the initial hydrogen release over the transition state **TS_2c/2d_**. This barrier is substantially lower than that calculated for the borane‐catalyzed dehydrogenation of the catechol‐substituted phenylphosphinate ester **1a**. Overall, the calculated energy profiles of the mechanisms depicted in Figure 3 provide a compelling explanation for the experimentally observed difference in reactivity between **1a** and **2a**. Moreover, the experimental observation that the reaction displays a first‐order dependence on the catalyst concentration is consistent with the computational prediction that hydrogen elimination from **2c** is rate‐determining.

**FIGURE 3:**
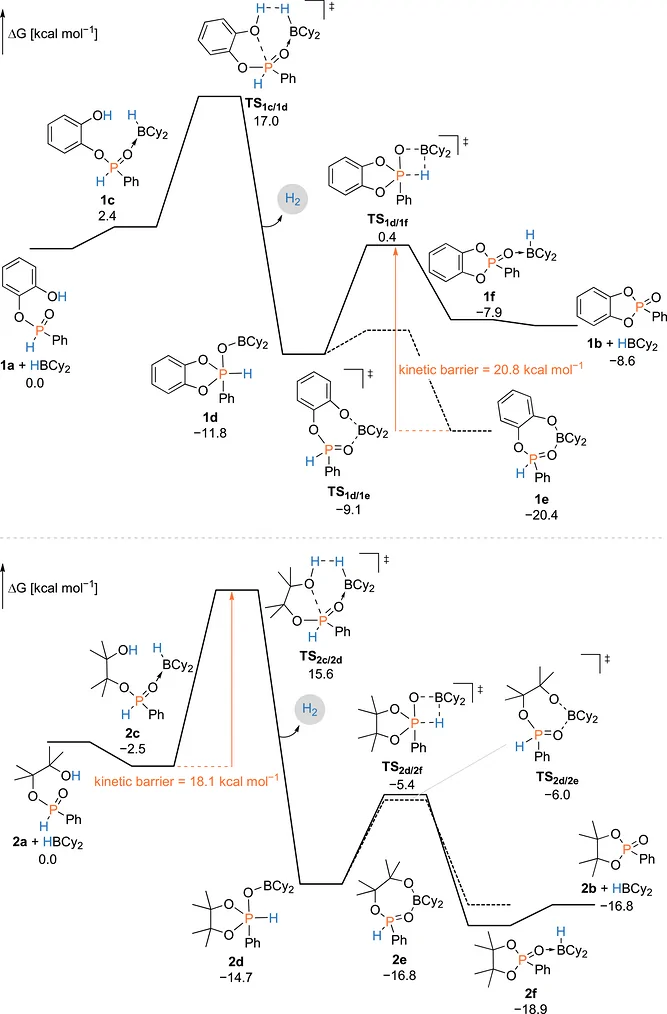
Computed potential Gibbs energy surfaces for the borane‐catalyzed hydrogen release from **1a** and **2a**. All calculations were performed at the SMD(THF)‐ωB97M‐V/def2‐QZVPP//CPCM(THF)‐r^2^SCAN‐3c level.

To prove experimentally the computational prediction that the B─H bond is critical for the hydrogen evolution, we performed a reaction with tricyclohexylborane, the closest congener without this functionality, as catalyst. Only traces of the dehydrogenation product **2b** were observed (see Table SI 3), showcasing the important role of the B─H functionality. We assume that diboroxane **7** can undergo a transesterification with the O─H group of the phenylphosphinate esters, generating the borinic acid esters **1e** and **2e,** which were proposed as intermediates in our DFT study (Figure 3) [36, 37]. In this way, **7** is an entry point to the catalytic cycle, which explains why it is active in the dehydrogenation of **1a** and **2a**. To further validate the computed mechanisms, we attempted to identify possible reaction intermediates experimentally. According to the computational study, the resting state of the dehydrogenation of **1a** is the borane‐substituted phenylphosphinate ester **1e**, which should also be accessible by the reaction of phosphine oxide **1b** with dicyclohexylborane **6** (Figure 3, top). Indeed, when **1b** was reacted with one equivalent of **6**, formation of a new species was observed by ^31^P NMR, which displayed a coupling pattern indicative of a P─H bond (27.89 (dt, *J* = 597.0, 14.9 Hz)) (Scheme 10A). The corresponding ^11^B NMR resonance at 40.06 ppm is typical for a trivalent borane, indicating that there is no strong coordination to the P═O group [38]. According to additional calculations, the closed form **1e** is in equilibrium with its open form **1g**. The conversion between these two forms is almost thermoneutral and has a low activation barrier of 4.0 kcal mol^−1^ (Scheme 10B), which explains the observed chemical shift in the ^11^B NMR spectrum. **1g** was found to catalyze the dehydrogenation of phenylphosphinate ester **1a** itself, supporting our assumption that mixed phenylphosphinate ester‐borinic acid esters are part of the catalytic cycle (see Chapter S7.3.2).

**SCHEME 10:**
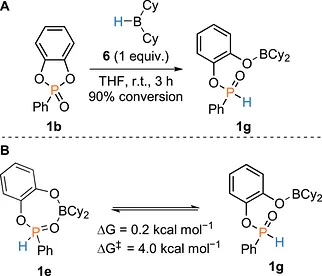
(A) Formation of the resting state **1g** of the catalytic cycle from the reaction between oxide **1b** and dicyclohexylborane **6**. (B) Computational study of the equilibrium between the cyclic (**1e**) and open form **1g** of the intermediate.

When a corresponding stoichiometric reaction between **2b** and borane **6** was carried out, no evidence of the formation of a new species was found by NMR. This indicates that the reaction of **2b** with borane **6** has no driving force, as predicted by the computations (Figure 3, bottom). The outcome of these stoichiometric reactions is thus in favorable agreement with the results of the computational study.

## 3 Conclusion

In summary, we report the observation that simple dioxaphosphole **1** and dioxaphospholane **2** display a high reactivity toward water, forming the corresponding phenylphosphinate esters **1a** and **2a** with stoichiometric amounts of water at room temperature within minutes. In an efficient borane‐catalyzed process, hydrogen can be released from these phenylphosphinate esters. Although traces of free water impede the catalytic reaction, a robust protocol was established, which allows a one‐pot dehydrogenation of water at a slightly elevated temperature of 60°C. Notably, formation of the phenylphosphinate esters **1a** and **2a** takes place already upon exposure of their parent dioxaphospholes and ‐phospholanes to air. We could demonstrate that **2a**, formed by water activation from atmospheric moisture, can also be dehydrogenated under the reported conditions, allowing atmospheric water to be directly integrated into the catalytic hydrogen formation. Mechanistic studies indicate that the borane‐catalyzed dehydrogenation of **1a** and **2a** proceeds via borane‐mediated hydrogen liberation and a retro‐hydroboration of the resulting phosphinate, which regenerates the borane catalyst. The properties of dioxaphosphole **1** and dioxaphospholane **2**, particularly their high reactivity toward water and the possibility of using the phenylphosphinate esters resulting from this reaction in a borane‐catalyzed hydrogen release, could be useful for the development of main‐group‐based systems for applications related to hydrogen storage and transfer hydrogenation.

## Author Contributions

**Niklas Koch**: investigation, conceptualization, writing – original draft, writing – review and editing, data curation. **Felix Wech**: conceptualization, investigation, writing – review and editing, data curation. **Tonin Leautier**: investigation, data curation, writing – review and editing. **Johanna Osterbrink**: investigation, data curation, writing – review and editing. **Urs Gellrich**: conceptualization, investigation, writing – original draft, writing – review and editing, project administration, supervision.

## Conflicts of Interest

The authors declare no conflicts of interest.

## Supporting information



The authors have cited additional references within the Supporting Information [39, 40, 41, 42, 43, 44, 45, 46, 47, 48, 49, 50, 51, 52, 53, 54, 55, 56, 57, 58, 59, 60, 61, 62, 63].**Supporting File**: anie74916‐sup‐0001‐SuppMat.docx.

## Data Availability

The data that supports the findings of this study are available in the Supporting Information of this article.
