# Supplementary material for: Borane‐Catalyzed Water Dehydrogenation at Dioxaphospholes and ‐Phospholanes
Source: Angew Chem Int Ed Engl. 2026 Sep 26;65(46):e3462462. doi: 10.1002/anie.3462462 (PMC13749294; doi:10.1002/anie.3462462)
Supplement: Supplementary file 1 [file ANIE-65-e3462462-s001.docx]

Supporting Information

**Borane-catalyzed Water Dehydrogenation at Dioxaphospholes and ‑phospholanes**

Niklas Koch, Felix Wech, Tonin Leautier, Johanna Osterbrink, Urs Gellrich

Table of contents

[1	General specifications	3](#_Toc241059125)

[2	Synthetic procedures	4](#_Toc241059126)

[2.1	Synthesis of dioxaphospholes and -phospholanes	4](#_Toc241059127)

[2.1.1	2-Phenyl-1,3,2-benzodioxaphosphole **1**	4](#_Toc241059128)

[2.1.2	4,4,5,5-Tetramethyl-2-phenyl-1,3,2-dioxaphospholane **2**	4](#_Toc241059129)

[2.2	Synthesis of phenylphosphinate esters	4](#_Toc241059130)

[2.2.1	General procedure	4](#_Toc241059131)

[2.2.2	2-Hydroxyphenyl phenylphosphinate **1a**	5](#_Toc241059132)

[2.2.3	Deuterated 2-hydroxyphenyl phenylphosphinate **1a-*d*_2_**	6](#_Toc241059133)

[2.2.4	3-Hydroxy-2,3-dimethylbutan-2-yl phenylphosphinate **2a**	7](#_Toc241059134)

[2.2.5	Deuterated 3-hydroxy-2,3-dimethylbutan-2-yl phenylphosphinate **2a-*d*_2_**	8](#_Toc241059135)

[2.3	Independent synthesis of 2-phenyl-1,3,2-benzodioxaphosphole 2-oxide **1b**	9](#_Toc241059136)

[3	Formation of phenylphosphinate esters **1a** and **2a** from moisture	10](#_Toc241059137)

[3.1	NMR spectra obtained after exposure of **1** to air	11](#_Toc241059138)

[3.2	NMR spectra obtained after exposure of **2** to air	14](#_Toc241059139)

[4	NMR studies of the water activation	16](#_Toc241059140)

[4.1	NMR experiments with methanol as surrogate	16](#_Toc241059141)

[4.2	Low temperature NMR studies on the water activation	18](#_Toc241059142)

[5	Dehydrogenation experiments	18](#_Toc241059143)

[5.1	Unsuccessful dehydrogenation with pyridonate borane **3**	18](#_Toc241059144)

[5.2	Successful dehydrogenation with pyridonate borane **4**	21](#_Toc241059145)

[5.3	Screening experiments for catalytic dehydrogenations	22](#_Toc241059146)

[5.3.1	NMR spectra of screening reactions	23](#_Toc241059147)

[5.4	Preparative scale dehydrogenations	28](#_Toc241059148)

[5.4.1	Characterization of **1b**	28](#_Toc241059149)

[5.4.2	Characterization of **2b**	29](#_Toc241059150)

[5.5	Hydrogen measurement via GC	29](#_Toc241059151)

[6	In situ reactions	30](#_Toc241059152)

[6.1	In situ water activation with subsequent dehydrogenation	30](#_Toc241059153)

[6.2	In situ reaction from moisture	32](#_Toc241059154)

[7	Mechanistic experiments	34](#_Toc241059155)

[7.1	Influence of trace water on the reaction progress	34](#_Toc241059156)

[7.2	Studies on the catalyst deactivation	34](#_Toc241059157)

[7.2.1	Synthesis of 1,1,3,3-tetracyclohexyldiboroxane **7**	35](#_Toc241059158)

[7.2.2	1,1,3,3-Tetracyclohexyldiboroxane **7** as catalyst for the dehydrogenation of phenylphosphinate esters **1a** and **2a**	37](#_Toc241059159)

[7.3	Identification of the resting state of the catalytic dehydrogenation	38](#_Toc241059160)

[7.3.1	Formation of the resting state **1g**	38](#_Toc241059161)

[7.3.2	Dehydrogenation of phenylphosphinate ester **1a** catalyzed by **1g**	43](#_Toc241059162)

[7.3.3	Formation of the resting state **2f**	44](#_Toc241059163)

[8	Kinetic measurements	46](#_Toc241059164)

[8.1	Sample preparation	46](#_Toc241059165)

[8.2	NMR data acquisition and evaluation	46](#_Toc241059166)

[8.2.1	Influence of relaxation delay on the integration	47](#_Toc241059167)

[8.3	Variable Time Normalization Analysis (VTNA)	47](#_Toc241059168)

[9	NMR spectra of isolated compounds	49](#_Toc241059169)

[9.1	NMR spectra of dioxaphospholes and phospholanes	49](#_Toc241059170)

[9.1.1	NMR spectra of 2-phenyl-1,3,2-benzodioxaphosphole **1**	49](#_Toc241059171)

[9.1.2	NMR spectra of 4,4,5,5-tetramethyl-2-phenyl-1,3,2-dioxaphospholane **2**	50](#_Toc241059172)

[9.2	NMR spectra of phenylphosphinate esters	54](#_Toc241059173)

[9.2.1	NMR spectra of 2-hydroxyphenyl phenylphosphinate **1a**	54](#_Toc241059174)

[9.2.2	NMR spectra of deuterated 2-hydroxyphenyl phenylphosphinate **1a-*d*_2_**	57](#_Toc241059175)

[9.2.3	NMR spectra of 3-hydroxy-2,3-dimethylbutan-2-yl phenylphosphinate **2a**	61](#_Toc241059176)

[9.2.4	NMR spectra of 3-hydroxy-2,3-dimethylbutan-2-yl phenylphosphinate **2a-*d*_2_**	64](#_Toc241059177)

[9.3	NMR spectra of phosphine oxides	68](#_Toc241059178)

[9.3.1	NMR spectra of **1b** obtained from preparative dehydrogenation	68](#_Toc241059179)

[9.3.2	NMR spectra of **2b** obtained from preparative dehydrogenation	69](#_Toc241059180)

[9.3.3	NMR spectra of independently synthesised **1b**	72](#_Toc241059181)

[9.4	NMR spectra of pyridonate borane **4**	75](#_Toc241059182)

[10	X-ray analysis	78](#_Toc241059183)

[10.1	Crystallographic data of 2-hydroxyphenyl phenylphosphinate **1a**	78](#_Toc241059184)

[10.2	Crystallographic data of 3-hydroxy-2,3-dimethylbutan-2-yl phenylphosphinate **2a**	84](#_Toc241059185)

[11	Computational details	89](#_Toc241059186)

[11.1	Energies and thermochemical corrections	89](#_Toc241059187)

[11.2	Cartesian Coordinates	90](#_Toc241059188)

[12	References	115](#_Toc241059189)

# 1 General specifications

All manipulations with air- and moisture-sensitive compounds were carried out under a nitrogen atmosphere using standard Schlenk and glovebox techniques.

6-*Tert*-butyl-2-pyridone was synthesised from 3,3’-dimethyl-2-butanone according to a literature procedure.^[1]^

B(C_6_F_5_)_3_ was synthesised from boron trifluoride etherate according to a literature procedure.^[2]^ Piers' Borane was synthesised from B(C_6_F_5_)_3_ according to a literature procedure.^[3]^

Dicyclohexylborane was synthesised from borane dimethylsulfide and cyclohexene according to a literature procedure.^[4,5]^

All dry, non-deuterated solvents were purchased from Acros Organics or Sigma Aldrich in a sealed bottle with a septum and stored over molecular sieves. Deuterated solvents were either distilled under inert conditions or degassed by three freeze-pump-thaw cycles and stored in a glovebox over molecular sieves. Demineralized water and deuterium oxide were degassed by bubbling an argon flow through them.

NMR spectra were recorded on Bruker Avance II 200 MHz, Bruker Avance III HD 400 MHz, Bruker Avance II 400 MHz, Varian Inova 500 MHz, and Bruker Avance III HD 600 MHz spectrometers. ^1^H and ^13^C NMR chemical shifts are referenced to residual solvent resonance peaks. ^31^P NMR chemical shifts are referenced externally to 85% H_3_PO_4_/D_2_O. Unless otherwise noted, ^31^P NMRs were recorded with a relaxation delay of 2 s. The T1-values of the phenylphosphinate esters **1a** and **2a** and the phosphine oxides **1b** and **2b** were measured and are given in the table below. These values are sufficiently close to determine conversions based on the integration of the ^31^P{^1^H} NMR (see chapter 8.2.1 for more details).

Table SI 1: T1-values of phenylphosphinate esters **1a** and **2a** and phosphine oxides **1b** and **2b**.

| **Compound** | **T1 / s** |
| --- | --- |
| **1a** | 3.59 |
| **1b** | 4.46 |
| **2a** | 4.02 |
| **2b** | 5.62 |

Mass spectra were recorded on an ESI-MS Bruker Mikro-TOF or a Thermo Fisher Scientific Exactive Plus Orbitrap mass spectrometer. Samples were dissolved in acetonitrile.

IR spectra were recorded on a Bruker ALPHA Compact FT-IR spectrometer. Samples were analysed as solid or THF solutions.

# 2 Synthetic procedures

## 2.1 Synthesis of dioxaphospholes and -phospholanes

### 2.1.1 2-Phenyl-1,3,2-benzodioxaphosphole 1

2-Phenyl-1,3,2-benzodioxaphosphole was synthesised according to a modified literature procedure.^[6]^

Catechol (11.0 g, 100 mmol, 1.00 equiv.) was dried under dynamic vacuum and then dissolved in dry diethyl ether (100 mL). Pyridine (16.2 mL, 15.9 g, 200 mmol, 2.00 equiv.) was added, and the solution was cooled to 0 °C with an ice bath. Dichlorophenylphosphine (13.5 mL, 17.9 g, 100 mmol, 1.00 equiv.) was added dropwise over 15 min. The reaction mixture was stirred at 0 °C for 1 h, then at r.t. for 2 h. The resulting suspension was filtered through a Schlenk frit, and the filtrate was concentrated in vacuo. The crude product was distilled (0.2 mbar, 70 °C) to obtain 2-phenyl-1,3,2-benzodioxaphosphole as a colorless oil (10.9 g, 50.4 mmol, 50%). The product was stored inside the Glovebox at −35 °C to avoid decomposition.

^1^H NMR (400 MHz, CDCl_3_): δ 7.51 (tt, *J* = 6.7, 1.4 Hz, 2H, C*H*_ph_), 7.45 – 7.39 (m, 1H, C*H*_ph_), 7.39 – 7.32 (m, 2H, C*H*_ph_), 7.08 (dd, *J* = 5.8, 3.4 Hz, 2H, C*H*_cat_), 6.94 (dd, *J* = 5.8, 3.4 Hz, 2H, C*H*_cat_).

^31^P{^1^H} NMR (162 MHz, CDCl_3_): δ 180.07.

NMR data are consistent with the literature.^[7]^

### 2.1.2 **4,4,5,5-Tetramethyl-2-phenyl-1,3,2-dioxaphospholane 2**

4,4,5,5-Tetramethyl-2-phenyl-1,3,2-dioxaphospholane was synthesised on the basis of a literature procedure for 2-phenyl-1,3,2-benzodioxaphosphole.^[6]^

Pinacol (5.91 g, 50.0 mmol, 1.00 equiv.) was dissolved in dry diethyl ether (50 mL). Pyridine (8.11 mL, 7.95 g, 100 mmol, 2.00 equiv.) was added, and the solution was cooled to 0 °C with an ice bath. Dichlorophenylphosphine (6.76 mL, 8.95 g, 50.0 mmol, 1.00 equiv.) was added dropwise over 15 min. The reaction mixture was stirred at 0 °C for 1 h, then at r.t. for 2 h. The resulting suspension was filtered through a Schlenk frit, and the filtrate was concentrated in vacuo. The crude product was purified via Kugelrohr sublimation (0.1 – 0.2 mbar, 120 °C) to obtain 4,4,5,5-tetramethyl-2-phenyl-1,3,2-dioxaphosphole as a white solid (6.59 g, 29.4 mmol, 59%).

^1^H NMR (400 MHz, C_6_D_6_): δ 7.68 – 7.59 (m, 2H, C*H*_ph_), 7.17 – 7.08 (m, 2H, C*H*_ph_), 7.09 – 7.03 (m, 1H, C*H*_ph_), 1.14 (s, 6H, C*H*_3_), 0.94 (s, 6H, C*H*_3_).

^13^C NMR (101 MHz, C_6_D_6_): δ 148.20 (d, *J* = 57.1 Hz, *C*_q,ph_), 129.53 (*C*H_ph_), 128.45 (d, *J* = 3.0 Hz, *C*H_ph_), 128.12 (*C*H_ph_), 84.76 (d, *J* = 6.4 Hz, *C*(CH_3_)_2_), 25.21 (d, *J* = 4.0 Hz, *C*H_3_), 24.62 (d, *J* = 1.1 Hz, *C*H_3_).

^31^P{^1^H} NMR (162 MHz, C_6_D_6_): δ 172.07.

Previously reported syntheses of this compound do not include complete NMR data.^[8]^

## 2.2 Synthesis of phenylphosphinate esters

### 2.2.1 General procedure

In a Schlenk tube equipped with a J. Young valve, the dioxaphosphole or -phospholane (5.00 mmol, 1.00 equiv.) was dissolved in acetonitrile (dry, 10 mL). Water or deuterium oxide (5.00 mmol, 1.00 equiv.) was added, and the tube was sealed. After 10 min stirring at r.t., the solvent was removed in vacuo. The colorless solid residue was dissolved in dry DCM (3 mL) and transferred into a vial. The tube was rinsed with additional DCM (2 mL). The solvent was allowed to fully evaporate overnight to obtain the phenylphosphinate ester as colorless crystals. Additional purification was achieved by crystallization from DCM/*n*-hexane.

### 2.2.2 2-Hydroxyphenyl phenylphosphinate 1a

Scheme SI 1: Synthesis of 2-hydroxyphenyl phenylphosphinate **1a**.

The product was obtained as colorless crystals (1.16 g, 4.95 mmol, 99%) from 2-phenyl-1,3,2-benzodioxaphosphole **1** (1.08 g, 5.00 mmol, 1.00 equiv.) and water (90.1 μL, 5.00 mmol, 1.00 equiv.) according to the general procedure.

^1^H NMR (400 MHz, CDCl_3_): δ 8.10 (s, 1H, O*H*), 7.87 (d, *J* = 585.3 Hz, 1H, P*H*), 7.95 – 7.84 (m, 2H, C*H*_ph_), 7.73 – 7.63 (m, 1H, C*H*_ph_), 7.57 (tdd, *J* = 6.5, 3.6, 1.3 Hz, 2H, C*H*_ph_), 7.07 – 7.02 (m, 2H, C*H*_cat_), 7.02 – 6.95 (m, 1H), 6.82 – 6.77 (m, 1H, C*H*_cat_).

^13^C {^1^H} NMR (101 MHz, CDCl_3_): δ 147.86 (d, *J* = 3.5 Hz, *C*_q,cat_), 139.19 (d, *J* = 9.9 Hz, *C*OH), 134.26 (d, *J* = 3.0 Hz, *C*H_ph_), 131.21 (d, *J* = 12.5 Hz, *C*H_ph_), 129.21 (d, *J* = 14.4 Hz, *C*H_ph_), 127.55 (d, *J* = 131.8 Hz, *C*_q,ph_), 126.99 (d, *J* = 1.6 Hz, *C*H_cat_), 121.87 (d, *J* = 4.6 Hz, *C*H_cat_), 120.87 (d, *J* = 1.2 Hz, *C*H_cat_), 119.41 (d, *J* = 1.6 Hz, *C*H_cat_).

^31^P NMR (162 MHz, CDCl_3_): δ 31.49 (dt, *J* = 585.3, 14.9 Hz).

HRMS (ESI) m/z	[M+H^+^] calc. for C_12_H_12_O_3_P^+^: 235.0519;	found [M+H^+^]: 235.0512	[M+Na^+^] calc. for C_12_H_11_O_3_PNa^+^: 257.0338;	found [M+Na^+^]: 257.0338

IR (THF solution, cm^−1^): *ṽ* = 3157 (br, OH), 2388 (PH).

Figure SI 1: IR spectrum of 2-hydroxyphenyl phenylphosphinate **1a**.

### 2.2.3 Deuterated 2-hydroxyphenyl phenylphosphinate 1a-*d*_2_

Scheme SI 2: Synthesis of deuterated 2-hydroxyphenyl phenylphosphinate **1a-d_2_**.

The product was obtained as colorless crystals (1.09 g, 4.62 mmol, 93%) from 2-phenyl-1,3,2-benzodioxaphosphole **1** (1.08 g, 5.00 mmol, 1.00 equiv.) and deuterium oxide (90.8 μL, 5.00 mmol, 1.00 equiv.) according to the general procedure.

^1^H NMR (400 MHz, CDCl_3_): 7.94 – 7.83 (m, 2H, C*H*_ph_), 7.72 – 7.62 (m, 1H, C*H*_ph_), 7.60 – 7.52 (m, 2H, C*H*_ph_), 7.09 – 7.00 (m, 2H, *C*H_cat_), 6.98 (ddt, *J* = 8.0, 1.8, 0.9 Hz, 1H, *C*H_cat_), 6.84 – 6.75 (m, 1H, *C*H_cat_).

^2^D NMR: (61 MHz, CD_3_CN): δ 7.93 (d, *J* = 90.8 Hz, P*D*), 7.49 (s, O*D*).

^13^C {^1^H} NMR (101 MHz, CDCl_3_): δ 147.61 (d, *J* = 3.4 Hz, *C*_q,cat_), 138.96 (d, *J* = 9.9 Hz, *C*OD), 134.13 (t, *J* = 2.9 Hz, *C*H_ph_), 131.08 (d, *J* = 12.5 Hz, *C*H_ph_), 129.07 (d, *J* = 14.3 Hz, *C*H_ph_), 126.86 (d, *J* = 3.8 Hz, *C*H_cat_), 121.74 (dd, *J* = 4.7, 1.7 Hz, *C*H_cat_), 120.76 (d, *J* = 4.0 Hz, *C*H_cat_), 119.23 (d, *J* = 9.3 Hz, *C*H_cat_).

Note that the phenyl-carbon directly attached to the phosphorus could not be observed.

^31^P{^1^H} NMR (162 MHz, CDCl_3_): δ 31.24 (t, *J* = 89.4 Hz).

^31^P NMR (162 MHz, CDCl_3_): δ 31.24 (tt, *J* = 89.4, 14.8 Hz).

HRMS (ESI) m/z	[M+Na^+^] calc. for C_12_H_10_DO_3_PNa^+^: 258.0401;	found [M+Na^+^]: 258.0403

Note that only the mass of the mono-deuterated compound could be observed.

IR (solid, cm^−1^): *ṽ* = 2305 (br, OD), 1740 (PD).

Figure SI 2: IR spectrum of deuterated 2-hydroxyphenyl phenylphosphinate **1a-d_2_**.

### 2.2.4 3-Hydroxy-2,3-dimethylbutan-2-yl phenylphosphinate 2a

Scheme SI 3: Synthesis of 3-hydroxy-2,3-dimethylbutan-2-yl phenylphosphinate **2a**.

The product was obtained as colorless crystals (1.09 g, 4.50 mmol, 90%) from 4,4,5,5-tetramethyl-2-phenyl-1,3,2-dioxaphospholane **2** (1.12 g, 5.00 mmol, 1.00 equiv.) and water (90.1 μL, 5.00 mmol, 1.00 equiv.) according to the general procedure.

^1^H NMR (400 MHz, CDCl_3_): 7.78 (ddd, *J* = 14.2, 8.3, 1.4 Hz, 2H, C*H*_ph_), 7.73 (d, *J* = 559.4 Hz, 1H, P*H*), 7.64 – 7.56 (m, 1H, C*H*_ph_), 7.51 (tdd, *J* = 6.8, 3.6, 1.3 Hz, 2H, C*H*_ph_), 3.81 (s, 1H, O*H*), 1.65 (s, 3H, C*H*_3_), 1.50 (s, 3H, C*H*_3_), 1.31 (s, 3H, C*H*_3_), 1.26 (s, 3H, C*H*_3_).

^13^C {^1^H} NMR (101 MHz, CDCl_3_): δ 133.02 (d, *J* = 3.0 Hz, *C*H_ph_), 130.91 (d, *J* = 12.0 Hz, *C*H_ph_), 130.86 (d, *J* = 139.8 Hz, *C*_q,ph_), 128.89 (d, *J* = 14.2 Hz, *C*H_ph_), 92.43 (d, *J* = 9.3 Hz, *C*(CH_3_)_2_), 74.42 (d, *J* = 2.5 Hz, *C*(CH_3_)_2_), 25.42, 25.36, 24.94, 23.29 (d, *J* = 5.0 Hz, all *C*H_3_).

^31^P NMR (162 MHz, CDCl_3_): δ 18.75 (dt, *J* = 559.5, 14.5 Hz).

HRMS (ESI) m/z	[M+Na^+^] calc. for C_12_H_19_O_3_PNa^+^: 265.0964;	found [M+Na^+^]: 265.0967

IR (THF solution, cm^−1^): *ṽ* = 3387 (br, OH), 2358 (PH).

Figure SI 3: IR spectrum of 3-hydroxy-2,3-dimethylbutan-2-yl phenylphosphinate **2a**.

### 2.2.5 Deuterated 3-hydroxy-2,3-dimethylbutan-2-yl phenylphosphinate 2a-*d*_2_

Scheme SI 4: Synthesis of deuterated 3-hydroxy-2,3-dimethylbutan-2-yl phenylphosphinate **2a**-d_2_.

The product was obtained as colorless crystals (1.09 g, 4.50 mmol, 64%) from 4,4,5,5-tetramethyl-2-phenyl-1,3,2-dioxaphospholane **2** (1.12 g, 5.00 mmol, 1.00 equiv.) and deuterium oxide (90.8 μL, 5.00 mmol, 1.00 equiv.) according to the general procedure.

^1^H NMR (400 MHz, CDCl_3_): 7.82 – 7.73 (m, 2H, C*H*_ph_), 7.64 – 7.57 (m, 1H, C*H*_ph_), 7.55 – 7.48 (m, 2H, C*H*_ph_), 1.65 (s, 3H, C*H*_3_), 1.50 (d, *J* = 1.3 Hz, 3H, C*H*_3_), 1.31 (s, 3H, C*H*_3_), 1.26 (s, 3H, C*H*_3_).

^2^D NMR: (61 MHz, CD_3_CN): δ 7.74 (d, *J* = 86.1 Hz, P*D*), 3.78 (s, O*D*).

^13^C {^1^H} NMR (101 MHz, CDCl_3_): δ 133.04 (d, *J* = 2.9 Hz, *C*H_ph_), 130.92 (d, *J* = 11.8 Hz, *C*H_ph_), 128.89 (d, *J* = 14.1 Hz, *C*H_ph_), 92.41 (d, *J* = 9.5 Hz, *C*(CH_3_)_2_), 74.35 (*C*(CH_3_)_2_), 25.40 (d, *J* = 6.4 Hz), 25.33, 24.91, 23.27 (d, *J* = 5.2 Hz, all *C*H_3_).

Note that the phenyl-carbon directly attached to the phosphorus could not be observed.

^31^P{^1^H} NMR (162 MHz, CDCl_3_): δ 18.39 (t, *J* = 86.2 Hz).

HRMS (ESI) m/z	[M+Na^+^] calc. for C_12_H_18_DO_3_PNa^+^: 266.1027;	found [M+Na^+^]: 266.1027

Note that only the mass of the mono-deuterated compound could be observed.

IR (solid, cm^−1^): *ṽ* = 2480 (br, OD), 1753 (PD).

Figure SI 4: IR spectrum of deuterated 3-hydroxy-2,3-dimethylbutan-2-yl phenylphosphinate **2a-d_2_**.

## 2.3 Independent synthesis of 2-phenyl-1,3,2-benzodioxaphosphole 2-oxide 1b

Scheme SI 5: Reaction sequence for the independent synthesis of **1b**.

Phenylphosphonic dichloride was synthesised from dichlorophenylphosphine and dimethyl sulfoxide according to a literature procedure.^[9]^

2-Phenyl-1,3,2-benzodioxaphosphole 2-oxide **1b** was synthesised according to the procedure described for 2-phenyl-1,3,2-benzodioxaphosphole **1** (see Chapter 2.1).

Catechol (0.77 g, 7.0 mmol, 1.0 equiv.) was dissolved in dry diethyl ether (40 mL). Pyridine (1.14 mL, 1.11 g, 14 mmol, 2.0 equiv.) was added, and the solution was cooled to 0 °C in an ice bath. Phenylphosphonic dichloride (0.99 mL, 1.36 g, 7.0 mmol, 1.0 equiv.) was added dropwise. The reaction mixture was stirred at 0 °C for 1 h, then at r.t. for 2 h. The resulting suspension was filtered through a Schlenk frit, and the filtrate was concentrated in vacuo. The obtained brownish solid residue was subjected to a Kugelrohr sublimation (0.3 mbar, 175 °C) to obtain 2-phenyl-1,3,2-benzodioxaphosphole 2-oxide as a colorless oil, which slowly solidified to a crystalline solid (0.41 g, 1.77 mmol, 25%).

^1^H NMR (600 MHz, CDCl_3_): δ 7.81 (ddq, *J* = 14.9, 6.8, 1.3 Hz, 2H, C*H*_ph_), 7.65 (ddq, *J* = 9.0, 7.3, 1.4 Hz, 1H, C*H*_ph_), 7.53 – 7.47 (m, 2H, C*H*_ph_), 7.14 (dtd, *J* = 5.4, 3.9, 1.1 Hz, 2H, C*H*_cat_), 7.07 (dd, *J* = 5.9, 3.5 Hz, 2H, C*H*_cat_).

^13^C NMR (151 MHz, CDCl_3_) δ 145.07 (*C*_q,cat_), 134.60 (d, *J* = 3.3 Hz, *C*H_ph_), 132.36 (d, *J* = 11.6 Hz, *C*H_ph_), 129.03 (d, *J* = 16.6 Hz), *C*H_ph_, 124.28 (d, *J* = 189.3 Hz, *C*_q,ph_), 123.91 (*C*H_cat_), 112.77 (d, *J* = 10.5 Hz, *C*H_cat_).

^31^P{^1^H} NMR (243 MHz, CDCl_3_): δ 36.29.

^31^P NMR (243 MHz, CDCl_3_) δ 36.29 (t, *J* = 15.3 Hz).

NMR data are consistent with the literature.^[10]^

# 3 Formation of phenylphosphinate esters 1a and 2a from moisture

Scheme SI 6: Formation of phenylphosphinate esters **1a** and **2a** upon exposure to air. Formation of the corresponding oxides **1b** and **2b** as side products is observed.

Procedure:

Solid portions of 2-phenyl-1,3,2-benzodioxaphosphole **1** and 4,4,5,5-tetramethyl-2-phenyl-1,3,2-dioxaphospholane **2** (0.05 mmol, 10.8 mg or 11.2 mg, respectively) were placed in open vials in a ventilated fumehood with a humidity of approximately 35%. After time intervals specified in the table below, the contents of the vials were dissolved in 0.5 mL chloroform-*d*_1_, and NMR spectra of the obtained solutions were recorded.

Table SI 2: Ratios of starting materials, phenylphosphinate esters, and oxides after exposure to air for different time intervals.

| Starting material | *t* / h | 1 *or* 2 | 1a *or* 2a | 1b *or* 2b |
| --- | --- | --- | --- | --- |
| **1** | 0.5 | 15% | 78% | 8% |
| **1** | 1 | 12% | 79% | 9% |
| **1** | 2 | 0% | 85% | 9%^[a]^ |
| **2** | 0.5 | 0% | 92% | 8% |
| **2** | 1 | 0% | 93% | 7% |
| **2** | 24 | 0% | 94% | 6% |

Ratios were obtained by integration of the ^31^P{^1^H} NMR spectra. [a] Formation of 5% of a new species at −10.6 ppm is observed.

In the case of **1**, at longer exposure times, decomposition into two new species with a chemical shift of 24.44 (dt, *J* = 574.8, 14.4 Hz) and 19.16 (t, *J* = 14.5 Hz) is observed, which is completed after 24 h. In contrast, samples of **2** analysed after 24 h exposure do not significantly differ from those obtained after 0.5 or 1 h.

## 3.1 NMR spectra obtained after exposure of 1 to air

Figure SI 5: ^31^P{^1^H} NMR spectrum obtained after exposure of 2-phenyl-1,3,2-benzodioxaphosphole **1** to air for 0.5 h (243 MHz, chloroform-d_1_).

Figure SI 6: ^31^P{^1^H} NMR spectrum obtained after exposure of 2-phenyl-1,3,2-benzodioxaphosphole **1** to air for 1 h (243 MHz, chloroform-d_1_).

Figure SI 7: ^31^P{^1^H} NMR spectrum obtained after exposure of 2-phenyl-1,3,2-benzodioxaphosphole **1** to air for 2 h (202 MHz, chloroform-d_1_).

Figure SI 8: ^31^P{^1^H} NMR spectrum obtained after exposure of 2-phenyl-1,3,2-benzodioxaphosphole **1** to air for 6 h (243 MHz, chloroform-d_1_). Decomposition into unidentified side products is partially observed. The excerpt of the ^31^P NMR (top right) shows the coupling pattern of the different products.

Figure SI 9: ^31^P{^1^H} NMR spectrum obtained after exposure of 2-phenyl-1,3,2-benzodioxaphosphole **1** to air for 24 h (243 MHz, chloroform-d_1_). Decomposition into unidentified side products is complete. The excerpt of the ^31^P NMR (top left) shows the coupling pattern of the decomposition products.

## 3.2 NMR spectra obtained after exposure of 2 to air

Figure SI 10: ^31^P{^1^H} NMR spectrum obtained after exposure of 4,4,5,5-tetramethyl-2-phenyl-1,3,2-dioxaphospholane **2** to air for 0.5 h (243 MHz, chloroform-d_1_).

Figure SI 11: ^31^P{^1^H} NMR spectrum obtained after exposure of 4,4,5,5-tetramethyl-2-phenyl-1,3,2-dioxaphospholane **2** to air for 1 h (243 MHz, chloroform-d_1_).

Figure SI 12: ^31^P{^1^H} NMR spectrum obtained after exposure of 4,4,5,5-tetramethyl-2-phenyl-1,3,2-dioxaphospholane **2** to air for 24 h (243 MHz, chloroform-d_1_).

# 4 NMR studies of the water activation

## 4.1 NMR experiments with methanol as surrogate

Scheme SI 7: Formation of a σ^5^‑phosphorane upon reaction of 4,4,5,5-tetramethyl-2-phenyl-1,3,2-dioxaphospholane **2** with methanol.

To gain insights into the water activation by 4,4,5,5-tetramethyl-2-phenyl-1,3,2-dioxaphospholane **2**, we replaced water with methanol to suppress the isomerization to phenylphosphinate ester **2a**.

4,4,5,5-Tetramethyl-2-phenyl-1,3,2-dioxaphospholane **2** (56.1 mg, 0.25 mmol, 1 equiv.) was dissolved in acetonitrile-*d*_3_ (0.5 mL, 0.5 M) and the solution was transferred to an NMR tube equipped with a J. Young valve. Methanol (10.1 µL, 0.25 mmol, 1 equiv., dried over molecular sieves) was added to the tube. NMR spectra were recorded at ambient temperature. The formation of an equilibrium between **2** and the σ^5^‑phosphorane as described by Barrans *et al.* was observed.^[11]^ The ratio is approximately 70:30, favoring the methanol adduct.

^1^H NMR (500 MHz, CD_3_CN) δ 7.89 (ddt, *J* = 17.3, 7.0, 1.5 Hz, 2H, C*H*_ph_ **2∙MeOH**), 7.56 – 7.48 (m, 2H, , C*H*_ph_ **2∙MeOH**), 7.48 – 7.38 (m, 1H, , C*H*_ph_ **2∙MeOH** overlapping with C*H*_ph_ **2**), 6.23 (d, *J* = 567.1 Hz, 1H, P*H* **2∙MeOH**), 3.28 (s, 3H, C*H*_3_ of remaining MeOH), 3.24 (d, *J* = 20.8 Hz, 3H, POC*H*_3_ **2∙MeOH**), 1.32 (s, 3H, C*H*_3_ **2**), 1.19 (s, 3H, C*H*_3_ **2∙MeOH**), 1.13 (s, 3H, C*H*_3_ **2**, overlapping with the next signal), 1.12 (s, 3H, C*H*_3_ **2∙MeOH**).

^31^P{^1^H} NMR (202 MHz, CD_3_CN) δ 170.42 (**2**), -26.37 (**2∙MeOH**).

^31^P NMR (202 MHz, CD_3_CN) δ 170.41 (t, *J* = 5.3 Hz, **2**), -24.63 – -28.05 (m, **2∙MeOH**).

Figure SI 13: ^1^H NMR spectrum of the reaction of 4,4,5,5-tetramethyl-2-phenyl-1,3,2-dioxaphospholane **2** with methanol (500 MHz, acetonitrile‑d_3_). The ratio of **2∙MeOH** and **2** is approximately 70:30.

Figure SI 14: ^31^P{^1^H} NMR spectrum of the reaction of 4,4,5,5-tetramethyl-2-phenyl-1,3,2-dioxaphospholane **2** with methanol (202 MHz, acetonitrile‑d_3_). Excerpts of the ^31^P NMR show the coupling patterns of both signals.

## 4.2 Low temperature NMR studies on the water activation

Solutions of 4,4,5,5-tetramethyl-2-phenyl-1,3,2-dioxaphospholane **2** (56.1 mg) and water (4.51 µL) were prepared in acetonitrile-*d*_3_ (2.0 mL, 0.125 M and 0.5 mL, 0.5 M). An aliquot of the solution of **2** (400 µL, 0.05 mmol, 1 equiv.) was transferred to an NMR tube. The tube was cooled in the NMR instrument to −30 °C, and NMR spectra were processed. After reaching the desired temperature, the tube was ejected from the instrument and placed in a cryostat at −30 °C to maintain the temperature. An aliquot of the water solution (100 µL, 0.05 mmol, 1 equiv.), likewise cooled to the same temperature, was added to the tube. After shaking the tube, it was inserted into the instrument again, and a measurement series of ^31^P NMR spectra was started. The reaction was slowly warmed to −10 °C in the instrument to achieve full conversion. Within this study, no evidence for the presence of the σ^5^‑phosphorane intermediate could be detected.

Figure SI 15: Stacked ^31^P NMR spectra of the addition of water to 4,4,5,5-tetramethyl-2-phenyl-1,3,2-dioxaphospholane **2** at −30 °C (202 MHz, acetonitrile‑d_3_). Complete conversion was achieved after warming to −10 °C (top).

# 5 Dehydrogenation experiments

## 5.1 Unsuccessful dehydrogenation with pyridonate borane 3

A solution of 6-*tert*-butyl-2-pyridone (15.1 mg, 0.10 mmol, 1.00 equiv.) in 5 mL *n*‑hexane was added to a vial containing Piers' borane (38.1 mg, 0.11 mmol, 1.10 equiv.). The mixture was shaken vigorously. The obtained solution was filtered through a syringe filter after 1 h, and the solvent was allowed to evaporate at r.t. The pyridonate borane **3** was obtained as an oily residue, which was taken up in 0.5 mL benzene-*d*_6_ and transferred into an NMR tube equipped with a J. Young valve. Analytical data are in accordance with those reported in the literature.^[12]^

2-Hydroxyphenyl phenylphosphinate **1a** (23.4 mg, 0.10 mmol, 1.00 equiv.) was added to the tube. At r.t., a shift in the ^31^P NMR to 42.97 ppm was observed, but a large coupling constant of *J* = 640.8 Hz revealed that the P-H bond is still present (Figure SI 16). Upon heating to 80 °C for 4 h, formation of **1** was observed, indicating water abstraction from **1a** by the pyridonate borane **3**. After a further 18 h at 80 °C, the starting material was fully consumed, and **1** (ca. 75%), 2-phenyl-1,3,2-benzodioxaphosphole 2‑oxide **1b** (ca. 14%) formed along with various minor products which were not further investigated (Figure SI 17).

Furthermore, formation of pentafluorobenzene is evident in the ^1^H NMR spectrum (δ  = 5.81 – 5.70 ppm (m, 1H)),^[13]^ indicating decomposition of the pyridonate borane **3** (Figure SI 18).

Figure SI 16: ^31^P NMR spectrum of the reaction of 2-hydroxyphenyl phenylphosphinate **1a** with the pyridonate borane **3** at r.t. (162 MHz, benzene‑d_6_). A low-field shift is observed compared to isolated **1a**.

Figure SI 17: ^31^P NMR spectrum of the reaction of 2-hydroxyphenyl phenylphosphinate **1a** with the pyridonate borane **3** after heating to 80 °C for 22 h (162 MHz, benzene‑d_6_). Approximately 75% of **1** and 14% of **1b** are observed.

Figure SI 18: ^1^H NMR spectrum of the reaction of 2-hydroxyphenyl phenylphosphinate **1a** with the pyridonate borane **3** after heating to 80 °C for 22 h (400 MHz, benzene‑d_6_). Formation of pentafluorobenzene is evident (see excerpt).

## 5.2 Successful dehydrogenation with pyridonate borane 4

A solution of 6-*tert*-butyl-2-pyridone (7.56 mg, 0.05 mmol, 1.00 equiv.) in 0.5 mL benzene-*d*_6_ was added to a vial containing dicyclohexylborane (8.91 mg, 0.05 mmol, 1.00 equiv.). Vigorous gas evolution occurred, and within 5 min, a clear solution of the pyridonate borane **4** was obtained, which was transferred into an NMR tube equipped with a J. Young valve.

^1^H NMR (400 MHz, C_6_D_6_): δ 7.10 (t, *J* = 7.8 Hz, 1H, C*H*_py_), 6.74 (dd, *J* = 7.6, 0.8 Hz, 1H, C*H*_py_), 6.46 (dd, *J* = 8.0, 0.7 Hz, 1H, C*H*_py_), 1.88 – 1.80 (m, 4H, C*H*_2_), 1.80 – 1.67 (m, 6H, C*H*_2_), 1.54 – 1.34 (m, 5H, C*H*_2_), 1.33 (s, 9H, *t*Bu), 1.31 – 1.20 (m, 5H, C*H*_2_).

^11^B NMR (128 MHz, C_6_D_6_): δ 53.47.

^13^C{^1^H} NMR (101 MHz, C_6_D_6_): δ 168.54, 162.02, 139.40 (*C*H_py_), 114.19 (*C*H_py_), 110.96 (*C*H_py_), 37.44 (*C*(CH_3_)_3_), 30.14 (C(*C*H_3_)_3_), 27.85 (*C*H_2_), 27.77 (*C*H_2_), 27.36 (*C*H_2_).

The 2-hydroxyphenyl phenylphosphinate **1a** (11.7 mg, 0.05 mmol, 1.00 equiv.) was added to the tube. Monitoring of the reaction at r.t. via ^31^P{^1^H} NMR indicated 90% conversion to 2-phenyl-1,3,2-benzodioxaphosphole 2-oxide **1b** after 6 h (Figure SI 19). The formation of dihydrogen is visible in the ^1^H NMR spectrum recorded after the reaction (Figure SI 20).

Figure SI 19: ^31^P{^1^H} NMR spectra of the stoichiometric reaction between 2-hydroxyphenyl phenylphosphinate **1a** and pyridonate borane **4** at r.t. after specified time intervals. (243 MHz, benzene-d_6_).

Figure SI 20: ^1^H NMR spectrum after the stoichiometric reaction between 2-hydroxyphenyl phenylphosphinate **1a** and pyridonate borane **4** at r.t., showing the formation of dihydrogen (600 MHz, benzene-d_6_).

A further equivalent of **1a** was added. Gas evolution was observed, and ^31^P NMR showed conversion to **1b**.

## 5.3 Screening experiments for catalytic dehydrogenations

Scheme SI 8: Screening experiments for the dehydrogenation of phenylphosphinate esters **1a** and **2a**.

General procedure:

The phenylphosphinate ester (0.500 mmol, 1.00 equiv.), the borane, and the co-catalyst (both 0.05 mmol, 10 mol%) were dissolved in THF (dry, 4 mL, 0.5 mL, and 0.5 mL, respectively) and added to a Schlenk tube equipped with a J. Young valve. The reaction was stirred at 1000 rpm for 6 h at the denoted temperature. The conversion to the phosphine oxides **1b** or **2b** was determined by ^31^P{^1^H} NMR spectroscopy using an aliquot of the reaction solution with a sealed glass capillary filled with benzene-*d*_6_.

If either the borane or the co-catalyst was kept off, the same procedure was applied, but the remaining catalyst component was dissolved in 1 mL of solvent.

Table SI 3: Screening results for the dehydrogenation of phenylphosphinate esters **1a** and **2a**.

| Entry | Starting material | Catalyst | *T* / °C | Conversion |
| --- | --- | --- | --- | --- |
| 1 | **1a** | **5 + 6** | 60 | Quant. |
| 2 | **2a** | **5 + 6** | 60 | Quant. |
| 3 | **1a** | **6** | 60 | Quant. |
| 4 | **2a** | **6** | 60 | Quant. |
| 5 | **1a** | None | 60 | Traces |
| 6 | **2a** | None | 60 | Traces |
| 7 | **1a** | **6** | 25 | Traces |
| 8 | **2a** | **6** | 25 | Quant. |
| 9 | **1a** | **5 + 6** | 25 | Traces |
| 10 | **2a** | BCy_3_ | 25 | Traces |

All reactions were carried out at a 0.5 mmol scale. Conversion determined from ^31^P{^1^H} NMR.

### 5.3.1 NMR spectra of screening reactions

Figure SI 21: ^31^P{^1^H} NMR of a reaction aliquot taken from the dehydrogenation of **1a** according to entry 1 (243 MHz, THF).

Figure SI 22: ^31^P{^1^H} NMR of a reaction aliquot taken from the dehydrogenation of **2a** according to entry 2 (243 MHz, THF).

Figure SI 23: ^31^P{^1^H} NMR of a reaction aliquot taken from the dehydrogenation of **1a** according to entry 3 (243 MHz, THF).

Figure SI 24: ^31^P{^1^H} NMR of a reaction aliquot taken from the dehydrogenation of **2a** according to entry 4 (243 MHz, THF).

Figure SI 25: ^31^P{^1^H} NMR of a reaction aliquot taken from the dehydrogenation of **1a** according to entry 5 (243 MHz, THF).

Figure SI 26: ^31^P{^1^H} NMR of a reaction aliquot taken from the dehydrogenation of **2a** according to entry 6 (243 MHz, THF).

Figure SI 27: ^31^P{^1^H} NMR of a reaction aliquot taken from the dehydrogenation of **1a** according to entry 7 (202 MHz, THF).

Figure SI 28: ^31^P{^1^H} NMR of a reaction aliquot taken from the dehydrogenation of **2a** according to entry 8 (243 MHz, THF).

Figure SI 29: ^31^P{^1^H} NMR of a reaction aliquot taken from the dehydrogenation of **1a** according to entry 9 (243 MHz, THF).

Figure SI 30: ^31^P{^1^H} NMR of a reaction aliquot taken from the dehydrogenation of **2a** according to entry 10 (202 MHz, THF).

## 5.4 Preparative scale dehydrogenations

General Procedure:

The phenylphosphinate ester **1a** or **2a** (1 mmol, 1 equiv.) was dissolved in dry THF (4 mL). A solution of dicyclohexylborane **6** (17.8 mg, 0.1 mmol, 10 mol%) in dry THF (1 mL) was added, and the reaction mixture was transferred into a tube equipped with a J. Young valve and sealed. The reaction was stirred for 6 h at 60 °C or room temperature for **1a** and **2a**, respectively.

### 5.4.1 Characterization of 1b

Note that phosphine oxide **1b** is not stable towards non-inert work-up conditions. Therefore, the solvent was removed in vacuo, the crude product was dissolved in dry dichloromethane (3 mL) and transferred into a flask. The solvent was removed in vacuo to obtain the phosphine oxide **1b** as a colorless oil (244 mg, 98% mass balance) from 2-hydroxyphenyl phenylphosphinate **1a** according to the general procedure from 4.4.

^1^H NMR (600 MHz, CDCl_3_): δ 7.87 – 7.80 (m, 2H, C*H*_ph_), 7.68 (tq, *J* = 7.2, 1.4 Hz, 1H, C*H*_ph_), 7.56 – 7.49 (m, 2H, C*H*_ph_), 7.17 (ddd, *J* = 5.9, 3.5, 1.1 Hz, 2H, C*H*_cat_), 7.09 (dd, *J* = 5.9, 3.5 Hz, 2H, C*H*_cat_).

^31^P{^1^H} NMR (243 MHz, CDCl_3_): δ 36.28.

HRMS (ESI) m/z	[M+Na^+^] calc. for C_12_H_9_O_3_PNa^+^: 255.0182;	found [M+Na^+^]: 255.0180

NMR data are in agreement with those obtained for an independently synthesised sample (see chapter 2.3).

### 5.4.2 Characterization of 2b

Work-up (non-inert): The solution was transferred into a flask, and the reaction vessel was rinsed with dichloromethane (2 mL), which was also added to the flask. All volatiles were removed at the Rotovap to obtain phosphine oxide **2b** as colorless crystals (249 mg, 97% mass balance) from 3-hydroxy-2,3-dimethylbutan-2-yl phenylphosphinate **2a** according to the general procedure from 4.4.

^1^H NMR (600 MHz, CDCl_3_): δ 7.85 – 7.79 (m, 2H, C*H*_ph_), 7.56 – 7.52 (m, 1H, C*H*_ph_), 7.48 – 7.43 (m, 2H, C*H*_ph_), 1.59 (s, 6H, C*H*_3_), 1.36 (s, 6H, C*H*_3_).

^13^C NMR (151 MHz, CDCl_3_) δ 132.45 (d, *J* = 3.3 Hz, *C*H_ph_), 131.40 (d, *J* = 10.1 Hz, *C*H_ph_), 129.18 (d, *J* = 186.3 Hz, *C*_q,ph_), 128.64 (d, *J* = 15.4 Hz, *C*H_ph_), 88.88 (*C*(CH_3_)_2_), 25.18 (d, *J* = 3.9 Hz, *C*H_3_), 24.41 (d, *J* = 5.5 Hz, *C*H_3_).

^31^P NMR (202 MHz, CDCl_3_): δ 30.35 (t, *J* = 13.9 Hz).

HRMS (ESI) m/z	[M+H^+^] calc. for C_12_H_18_O_3_P^+^: 241.0988;	found [M+H^+^]: 241.0986

## 5.5 Hydrogen measurement via GC

Procedure:

The phenylphosphinate ester **1a** or **2a** (0.2 mmol, 1 equiv.) was dissolved in dry THF (1.5 mL). A solution of dicyclohexylborane **6** (1.78 mg, 0.01 mmol, 5 mol% for **1a** or 3.56 mg, 0.02 mmol, 10 mol% for **2a**, respectively) in dry THF (0.5 mL) was added, and the reaction mixture was transferred into a GC vessel and closed with a gas-tight septum. The reaction was stirred for 6 h at 60 °C or room temperature for **1a** and **2a**, respectively. Afterwards, an aliquot of the headspace of the reaction vessel was injected into the GC. Additionally, the conversion to the phosphine oxides **1b** or **2b** was determined by ^31^P{^1^H} NMR spectroscopy using an aliquot of the reaction solution with a sealed glass capillary filled with benzene-*d*_6_.

Figure SI 31: GC spectrum from the dehydrogenation of **2a**.

Figure SI 32: GC spectrum from the dehydrogenation of **1a**.

# 6 In situ reactions

## 6.1 In situ water activation with subsequent dehydrogenation

General procedure:

2-Phenyl-1,3,2-benzodioxaphosphole **1** or 4,4,5,5-tetramethyl-2-phenyl-1,3,2-dioxaphospholane **2** (0.500 mmol, 1.00 equiv.) and dicyclohexylborane **6** (0.05 mmol, 10 mol%) were dissolved in dry THF (4 mL and 1 mL, respectively). Water (9.01 μL, 0.500 mmol, 1.00 equiv.) was added. The reaction was stirred at 1000 rpm at the specified temperature for 6 h. The conversion to the phosphine oxides **1b** or **2b** was determined by ^31^P{^1^H} NMR spectroscopy using an aliquot of the reaction solution with a sealed glass capillary filled with benzene-*d*_6_.

Scheme SI 9: General procedure for the in situ formation of phenylphosphinate esters **1a** and **2a** from water and **1** or **2**, respectively, with subsequent dehydrogenation to **1b** and **2b**.

Table SI 4: Screening results for the in situ water activation and dehydrogenation procedure.

| Entry | Starting material | *T* / °C | Conversion |
| --- | --- | --- | --- |
| 1 | **1** | 60 | 38%^[a]^ |
| 2 | **2** | 60 | 90% |
| 3 | **2** | 25 | 32% |

Conversion determined from ^31^P{^1^H} NMR. [a] Ratio of product **1b** to other observed side products; **1a** is not observed.

Figure SI 33: ^31^P{^1^H} NMR of the in situ water activation and dehydrogenation starting from 2-phenyl-1,3,2-benzodioxaphosphole **1** (202 MHz, THF). While the starting material is completely consumed and no phenylphosphinate ester **1a** is observed, formation of unidentified side products is evident, diminishing the overall yield of **1b** to 38%.

Figure SI 34: ^31^P{^1^H} NMR of the in situ water activation and dehydrogenation starting from 4,4,5,5-tetramethyl-2-phenyl-1,3,2-dioxaphospholane **2** (202 MHz, THF). The starting material is completely consumed, and the phenylphosphinate ester **2a** is converted to **2b** in 90%.

Figure SI 35: ^31^P{^1^H} NMR of the in situ water activation and dehydrogenation starting from 4,4,5,5-tetramethyl-2-phenyl-1,3,2-dioxaphospholane **2** (243 MHz, THF). The starting material is completely consumed, and the phenylphosphinate ester **2a** is converted to **2b** in 32%.

## 6.2 In situ reaction from moisture

Scheme SI 10: Procedure for the activation of moisture by 4,4,5,5-tetramethyl-2-phenyl-1,3,2-dioxaphospholane **2** and the subsequent dehydrogenation catalyzed by dicyclohexylborane **6**.

Procedure:

A solid portion of 4,4,5,5-tetramethyl-2-phenyl-1,3,2-dioxaphospholane **2** (44.8 mg, 0.2 mmol, 1 equiv.) was placed in a vial and exposed to air for 60 min. The contents of the vial were dissolved in THF (dry, 1.5 mL) and transferred into a glass tube, which was closed with a gas-tight septum. A solution of dicyclohexylborane **6** (3.56 mg, 0.02 mL, 10 mol%) in THF (dry, 0.5 mL) was added. The reaction was stirred at room temperature for 6 h. Afterwards, an aliquot of the headspace of the reaction vessel was injected into the GC. Additionally, conversion to phosphine oxide **2b** was determined by ^31^P{^1^H} NMR spectroscopy using an aliquot of the reaction solution with a sealed glass capillary filled with benzene-*d*_6_.

Figure SI 36: GC spectrum from the dehydrogenation of **2a** formed from moisture.

Figure SI 37: ^31^P NMR of the moisture activation with subsequent dehydrogenation starting from 4,4,5,5-tetramethyl-2-phenyl-1,3,2-dioxaphospholane **2** (243 MHz, THF).

# 7 Mechanistic experiments

## 7.1 Influence of trace water on the reaction progress

Stock solutions of 3-hydroxy-2,3-dimethylbutan-2-yl phenylphosphinate **2a** (157 mg in 5.2 mL, 0.125 M), dicyclohexylborane **6** (11.6 mg in 1.3 mL, 0.05 M), and water (18 µL in 1 mL, 1 M) in THF were prepared. Six glass tubes equipped with a J. Young valve were filled with the **2a** stock solution (800 µL, 0.1 mmol, 1 equiv.). To these solutions, water was added (either 0, 0.05, or 0.1 equiv., see table below). Dicyclohexylborane (200 µL of the stock solution, 0.1 equiv.) was added to every sample, and the reactions were stirred for 2 h at the denoted temperature. Afterwards, the conversion to phosphine oxide **2b** was determined by ^31^P{^1^H} NMR spectroscopy using an aliquot of the reaction solution with a sealed glass capillary filled with benzene-*d*_6_.

Table SI 5: Influence of small amounts of water on the conversion of the dehydrogenation.

| Entry | H_2_O equiv. | *T* / °C | Conversion / %^[a]^ |
| --- | --- | --- | --- |
| 1 | 0 | 25 | 74 |
| 2 | 0 | 60 | 99 |
| 3 | 0.05 | 25 | 24 |
| 4 | 0.05 | 60 | 44 |
| 5 | 0.1 | 25 | 16 |
| 6 | 0.1 | 60 | 39 |

All reactions were carried out at a 0.1 mmol scale in THF (0.1 M). [a] Conversion determined from ^31^P{^1^H} NMR as ratio of product to starting material.

## 7.2 Studies on the catalyst deactivation

Scheme SI 11: The reaction of dicyclohexylborane **6** with water in the presence of phospholane **2** yields 1,1,3,3-tetracyclohexylboroxane **7**.

Procedure:

Water (0.9 µL, 0.05 mmol, 1 equiv.) was added to an NMR tube equipped with a benzene-*d*_6_ capillary containing a solution of 4,4,5,5-tetramethyl-2-phenyl-1,3,2-dioxaphospholane **2** (11.2 mg, 0.05 mmol, 1 equiv.) and dicyclohexylborane (8.91 mg, 0.05 mmol, 1 equiv.) in THF (0.5 mL). Formation of 1,1,3,3-tetracyclohexyldiboroxane **7** with a ^11^B shift of 52.5 ppm became evident. The same reaction takes place in the absence of phospholane **2**.

Figure SI 38: 11B NMR spectrum of the reaction of dicyclohexylborane **6** with water in the presence of 4,4,5,5-tetramethyl-2-phenyl-1,3,2-dioxaphospholane **2** under the reaction conditions for the dehydrogenation of **2a** (193 MHz, THF). Quantitative formation of 1,1,3,3-tetracyclohexyldiboroxane **7** is observed.

### 7.2.1 Synthesis of 1,1,3,3-tetracyclohexyldiboroxane 7

Scheme SI 12: Synthesis of 1,1,3,3-tetracyclohexyldiboroxane **7** from dicyclohexylborane **6** and water.

Procedure:

Dicyclohexylborane (178 mg, 1.0 mmol, 1 equiv.) was dissolved in THF (10 mL). Water (10.8 µL, 0.6 mmol, 0.6 equiv.) was added to the stirred solution. After stirring at room temperature for 1 h, the solvent was removed in vacuo. The crude solid residue was crystallized from a concentrated solution of *n*-pentane at −36 °C to yield 1,1,3,3-tetracyclohexyldiboroxane **7** as colorless crystals (44 mg, 0.12 mmol, 24%).

^1^H NMR (600 MHz, C_6_D_6_) δ 1.81 – 1.69 (m, 10H, C*H*_2_), 1.35 – 1.17 (m, 10H, C*H*_2_), 1.09 (tt, *J* = 11.9, 2.9 Hz, 4H, BC*H*).

^11^B NMR (193 MHz, C_6_D_6_) δ 50.30.

Analytical data are in agreement with the literature.^[14]^

Figure SI 39: ^1^H NMR spectrum of 1,1,3,3-tetracyclohexyldiboroxane **7** (600 MHz, benzene-d_6_).

Figure SI 40: ^11^B NMR spectrum of 1,1,3,3-tetracyclohexyldiboroxane **7** (193 MHz, benzene-d_6_).

### 7.2.2 1,1,3,3-Tetracyclohexyldiboroxane 7 as catalyst for the dehydrogenation of phenylphosphinate esters 1a and 2a

Scheme SI 13: Dehydrogenation of phenylphosphinate esters **1a** and **2a** catalyzed by 1,1,3,3-tetracyclohexylboroxane **7**.

Procedure:

The phenylphosphinate ester **1a** or **2a** (0.05 mmol, 1 equiv.) was dissolved in THF (0.4 mL) and transferred into an NMR tube equipped with a J. Young valve and a benzene-*d*_6_ capillary. A solution of 1,1,3,3-tetracyclohexyldiboroxane **7** (1.85 mg, 0.005 mmol, 10 mol%) in THF (0.1 mL) was added, and the tube was sealed. The reactions were heated to 60 °C for 6 h. The dehydrogenation was checked by ^31^P NMR spectroscopy, which was complete for both substrates.

Figure SI 41: ^31^P NMR spectrum obtained from the dehydrogenation of **1a** catalyzed by 1,1,3,3-tetracyclohexylboroxane **7** (243 MHz, THF).

Figure SI 42: ^31^P NMR spectrum obtained from the dehydrogenation of **2a** catalyzed by 1,1,3,3-tetracyclohexylboroxane **7** (243 MHz, THF).

## 7.3 Identification of the resting state of the catalytic dehydrogenation

Procedure:

The phosphine oxides **1b** (11.6 mg, 0.05 mmol, 1 equiv.) or **2b** (12.0 mg, 0.05 mmol, 1 equiv.) were dissolved in THF (dry, 0.5 mL). The solution was added to a vial containing dicyclohexylborane **6** (8.91 mg, 0.05 mmol, 1 equiv.). After the initially formed suspension turned into a clear solution, it was transferred into an NMR tube equipped with a J. Young valve and a benzene-*d*_6_ capillary.

### 7.3.1 Formation of the resting state 1g

Scheme SI 14: Formation of the resting state **1g** upon a stoichiometric reaction between phosphine oxide **1b** and dicyclohexylborane **6**.

According to the procedure above, a new species in the ^31^P NMR was formed with a chemical shift of 27.89 ppm and a coupling pattern (dt, *J* = 597.0, 14.9 Hz) that clearly indicates the presence of a P–H bond. The corresponding signal in the ^11^B NMR appears at 40.06 ppm. Additionally, the presence of approximately 10% phosphine oxide **1b** is observed. Isolation of the intermediate **1g** was not feasible, and it was therefore characterized from the reaction solution.

^1^H NMR (600 MHz, THF-*d*_8_): δ 8.04 (d, *J* = 596.0 Hz, 1H, P*H*), 7.90 (ddt, *J* = 14.5, 6.8, 1.4 Hz, 2H, C*H*_ph_), 7.70 – 7.66 (m, 1H, C*H*_ph_), 7.58 (td, *J* = 7.7, 3.7 Hz, 2H, C*H*_ph_), 7.26 – 7.22 (m, 1H, C*H*_cat_), 7.09 – 7.03 (m, 1H, C*H*_cat_), 6.98 – 6.94 (m, 2H, C*H*_cat_), 1.71 – 1.60 (m, 10H, C*H*_2_), 1.31 – 1.07 (m, 10H, C*H*_2_), 0.90 (tt, *J* = 12.3, 2.8 Hz, 2H, BC*H*).

^11^B NMR (160 MHz, THF): δ 40.06.

^31^P NMR (202 MHz, THF): δ 27.89 (dt, *J* = 597.0, 14.9 Hz).

HRMS (ESI) m/z	[M−H^+^] calc. for C_24_H_31_BO_3_P^−^: 409.2103;	found [M−H^+^]: 409.2098

Furthermore, an acetonitrile adduct [M−H^+^+MeCN] and a typical fragment obtained from the cleavage of the P−O phosphinate ester bond were detected.

HRMS (ESI) m/z	[M−H^+^+MeCN] calc. for C_26_H_34_BNO_3_P^−^: 450.2366;	found [M−H^+^+MeCN]: 450.2363

HRMS (ESI) m/z	[M−PhPOH] calc. for C_18_H_26_BO_2_^−^: 285.2031;	found [M− PhPOH]: 285.2026

An unambiguous assignment of the ^13^C NMR spectra is not possible due to partial overlap with the signals of **1b**, which is present in the reaction mixture as a minor component. The unassigned ^13^C, HSQC, and HMBC NMR spectra are shown below.

Figure SI 43: ^1^H NMR of the stoichiometric reaction between phosphine oxide **1b** and dicyclohexylborane **6**, forming the resting state **1g**. Top left: Excerpt of the aromatic region. The main impurity is unreacted phosphine oxide **1b** (600 MHz, THF‑d_8_).

Figure SI 44: ^31^P{^1^H} NMR of the stoichiometric reaction between phosphine oxide **1b** and dicyclohexylborane **6**, forming the resting state **1g** in approximately 90% conversion. Top left: Excerpt of the ^31^P{^1^H} NMR showing the two peaks. Top right: Excerpt of the ^31^P NMR showing the coupling pattern of all signals (202 MHz, THF).

Figure SI 45: ^11^B NMR of the resting state **1g** formed upon the stoichiometric reaction between phosphine oxide **1b** and dicyclohexylborane **6** (160 MHz, THF).

Figure SI 46: ^13^C NMR of the resting state **1g** formed upon the stoichiometric reaction between phosphine oxide **1b** and dicyclohexylborane **6** (151 MHz, THF-d_8_).

Figure SI 47: COSY NMR of the resting state **1g** formed upon the stoichiometric reaction between phosphine oxide **1b** and dicyclohexylborane **6** (600 MHz, THF-d_8_).

Figure SI 48: HSQC NMR of the resting state **1g** formed upon the stoichiometric reaction between phosphine oxide **1b** and dicyclohexylborane **6** (151 MHz, THF-d_8_).

Figure SI 49: HMBC NMR of the resting state **1g** formed upon the stoichiometric reaction between phosphine oxide **1b** and dicyclohexylborane **6** (151 MHz, THF-d_8_).

### 7.3.2 Dehydrogenation of phenylphosphinate ester 1a catalyzed by 1g

Scheme SI 15: Dehydrogenation of 2-hydroxyphenyl phenylphosphinate **1a** catalyzed by intermediate **1g**.

Procedure:

2-Phenyl-1,3,2-benzodioxaphosphole 2-oxide **1b** (23.2 mg, 0.10 mmol, 0.2 equiv.) was dissolved in THF (dry, 1 mL). The solution was added to a vial containing dicyclohexylborane **6** (17.8 mg, 0.1 mmol, 0.2 equiv.). After the initially formed suspension turned into a clear solution, one half of it was transferred into an NMR tube equipped with a J. Young valve and a benzene-*d*_6_ capillary to confirm the formation of 1g. 2-Hydroxyphenyl phenylphosphinate **1a** (117 mg, 0.5 mmol, 1 equiv.) was dissolved in THF (4.5 mL) and transferred into a Schlenk tube equipped with a J. Young valve. To this solution was added the remaining half of the freshly prepared solution of **1g** (0.5 mL, 0.05 mmol, 10 mol%). The reaction was stirred at 60 °C for 6 h.

Figure SI 50: ^31^P{^1^H} NMR spectrum obtained from the dehydrogenation of **1a** catalyzed by **1g** (243 MHz, THF). Complete conversion of the phenylphosphinate ester **1a** is achieved, but formation of an unidentified side product is observed. However, this is not untypical for **1a** also when the standard catalyst is employed (compare, for example, Figure SI 21 or Figure SI 33).

### 7.3.3 Formation of the resting state 2f

Scheme SI 16: The stoichiometric reaction between phosphine oxide **2b** and dicyclohexylborane **6** does not lead to a hydroboration of **2b**.

When the stoichiometric reaction between phosphine oxide **2b** and dicyclohexylborane **6** was conducted according to the procedure above, no significant changes in the ^31^P NMR were obtained compared to a spectrum of isolated **2b**. The ^11^B NMR showed two main species; the one at 19.14 ppm corresponds to the THF adduct of **6** (compare Figure SI 53), whereas the one at −12.21 ppm is tentatively assigned to dicyclohexylborane **6** coordinating to the phosphine oxide. It should, however, be noted that no effects of this coordination can be seen in the ^31^P NMR.

Figure SI 51: ^31^P{^1^H} NMR of the stoichiometric reaction between phosphine oxide **2b** and dicyclohexylborane **6**. Top right: Excerpt of the ^31^P NMR showing the coupling pattern of all signals (202 MHz, THF).

Figure SI 52: ^11^B NMR of the stoichiometric reaction between phosphine oxide **2b** and dicyclohexylborane **6** (160 MHz, THF).

Figure SI 53: ^11^B NMR spectrum of dicyclohexylborane **6** in THF (193 MHz, THF).

# 8 Kinetic measurements

## 8.1 Sample preparation

A stock solution of 3-hydroxy-2,3-dimethylbutan-2-yl phenylphosphinate **2a** in THF (605 mg in 15.0 mL, 0.167 M) was prepared and stored over molecular sieves for a minimum of 24 h. A 1.5 mL aliquot of the stock solution was filled up with THF so that an overall volume of 2.5 mL (0.1 M of **2a**) is achieved after addition of the catalyst stock solution. A stock solution of dicyclohexylborane **6** in THF (17.8 mg in 5.0 mL, 0.02 M) was prepared, and aliquots thereof (e.g., 125 µL for 1 mol%) were added to the stirred solution of **2a**. The solution was stirred for 1 min to allow for complete mixing, then 0.5 mL were transferred into an NMR tube equipped with a J. Young valve and a benzene-*d*_6_ capillary.

## 8.2 NMR data acquisition and evaluation

Measurement series were recorded at a Varian Inova 500 MHz NMR spectrometer. A series of ^31^P{^1^H} NMR spectra with intervals of 5 min between individual datapoints was recorded at 25 °C. For 1, 2, and 3 mol%, two measurement series with 30 and 60 datapoints, respectively, were recorded. The relaxation delay was set to 2 s. Concentrations of **2a** and **2b** were calculated from their respective integral ratios and the set starting concentration of **2a**. The obtained concentration profiles for the decrease of 2a in all six measurement series are depicted in Figure SI 54. It should be noted that in some cases, differences in the observed reaction rates were observed, which were tentatively attributed to the presence of trace amounts of water.

Figure SI 54: Concentration profiles for the dehydrogenation with different catalyst loadings.

### 8.2.1 Influence of relaxation delay on the integration

A representative sample containing **2a** and **2b** (overall concentration 0.1 M) was prepared in THF and transferred into an NMR tube equipped with a benzene-*d*_6_ capillary. ^31^P{^1^H} NMR spectra were recorded with eight different relaxation delays ranging from 2 s (standard d_1_) to 60 s. The differences in the integral ratio were found to be small and do not significantly affect the calculated conversion (< 1%).

Figure SI 55: Excerpts of the ^31^P{^1^H} NMR spectra of a mixture of **2a** and **2b** showing the influence of the relaxation delay on the integral ratio (202 MHz, THF). The conversion that can be calculated from these integrations differs by less than 1%.

## 8.3 Variable Time Normalization Analysis (VTNA)

The obtained reaction profiles were treated according to the VTNA and plotted for different orders in catalyst. A good fit was obtained for a first-order reaction in catalyst (Figure SI 56), whereas no overlay was obtained for a fit according to a second-order reaction (Figure SI 57). Figure 2 shows only the normalised reaction profiles for the series with 60 datapoints, whereas Figure SI 56 and Figure SI 57 provide a more detailed overview, including all six measurement series.

Figure SI 56: VTNA assuming a first-order reaction in catalyst concentration.

Figure SI 57: VTNA assuming a second-order reaction in catalyst concentration.

# 9 NMR spectra of isolated compounds

## 9.1 NMR spectra of dioxaphospholes and phospholanes

### 9.1.1 NMR spectra of 2-phenyl-1,3,2-benzodioxaphosphole 1

Figure SI 58: ^1^H NMR spectrum of 2-phenyl-1,3,2-benzodioxaphosphole **1** (400 MHz, chloroform-d_1_).

Figure SI 59: ^31^P{^1^H} NMR spectrum of 2-phenyl-1,3,2-benzodioxaphosphole **1** (162 MHz, chloroform-d_1_).

### 9.1.2 NMR spectra of 4,4,5,5-tetramethyl-2-phenyl-1,3,2-dioxaphospholane 2

Figure SI 60: ^1^H NMR spectrum of 4,4,5,5-tetramethyl-2-phenyl-1,3,2-dioxaphospholane **2** (400 MHz, benzene-d_6_).

Figure SI 61: ^13^C NMR spectrum of 4,4,5,5-tetramethyl-2-phenyl-1,3,2-dioxaphospholane **2** (101 MHz, benzene-d_6_).

Figure SI 62: ^31^P NMR spectrum of 4,4,5,5-tetramethyl-2-phenyl-1,3,2-dioxaphospholane **2** (162 MHz, benzene-d_6_).

Figure SI 63: COSY NMR spectrum of 4,4,5,5-tetramethyl-2-phenyl-1,3,2-dioxaphospholane **2** (400 MHz, benzene-d_6_).

Figure SI 64: HSQC NMR spectrum of 4,4,5,5-tetramethyl-2-phenyl-1,3,2-dioxaphospholane **2** (101 MHz, benzene-d_6_).

Figure SI 65: HMBC NMR spectrum of 4,4,5,5-tetramethyl-2-phenyl-1,3,2-dioxaphospholane **2** (101 MHz, benzene-d_6_).

## 9.2 NMR spectra of phenylphosphinate esters

### 9.2.1 NMR spectra of 2-hydroxyphenyl phenylphosphinate 1a

Figure SI 66: ^1^H NMR spectrum of 2-hydroxyphenyl phenylphosphinate **1a** (400 MHz, chloroform-d_1_).

Figure SI 67: ^13^C{^1^H} NMR spectrum of 2-hydroxyphenyl phenylphosphinate **1a** (101 MHz, chloroform-d_1_).

Figure SI 68: ^31^P NMR spectrum of 2-hydroxyphenyl phenylphosphinate **1a** (162 MHz, chloroform-d_1_).

Figure SI 69: COSY NMR spectrum of 2-hydroxyphenyl phenylphosphinate **1a** (400 MHz, chloroform-d_1_).

Figure SI 70: HSQC NMR spectrum of 2-hydroxyphenyl phenylphosphinate **1a** (101 MHz, chloroform-d_1_).

Figure SI 71: HMBC NMR spectrum of 2-hydroxyphenyl phenylphosphinate **1a** (101 MHz, chloroform-d_1_).

### 9.2.2 NMR spectra of deuterated 2-hydroxyphenyl phenylphosphinate 1a-*d*_2_

Figure SI 72: ^1^H NMR spectrum of deuterated 2-hydroxyphenyl phenylphosphinate **1a**-**d_2_** (400 MHz, chloroform-d_1_).

Figure SI 73: ^2^H NMR spectrum of deuterated 2-hydroxyphenyl phenylphosphinate **1a**-**d_2_** (61 MHz, acetonitrile-d_3_).

Figure SI 74: ^13^C{^1^H} NMR spectrum of deuterated 2-hydroxyphenyl phenylphosphinate **1a**-**d_2_** (101 MHz, chloroform-d_1_).

Figure SI 75: ^31^P{^1^H} and ^31^P (excerpt top right) NMR spectrum of deuterated 2-hydroxyphenyl phenylphosphinate **1a**-**d_2_** (162 MHz, chloroform-d_1_). Note that small amounts of **1a** are formed in solution.

Figure SI 76: COSY NMR spectrum of deuterated 2-hydroxyphenyl phenylphosphinate **1a**-**d_2_** (400 MHz, chloroform-d_1_).

Figure SI 77: HSQC NMR spectrum of deuterated 2-hydroxyphenyl phenylphosphinate **1a**-**d_2_** (101 MHz, chloroform-d_1_).

Figure SI 78: HMBC NMR spectrum of deuterated 2-hydroxyphenyl phenylphosphinate **1a**-**d_2_** (101 MHz, chloroform-d_1_).

### 9.2.3 NMR spectra of 3-hydroxy-2,3-dimethylbutan-2-yl phenylphosphinate 2a

Figure SI 79: ^1^H NMR spectrum of 3-hydroxy-2,3-dimethylbutan-2-yl phenylphosphinate **2a** (400 MHz, chloroform-d_1_).

Figure SI 80: ^13^C NMR spectrum of 3-hydroxy-2,3-dimethylbutan-2-yl phenylphosphinate **2a** (101 MHz, chloroform-d_1_).

Figure SI 81: ^31^P NMR spectrum of 3-hydroxy-2,3-dimethylbutan-2-yl phenylphosphinate **2a** (162 MHz, chloroform-d_1_).

Figure SI 82: COSY NMR spectrum of 3-hydroxy-2,3-dimethylbutan-2-yl phenylphosphinate **2a** (400 MHz, chloroform-d_1_).

Figure SI 83: HSQC NMR spectrum of 3-hydroxy-2,3-dimethylbutan-2-yl phenylphosphinate **2a** (101 MHz, chloroform-d_1_).

Figure SI 84: HMBC NMR spectrum of 3-hydroxy-2,3-dimethylbutan-2-yl phenylphosphinate **2a** (101 MHz, chloroform-d_1_).

### 9.2.4 NMR spectra of 3-hydroxy-2,3-dimethylbutan-2-yl phenylphosphinate 2a-*d*_2_

Figure SI 85: ^1^H NMR spectrum of deuterated 3-hydroxy-2,3-dimethylbutan-2-yl phenylphosphinate **2a**-**d_2_** (400 MHz, chloroform-d_1_).

Figure SI 86: ^2^H NMR spectrum of deuterated 3-hydroxy-2,3-dimethylbutan-2-yl phenylphosphinate **2a**-**d_2_** (61 MHz, acetonitrile-d_3_).

Figure SI 87: ^13^C NMR spectrum of deuterated 3-hydroxy-2,3-dimethylbutan-2-yl phenylphosphinate **2a**-**d_2_** (101 MHz, chloroform-d_1_).

Figure SI 88: ^31^P NMR spectrum of deuterated 3-hydroxy-2,3-dimethylbutan-2-yl phenylphosphinate **2a**-**d_2_** (162 MHz, chloroform-d_1_). Note that small amounts of **2a** are formed in solution.

Figure SI 89: COSY NMR spectrum of deuterated 3-hydroxy-2,3-dimethylbutan-2-yl phenylphosphinate **2a**-**d_2_** (400 MHz, chloroform-d_1_).

Figure SI 90: HSQC NMR spectrum of deuterated 3-hydroxy-2,3-dimethylbutan-2-yl phenylphosphinate **2a**-**d_2_** (101 MHz, chloroform-d_1_).

Figure SI 91: ^1^H NMR spectrum of deuterated 3-hydroxy-2,3-dimethylbutan-2-yl phenylphosphinate **2a**-**d_2_** (101 MHz, chloroform-d_1_).

## 9.3 NMR spectra of phosphine oxides

### 9.3.1 NMR spectra of 1b obtained from preparative dehydrogenation

Figure SI 92: ^1^H NMR spectrum of 2-phenyl-1,3,2-benzodioxaphospholane 2-oxide **1b** as obtained from the dehydrogenation of **1a** (600 MHz, chloroform‑d_1_).

Figure SI 93: ^31^P{^1^H} NMR spectrum of 2-phenyl-1,3,2-benzodioxaphospholane 2-oxide **1b** as obtained from the dehydrogenation of **1a** (243 MHz, chloroform‑d_1_).

### 9.3.2 NMR spectra of 2b obtained from preparative dehydrogenation

Figure SI 94: ^1^H NMR spectrum of 4,4,5,5-tetramethyl-2-phenyl-1,3,2-dioxaphospholane 2-oxide **2b** as obtained from the dehydrogenation of **2a** (600 MHz, chloroform‑d_1_).

Figure SI 95: ^13^C NMR spectrum of 4,4,5,5-tetramethyl-2-phenyl-1,3,2-dioxaphospholane 2-oxide **2b** as obtained from the dehydrogenation of **2a** (151 MHz, chloroform‑d_1_).

Figure SI 96: ^31^P NMR spectrum of 4,4,5,5-tetramethyl-2-phenyl-1,3,2-dioxaphospholane 2-oxide **2b** as obtained from the dehydrogenation of **2a** (202 MHz, chloroform‑d_1_).

Figure SI 97: COSY NMR spectrum of 4,4,5,5-tetramethyl-2-phenyl-1,3,2-dioxaphospholane 2-oxide **2b** as obtained from the dehydrogenation of **2a** (600 MHz, chloroform‑d_1_).

Figure SI 98: HSQC NMR spectrum of 4,4,5,5-tetramethyl-2-phenyl-1,3,2-dioxaphospholane 2-oxide **2b** as obtained from the dehydrogenation of **2a** (151 MHz, chloroform‑d_1_).

Figure SI 99: HMBC NMR spectrum of 4,4,5,5-tetramethyl-2-phenyl-1,3,2-dioxaphospholane 2-oxide **2b** as obtained from the dehydrogenation of **2a** (151 MHz, chloroform‑d_1_).

### 9.3.3 NMR spectra of independently synthesised 1b

Figure SI 100: ^1^H NMR spectrum of 2-phenyl-1,3,2-benzodioxaphosphole 2-oxide **1b** (600 MHz, chloroform‑d_1_).

Figure SI 101: ^13^C NMR spectrum of 2-phenyl-1,3,2-benzodioxaphosphole 2-oxide **1b** (151 MHz, chloroform‑d_1_).

Figure SI 102: ^31^P{^1^H} NMR spectrum of 2-phenyl-1,3,2-benzodioxaphosphole 2-oxide **1b**. The excerpt shows the coupling pattern in the ^31^P NMR spectrum (243 MHz, chloroform‑d_1_).

Figure SI 103: COSY NMR spectrum of 2-phenyl-1,3,2-benzodioxaphosphole 2-oxide **1b** (600 MHz, chloroform‑d_1_).

Figure SI 104: HSQC NMR spectrum of 2-phenyl-1,3,2-benzodioxaphosphole 2-oxide **1b** (151 MHz, chloroform‑d_1_).

Figure SI 105: HMBC NMR spectrum of 2-phenyl-1,3,2-benzodioxaphosphole 2-oxide **1b** (151 MHz, chloroform‑d_1_).

## 9.4 NMR spectra of pyridonate borane 4

Figure SI 106: ^1^H NMR spectrum of the pyridonate borane **4** (400 MHz, benzene-d_6_).

Figure SI 107: ^11^B NMR spectrum of the pyridonate borane **4** (128 MHz, benzene-d_6_).

Figure SI 108: ^13^C{^1^H} NMR spectrum of the pyridonate borane **4** (101 MHz, benzene-d_6_).

Figure SI 109: COSY NMR spectrum of the pyridonate borane **4** (400 MHz, benzene-d_6_).

Figure SI 110: HSQC NMR spectrum of the pyridonate borane **4** (101 MHz, benzene-d_6_).

Figure SI 111: HMBC NMR spectrum of the pyridonate borane **4** (101 MHz, benzene-d_6_).

# 10 X-ray analysis

The crystallographic data has been deposited with the Cambridge Crystallographic Data Centre as CCDC No. 2553565 and 2553646 and can be obtained free of charge.

## 10.1 Crystallographic data of 2-hydroxyphenyl phenylphosphinate 1a

Diffraction data were collected at low temperatures (100 K) using φ- and ω-scans on a BRUKER D8 Venture system. MO-Kα radiation wavelength 0.71073 Å was used. Refinement was performed using SHELXL2018/3.^[15]^ All atoms except hydrogen were refined anisotropically, and hydrogen atoms bound to carbon or oxygen were positioned at geometrically calculated positions and refined using a rigid model.^[16,17]^ The isotropic displacement parameters of all hydrogen atoms were fixed to 1.2x using the U(eq) value of the atoms they are linked to.

The crystal structure of **1a** was solved in the monoclinic space group *P*2_1_/*c*. The asymmetric unit contains two molecules of **1a**.

Table SI 6: Crystal data and structure refinement for 2-hydroxyphenyl phenylphosphinate **1a**.

Identification code	DC22118_a

Empirical formula	C_24_H_22_O_6_P_2_

Formula weight	468.35

Temperature/K	100(2)

Crystal system	monoclinic

Space group	*P*2_1_/c

a/Å	6.8018(3)

b/Å	10.8750(4)

c/Å	30.4858(12)

α/°	90

β/°	93.099(2)

γ/°	90

Volume/Å^3^	2251.72(16)

Z	4

ρ_calc_g/cm^3^	1.382

μ/mm^−1^	0.232

F(000)	976.0

Crystal size/mm^3^	0.436 × 0.132 × 0.084

Radiation	MoKα (λ = 0.71073)

2Θ range for data collection/°	5.352 to 59.144

Index ranges	-9 ≤ h ≤ 9, -15 ≤ k ≤ 15, -42 ≤ l ≤ 42

Reflections collected	116064

Independent reflections	6306 [R_int_ = 0.0468, R_sigma_ = 0.0186]

Data/restraints/parameters	6306/2/301

Goodness-of-fit on F^2^	1.016

Final R indexes [I>=2σ (I)]	R_1_ = 0.0321, wR_2_ = 0.0996

Final R indexes [all data]	R_1_ = 0.0354, wR_2_ = 0.1034

Largest diff. peak/hole / e Å^−3^	0.44/-0.23

Table SI 7: Fractional Atomic Coordinates (×10^4^) and Equivalent Isotropic Displacement Parameters (Å^2^×10^3^) for 2‑hydroxyphenyl phenylphosphinate **1a**. U_eq_ is defined as 1/3 of the trace of the orthogonalised U_IJ_ tensor.

| **Atom** | ***x*** | ***y*** | ***z*** | **U(eq)** |
| --- | --- | --- | --- | --- |
| P1 | 3510.6(4) | 3474.5(2) | 5348.0(2) | 15.64(8) |
| O1 | 4417.6(11) | 2633.1(7) | 5737.2(2) | 18.66(15) |
| C1 | 3388.5(15) | 4931.8(9) | 5607.6(3) | 18.67(19) |
| P2 | 9244.6(4) | 10483.4(2) | 6593.3(2) | 16.16(8) |
| O2 | 1595.0(11) | 3060.3(7) | 5144.1(3) | 20.98(16) |
| C2 | 4991.0(17) | 5380.5(10) | 5868.1(4) | 22.8(2) |
| O3 | 8134.4(11) | 2986.0(7) | 5508.5(3) | 20.18(15) |
| C3 | 4817(2) | 6498.2(11) | 6081.9(4) | 29.1(2) |
| O4 | 8580.2(11) | 10111.9(7) | 7071.4(2) | 19.19(15) |
| C4 | 3080(2) | 7169.2(11) | 6033.1(4) | 31.5(3) |
| O5 | 11132.1(11) | 9920.4(8) | 6474.8(3) | 23.01(16) |
| C5 | 1508(2) | 6733.8(11) | 5770.6(4) | 30.5(3) |
| O6 | 4729.8(11) | 10170.2(7) | 6820.2(3) | 21.81(16) |
| C6 | 1654.1(17) | 5611.5(10) | 5557.6(4) | 24.1(2) |
| C7 | 5547.4(14) | 1605.8(9) | 5628.5(3) | 16.32(18) |
| C8 | 7484.2(14) | 1807.8(9) | 5516.5(3) | 16.16(18) |
| C9 | 8630.5(15) | 791.5(10) | 5419.6(3) | 19.42(19) |
| C10 | 7823.8(17) | -383.6(10) | 5428.3(3) | 21.8(2) |
| C11 | 5892.6(17) | -562.7(10) | 5540.0(3) | 22.5(2) |
| C12 | 4743.5(15) | 440.6(10) | 5645.1(3) | 20.1(2) |
| C13 | 9305.3(15) | 12118.0(10) | 6627.4(3) | 18.54(19) |
| C14 | 11007.9(17) | 12754.4(11) | 6530.0(4) | 23.1(2) |
| C15 | 11062.5(19) | 14026.5(11) | 6572.6(4) | 28.7(2) |
| C16 | 9431(2) | 14655.6(11) | 6706.8(4) | 30.5(3) |
| C17 | 7725.2(19) | 14026.6(11) | 6799.9(4) | 28.2(2) |
| C18 | 7650.1(16) | 12754.4(10) | 6759.8(4) | 22.4(2) |
| C19 | 7435.1(15) | 9055.1(9) | 7125.1(3) | 17.65(18) |
| C20 | 8279.9(16) | 8014.5(10) | 7317.2(3) | 21.1(2) |
| C21 | 7100.5(18) | 6998.3(10) | 7393.0(4) | 24.3(2) |
| C22 | 5094.0(18) | 7045.4(10) | 7281.2(4) | 24.4(2) |
| C23 | 4258.1(16) | 8097.5(10) | 7090.7(3) | 21.8(2) |
| C24 | 5421.5(15) | 9113.4(10) | 7006.8(3) | 17.92(19) |

Table SI 8: Anisotropic Displacement Parameters (Å^2^×10^3^) for 2-hydroxyphenyl phenylphosphinate **1a**. The Anisotropic displacement factor exponent takes the form: -2π^2^[h^2^a*^2^U_11_+2hka*b*U_12_+…].

| **Atom** | **U_11_** | **U_22_** | **U_33_** | **U_23_** | **U_13_** | **U_12_** |
| --- | --- | --- | --- | --- | --- | --- |
| P1 | 12.26(12) | 17.12(13) | 17.56(13) | 2.61(8) | 0.89(9) | 0.51(8) |
| O1 | 16.6(3) | 20.4(3) | 19.3(3) | 3.7(3) | 4.0(3) | 5.5(3) |
| C1 | 20.7(5) | 17.2(4) | 17.9(4) | 2.9(3) | -0.3(4) | -0.6(3) |
| P2 | 12.55(12) | 19.61(14) | 16.17(13) | 0.15(8) | -0.50(9) | 1.07(8) |
| O2 | 14.8(3) | 21.2(4) | 26.6(4) | -0.6(3) | -1.3(3) | -0.5(3) |
| C2 | 23.3(5) | 22.9(5) | 21.9(5) | 2.4(4) | -1.2(4) | -3.9(4) |
| O3 | 15.8(3) | 17.1(3) | 27.9(4) | -0.2(3) | 3.3(3) | -1.0(3) |
| C3 | 36.9(6) | 24.8(5) | 25.1(5) | -0.7(4) | -2.1(5) | -7.8(5) |
| O4 | 18.9(3) | 21.5(4) | 16.9(3) | 1.7(3) | -1.3(3) | -3.5(3) |
| C4 | 50.9(8) | 18.0(5) | 25.6(5) | 0.1(4) | 0.3(5) | 1.1(5) |
| O5 | 14.8(3) | 26.3(4) | 27.8(4) | -1.7(3) | 1.2(3) | 4.1(3) |
| C5 | 41.3(7) | 21.6(5) | 28.1(6) | 1.9(4) | -1.6(5) | 11.2(5) |
| O6 | 16.2(3) | 20.9(4) | 28.0(4) | 0.7(3) | -1.7(3) | 3.3(3) |
| C6 | 27.5(5) | 21.4(5) | 23.0(5) | 1.8(4) | -3.3(4) | 5.2(4) |
| C7 | 15.0(4) | 17.8(4) | 16.1(4) | 1.4(3) | 0.1(3) | 2.8(3) |
| C8 | 15.9(4) | 16.4(4) | 16.1(4) | 0.7(3) | 0.2(3) | 0.7(3) |
| C9 | 18.3(4) | 21.3(5) | 18.8(4) | 0.0(4) | 3.0(3) | 4.3(4) |
| C10 | 29.9(5) | 17.5(5) | 18.1(5) | -0.5(3) | 2.5(4) | 5.2(4) |
| C11 | 30.3(5) | 17.3(5) | 19.7(5) | 1.0(4) | -2.0(4) | -2.8(4) |
| C12 | 18.2(4) | 22.7(5) | 19.1(5) | 3.3(4) | -1.5(4) | -2.7(4) |
| C13 | 19.2(5) | 20.3(5) | 16.0(4) | 1.5(3) | -0.1(3) | 0.7(3) |
| C14 | 22.9(5) | 26.4(5) | 20.3(5) | 1.8(4) | 4.3(4) | -2.4(4) |
| C15 | 35.4(6) | 26.3(6) | 25.0(5) | 4.2(4) | 5.7(4) | -8.1(5) |
| C16 | 43.0(7) | 21.5(5) | 27.1(6) | 3.6(4) | 2.3(5) | -1.1(5) |
| C17 | 31.9(6) | 22.7(5) | 29.8(6) | 0.9(4) | 1.2(4) | 6.7(4) |
| C18 | 21.1(5) | 23.1(5) | 22.9(5) | 0.8(4) | 0.2(4) | 2.6(4) |
| C19 | 17.6(4) | 19.7(5) | 15.6(4) | -0.4(3) | 0.5(3) | -1.8(4) |
| C20 | 19.8(5) | 24.6(5) | 18.6(4) | 2.2(4) | -2.0(4) | 1.6(4) |
| C21 | 29.4(6) | 22.3(5) | 20.9(5) | 4.2(4) | -0.4(4) | 0.5(4) |
| C22 | 28.2(5) | 24.0(5) | 21.0(5) | 1.8(4) | 2.5(4) | -5.5(4) |
| C23 | 18.0(5) | 26.7(5) | 20.7(5) | -1.1(4) | 2.3(4) | -2.4(4) |
| C24 | 17.8(4) | 19.9(5) | 16.1(4) | -2.3(3) | 1.7(3) | 1.8(3) |

Table SI 9: Bond Lengths for 2-hydroxyphenyl phenylphosphinate **1a**.

| **Atom** | **Atom** | **Length/Å** |  | **Atom** | **Atom** | **Length/Å** |
| --- | --- | --- | --- | --- | --- | --- |
| P1 | O1 | 1.5958(8) |  | C7 | C12 | 1.3821(14) |
| P1 | C1 | 1.7753(11) |  | C8 | C9 | 1.3934(13) |
| P1 | O2 | 1.4831(8) |  | C9 | C10 | 1.3916(15) |
| O1 | C7 | 1.4056(12) |  | C10 | C11 | 1.3883(16) |
| C1 | C2 | 1.4012(14) |  | C11 | C12 | 1.3897(15) |
| C1 | C6 | 1.3935(15) |  | C13 | C14 | 1.3948(15) |
| P2 | O4 | 1.6009(8) |  | C13 | C18 | 1.3994(14) |
| P2 | O5 | 1.4845(8) |  | C14 | C15 | 1.3898(17) |
| P2 | C13 | 1.7810(11) |  | C15 | C16 | 1.3841(19) |
| C2 | C3 | 1.3872(16) |  | C16 | C17 | 1.3895(18) |
| O3 | C8 | 1.3561(12) |  | C17 | C18 | 1.3896(16) |
| C3 | C4 | 1.3897(19) |  | C19 | C20 | 1.3847(14) |
| O4 | C19 | 1.4030(12) |  | C19 | C24 | 1.3988(14) |
| C4 | C5 | 1.3842(19) |  | C20 | C21 | 1.3922(16) |
| C5 | C6 | 1.3886(16) |  | C21 | C22 | 1.3897(16) |
| O6 | C24 | 1.3552(12) |  | C22 | C23 | 1.3906(16) |
| C7 | C8 | 1.3961(14) |  | C23 | C24 | 1.3907(15) |

Table SI 10: Bond Angles for 2-hydroxyphenyl phenylphosphinate **1a**.

| **Atom** | **Atom** | **Atom** | **Angle/˚** |  | **Atom** | **Atom** | **Atom** | **Angle/˚** |
| --- | --- | --- | --- | --- | --- | --- | --- | --- |
| O1 | P1 | C1 | 101.88(4) |  | C10 | C9 | C8 | 119.91(10) |
| O2 | P1 | O1 | 115.66(4) |  | C11 | C10 | C9 | 120.85(10) |
| O2 | P1 | C1 | 113.38(5) |  | C10 | C11 | C12 | 119.85(10) |
| C7 | O1 | P1 | 118.35(6) |  | C7 | C12 | C11 | 118.87(10) |
| C2 | C1 | P1 | 120.65(8) |  | C14 | C13 | P2 | 119.98(8) |
| C6 | C1 | P1 | 118.98(8) |  | C14 | C13 | C18 | 120.41(10) |
| C6 | C1 | C2 | 120.35(10) |  | C18 | C13 | P2 | 119.61(8) |
| O4 | P2 | C13 | 101.85(4) |  | C15 | C14 | C13 | 119.49(11) |
| O5 | P2 | O4 | 114.07(5) |  | C16 | C15 | C14 | 120.13(11) |
| O5 | P2 | C13 | 114.10(5) |  | C15 | C16 | C17 | 120.58(11) |
| C3 | C2 | C1 | 119.19(11) |  | C16 | C17 | C18 | 119.95(11) |
| C2 | C3 | C4 | 120.25(11) |  | C17 | C18 | C13 | 119.44(11) |
| C19 | O4 | P2 | 119.93(6) |  | C20 | C19 | O4 | 119.91(9) |
| C5 | C4 | C3 | 120.52(11) |  | C20 | C19 | C24 | 121.62(10) |
| C4 | C5 | C6 | 119.89(11) |  | C24 | C19 | O4 | 118.34(9) |
| C5 | C6 | C1 | 119.79(11) |  | C19 | C20 | C21 | 119.29(10) |
| C8 | C7 | O1 | 117.89(9) |  | C22 | C21 | C20 | 119.80(10) |
| C12 | C7 | O1 | 119.85(9) |  | C23 | C22 | C21 | 120.45(10) |
| C12 | C7 | C8 | 122.25(9) |  | C22 | C23 | C24 | 120.43(10) |
| O3 | C8 | C7 | 117.78(9) |  | O6 | C24 | C19 | 117.36(9) |
| O3 | C8 | C9 | 123.97(9) |  | O6 | C24 | C23 | 124.25(9) |
| C9 | C8 | C7 | 118.25(9) |  | C23 | C24 | C19 | 118.39(9) |

Table SI 11: Hydrogen Bonds for 2-hydroxyphenyl phenylphosphinate **1a**.

| **D** | **H** | **A** | **d(D-H)/Å** | **d(H-A)/Å** | **d(D-A)/Å** | **D-H-A/°** |
| --- | --- | --- | --- | --- | --- | --- |
| O3 | H3A | O2^1^ | 0.85 | 1.81 | 2.6578(11) | 173.9 |
| O6 | H6A | O5^2^ | 0.82 | 1.81 | 2.6253(11) | 173.6 |

^1^1+X,+Y,+Z; ^2^-1+X,+Y,+Z

Table SI 12: Hydrogen Atom Coordinates (Å×10^4^) and Isotropic Displacement Parameters (Å^2^×10^3^) for 2-hydroxyphenyl phenylphosphinate **1a**.

| **Atom** | ***x*** | ***y*** | ***z*** | **U(eq)** |
| --- | --- | --- | --- | --- |
| H1A | 4830(20) | 3475(12) | 5063(4) | 17(3) |
| H2A | 7770(20) | 10110(13) | 6343(5) | 23(3) |
| H2Y | 6182.41 | 4924.86 | 5898.02 | 27 |
| H3A | 9280(20) | 3003.1(17) | 5411(5) | 30 |
| H3 | 5888.43 | 6805.97 | 6262.2 | 35 |
| H4 | 2970.69 | 7932.81 | 6181.23 | 38 |
| H5Y | 330.37 | 7202.07 | 5736.11 | 37 |
| H6A | 3580(20) | 10073(5) | 6731(5) | 33 |
| H6 | 575.36 | 5307.96 | 5378.39 | 29 |
| H9 | 9960.88 | 900.73 | 5347.72 | 23 |
| H10 | 8604.64 | -1072.12 | 5357 | 26 |
| H11 | 5357.93 | -1369.33 | 5544.44 | 27 |
| H12 | 3427.73 | 327.48 | 5726.97 | 24 |
| H14 | 12121.69 | 12321.57 | 6435.25 | 28 |
| H15 | 12221.62 | 14465.03 | 6509.44 | 34 |
| H16 | 9478.62 | 15525.13 | 6735.57 | 37 |
| H17 | 6610.89 | 14465.99 | 6890.98 | 34 |
| H18 | 6485.05 | 12320.42 | 6821.65 | 27 |
| H20 | 9649.66 | 7994.16 | 7396.38 | 25 |
| H21 | 7665.71 | 6274.85 | 7520.89 | 29 |
| H22 | 4287.94 | 6354.19 | 7335.17 | 29 |
| H23 | 2883.36 | 8122.35 | 7017.33 | 26 |

## 10.2 Crystallographic data of 3-hydroxy-2,3-dimethylbutan-2-yl phenylphosphinate 2a

Single crystals of C_12_H_19_O_3_P **2a** were grown from a concentrated solution of **2a** in dichloromethane, which was overlaid with *n*-hexane and stored at −36 °C. A suitable crystal was selected and measured on a Bruker Kappa APEXII Duo diffractometer.^[18,19]^ The crystal was kept at 100.01 K during data collection. Using Olex2,^[20]^ the structure was solved with the SHELXT^[21]^ structure solution program using Intrinsic Phasing and refined with the SHELXL^[15]^ refinement package using Least Squares minimisation.

Crystal Data for C_12_H_19_O_3_P (*M* =242.24 g/mol): monoclinic, space group *P*2_1_/n (no. 14), *a* = 6.6901(5) Å, *b* = 25.4488(17) Å, *c* = 7.9051(6) Å, *β* = 111.401(3)°, *V* = 1253.08(16) Å^3^, *Z* = 4, *T* = 100.01 K, μ(MoKα) = 0.210 mm^-1^, *D_calc_* = 1.284 g/cm^3^, 5870 reflections measured (3.2° ≤ 2Θ ≤ 57.584°), 3086 unique (*R*_int_ = 0.0293, R_sigma_ = 0.0480) which were used in all calculations. The final *R*_1_ was 0.0448 (I > 2σ(I)), and *wR*_2_ was 0.1129 (all data).

Table SI 13: Crystal data and structure refinement for 3-hydroxy-2,3-dimethylbutan-2-yl phenylphosphinate **2a**.

Identification code	mo_ge001_nk1_4

Empirical formula	C12H19O3P

Formula weight	242.24

Temperature/K	100.01

Crystal system	monoclinic

Space group	*P*2_1_/n

a/Å	6.6901(5)

b/Å	25.4488(17)

c/Å	7.9051(6)

α/°	90

β/°	111.401(3)

γ/°	90

Volume/Å^3^	1253.08(16)

Z	4

ρ_calc_g/cm^3^	1.284

μ/mm^−1^	0.210

F(000)	520.0

Crystal size/mm^3^	0.247 × 0.164 × 0.151

Radiation	MoKα (λ = 0.71073)

2Θ range for data collection/°	3.2 to 57.584

Index ranges	-8 ≤ h ≤ 8, -33 ≤ k ≤ 33, 0 ≤ l ≤ 10

Reflections collected	5870

Independent reflections	3086 [R_int_ = 0.0293, R_sigma_ = 0.0480]

Data/restraints/parameters	3086/0/157

Goodness-of-fit on F^2^	1.124

Final R indexes [I>=2σ (I)]	R_1_ = 0.0448, wR_2_ = 0.1098

Final R indexes [all data]	R_1_ = 0.0566, wR_2_ = 0.1129

Largest diff. peak/hole / e Å^−3^	0.29/-0.46

Table SI 14: Fractional Atomic Coordinates (×10^4^) and Equivalent Isotropic Displacement Parameters (Å^2^×10^3^) for 3-hydroxy-2,3-dimethylbutan-2-yl phenylphosphinate **2a**. U_eq_ is defined as 1/3 of the trace of the orthogonalised U_IJ_ tensor.

| **Atom** | ***x*** | ***y*** | ***z*** | **U(eq)** |
| --- | --- | --- | --- | --- |
| P1 | 5322.9(7) | 3571.1(2) | 5243.4(5) | 14.27(13) |
| O1 | 7445(2) | 3472.0(5) | 5154.3(16) | 22.7(3) |
| O2 | 5291(2) | 3690.7(4) | 7185.9(15) | 17.1(3) |
| O3 | 1598(2) | 3130.0(5) | 6613.7(17) | 22.9(3) |
| C1 | 5153(3) | 3309.8(6) | 8552(2) | 14.5(3) |
| C2 | 6721(3) | 3527.5(7) | 10342(2) | 20.8(4) |
| C3 | 5865(3) | 2764.9(6) | 8189(2) | 18.4(3) |
| C4 | 2799(3) | 3315.6(6) | 8408(2) | 15.7(3) |
| C5 | 2018(3) | 3865.5(6) | 8644(2) | 19.8(3) |
| C6 | 2454(3) | 2942.8(7) | 9796(3) | 26.8(4) |
| C7 | 4057(3) | 4141.9(6) | 3985(2) | 14.4(3) |
| C8 | 5287(3) | 4486.5(6) | 3395(2) | 16.6(3) |
| C9 | 4359(3) | 4938.7(6) | 2449(2) | 21.6(4) |
| C10 | 2225(3) | 5043.1(7) | 2096(2) | 23.7(4) |
| C11 | 992(3) | 4700.4(7) | 2668(2) | 24.9(4) |
| C12 | 1910(3) | 4246.7(7) | 3621(2) | 20.1(4) |

Table SI 15: Anisotropic Displacement Parameters (Å^2^×10^3^) for 3-hydroxy-2,3-dimethylbutan-2-yl phenylphosphinate **2a**. The Anisotropic displacement factor exponent takes the form: -2π^2^[h^2^a*^2^U_11_+2hka*b*U_12_+…].

| **Atom** | **U_11_** | **U_22_** | **U_33_** | **U_23_** | **U_13_** | **U_12_** |
| --- | --- | --- | --- | --- | --- | --- |
| P1 | 17.1(3) | 14.1(2) | 11.7(2) | 0.19(13) | 5.48(15) | 1.82(14) |
| O1 | 19.7(7) | 29.8(7) | 20.2(6) | 4.5(5) | 9.2(5) | 8.5(5) |
| O2 | 27.2(7) | 13.0(5) | 12.7(5) | -0.4(4) | 9.4(5) | -1.2(4) |
| O3 | 15.4(7) | 25.0(6) | 23.5(6) | -10.7(5) | 1.3(5) | 2.9(5) |
| C1 | 19.2(9) | 13.6(7) | 11.6(7) | 1.7(5) | 6.8(6) | 1.1(5) |
| C2 | 19.0(10) | 27.9(9) | 14.3(8) | -1.2(6) | 4.5(6) | -0.6(6) |
| C3 | 20.9(10) | 16.2(7) | 20.1(8) | 3.1(6) | 10.0(7) | 5.2(6) |
| C4 | 16.9(9) | 14.2(7) | 16.4(8) | -1.6(5) | 6.4(6) | 0.8(5) |
| C5 | 21.2(10) | 17.6(8) | 21.2(8) | -4.2(6) | 8.5(7) | 2.6(6) |
| C6 | 26.8(11) | 24.5(9) | 35.2(10) | 7.1(7) | 18.6(8) | 0.9(7) |
| C7 | 18.0(9) | 13.8(7) | 10.5(7) | -0.6(5) | 4.2(6) | 0.2(6) |
| C8 | 16.9(9) | 18.3(7) | 15.8(7) | -2.1(6) | 7.4(6) | -1.9(6) |
| C9 | 32.6(11) | 15.7(8) | 17.4(8) | -1.6(6) | 10.1(7) | -5.1(6) |
| C10 | 31.4(11) | 17.6(8) | 18.9(8) | 2.6(6) | 5.4(7) | 5.1(6) |
| C11 | 20.5(10) | 27.4(9) | 24.4(9) | 3.8(7) | 5.5(7) | 7.0(7) |
| C12 | 18.3(10) | 22.0(8) | 20.0(8) | 3.0(6) | 7.0(7) | 0.9(6) |

Table SI 16: Bond Lengths for 3-hydroxy-2,3-dimethylbutan-2-yl phenylphosphinate **2a**.

| P1 | O1 | 1.4687(14) |  | C4 | C5 | 1.529(2) |
| --- | --- | --- | --- | --- | --- | --- |
| P1 | O2 | 1.5732(11) |  | C4 | C6 | 1.530(2) |
| P1 | C7 | 1.7895(16) |  | C7 | C8 | 1.395(2) |
| O2 | C1 | 1.4790(18) |  | C7 | C12 | 1.384(3) |
| O3 | C4 | 1.4316(19) |  | C8 | C9 | 1.389(2) |
| C1 | C2 | 1.525(2) |  | C9 | C10 | 1.377(3) |
| C1 | C3 | 1.527(2) |  | C10 | C11 | 1.385(3) |
| C1 | C4 | 1.537(2) |  | C11 | C12 | 1.393(2) |
| P1 | O1 | 1.4687(14) |  | C4 | C5 | 1.529(2) |

Table SI 17: Bond Angles for 3-hydroxy-2,3-dimethylbutan-2-yl phenylphosphinate **2a**.

| **Atom** | **Atom** | **Atom** | **Angle/˚** |  | **Atom** | **Atom** | **Atom** | **Angle/˚** |
| --- | --- | --- | --- | --- | --- | --- | --- | --- |
| O1 | P1 | O2 | 115.96(7) |  | O3 | C4 | C6 | 109.20(14) |
| O1 | P1 | C7 | 112.49(8) |  | C5 | C4 | C1 | 112.73(13) |
| O2 | P1 | C7 | 102.31(7) |  | C5 | C4 | C6 | 109.44(14) |
| C1 | O2 | P1 | 127.79(9) |  | C6 | C4 | C1 | 111.06(14) |
| O2 | C1 | C2 | 103.55(12) |  | C8 | C7 | P1 | 118.62(13) |
| O2 | C1 | C3 | 110.55(12) |  | C12 | C7 | P1 | 121.12(12) |
| O2 | C1 | C4 | 105.96(12) |  | C12 | C7 | C8 | 120.25(15) |
| C2 | C1 | C3 | 110.37(14) |  | C9 | C8 | C7 | 119.90(16) |
| C2 | C1 | C4 | 113.87(14) |  | C10 | C9 | C8 | 119.71(16) |
| C3 | C1 | C4 | 112.09(13) |  | C9 | C10 | C11 | 120.62(16) |
| O3 | C4 | C1 | 105.05(13) |  | C10 | C11 | C12 | 120.08(18) |
| O3 | C4 | C5 | 109.23(13) |  | C7 | C12 | C11 | 119.44(16) |

Table SI 18: Torsion Angles for 3-hydroxy-2,3-dimethylbutan-2-yl phenylphosphinate **2a**.

| **A** | **B** | **C** | **D** | **Angle/˚** |  | **A** | **B** | **C** | **D** | **Angle/˚** |
| --- | --- | --- | --- | --- | --- | --- | --- | --- | --- | --- |
| P1 | O2 | C1 | C2 | 139.29(12) |  | C2 | C1 | C4 | O3 | 176.46(13) |
| P1 | O2 | C1 | C3 | 21.1(2) |  | C2 | C1 | C4 | C5 | 57.62(18) |
| P1 | O2 | C1 | C4 | -100.59(14) |  | C2 | C1 | C4 | C6 | -65.60(17) |
| P1 | C7 | C8 | C9 | -178.62(12) |  | C3 | C1 | C4 | O3 | -57.35(16) |
| P1 | C7 | C12 | C11 | 178.75(13) |  | C3 | C1 | C4 | C5 | -176.19(13) |
| O1 | P1 | O2 | C1 | -87.85(14) |  | C3 | C1 | C4 | C6 | 60.58(17) |
| O1 | P1 | C7 | C8 | -13.00(15) |  | C7 | P1 | O2 | C1 | 149.36(13) |
| O1 | P1 | C7 | C12 | 167.92(13) |  | C7 | C8 | C9 | C10 | -0.2(2) |
| O2 | P1 | C7 | C8 | 112.11(13) |  | C8 | C7 | C12 | C11 | -0.3(2) |
| O2 | P1 | C7 | C12 | -66.96(14) |  | C8 | C9 | C10 | C11 | -0.3(3) |
| O2 | C1 | C4 | O3 | 63.32(14) |  | C9 | C10 | C11 | C12 | 0.4(3) |
| O2 | C1 | C4 | C5 | -55.52(16) |  | C10 | C11 | C12 | C7 | -0.1(3) |
| O2 | C1 | C4 | C6 | -178.75(13) |  | C12 | C7 | C8 | C9 | 0.5(2) |

Table SI 19: Hydrogen Atom Coordinates (Å×10^4^) and Isotropic Displacement Parameters (Å^2^×10^3^) for 3-hydroxy-2,3‑dimethylbutan-2-yl phenylphosphinate **2a**.

| **Atom** | ***x*** | ***y*** | ***z*** | **U(eq)** |
| --- | --- | --- | --- | --- |
| H1 | 3940(30) | 3190(8) | 4580(30) | 21(5) |
| H3 | 340(40) | 3231(10) | 6300(30) | 39(7) |
| H2A | 8179.77 | 3520.45 | 10327.75 | 31 |
| H2B | 6664.68 | 3311.8 | 11350.49 | 31 |
| H2C | 6327.56 | 3890.25 | 10498.11 | 31 |
| H3A | 4770.54 | 2615.28 | 7101.99 | 28 |
| H3B | 6052.06 | 2536.35 | 9233.86 | 28 |
| H3C | 7228.43 | 2793.34 | 7998.79 | 28 |
| H5A | 2284.75 | 4107.23 | 7784.47 | 30 |
| H5B | 2791.98 | 3987.58 | 9887.96 | 30 |
| H5C | 475.3 | 3854.21 | 8409.79 | 30 |
| H6A | 952.65 | 2960.34 | 9697.65 | 40 |
| H6B | 3391.82 | 3046.55 | 11023.12 | 40 |
| H6C | 2797.7 | 2582.8 | 9555.01 | 40 |
| H8 | 6757.64 | 4412.04 | 3638.65 | 20 |
| H9 | 5193.1 | 5174.85 | 2047.21 | 26 |
| H10 | 1592.32 | 5353.16 | 1453.57 | 28 |
| H11 | -481.38 | 4774.91 | 2410.79 | 30 |
| H12 | 1069.14 | 4011.23 | 4017.9 | 24 |

# 11 Computational details

Initial conformational sampling and ranking were performed using the *CREST* program in combination with *CENSO*, employing default settings.^[22–25]^ Solvent effects during conformer sampling were included via the ALPB implicit solvation model for either acetonitrile or tetrahydrofuran (THF), as specified.

All further calculations were carried out using the ORCA package.^[26,27]^ Geometry optimisations and vibrational frequency calculations were performed using the r²SCAN-3c composite method.^[28]^ Solvent effects were accounted for using the CPCM implicit solvation model with parameters corresponding to acetonitrile or THF.^[29]^ Frequency calculations confirmed all stationary points as minima (no imaginary frequencies) or transition states (one imaginary frequency) and provided thermal corrections to the Gibbs free energies.

Final single-point energies were computed at the ωB97M-V/def2-QZVPP level of theory.^[30,31]^ Solvent effects were included using the SMD solvation model for acetonitrile or THF.^[32]^

Tight SCF convergence criteria were applied for the geometry optimisations, and the single-point calculations, and numerical integrations were performed using the DefGrid3 grid.

All reported Gibbs free energies include thermal corrections obtained from the r²SCAN-3c frequency calculations and were corrected to a standard state of 1 mol·L^−^¹ by adding 1.89 kcal·mol^−^¹.

## 11.1 Energies and thermochemical corrections

Table SI 20: Energies (E) and thermochemical corrections (G-E) in Hartree. The final Gibbs energies G are given in kcal mol^−1^ and include the standard state correction.

|  | E SMD(MeCN)-ωB97M-V7def2QZVPP | G-E CPCM(MeCN)-r2SCAN-3c | G |
| --- | --- | --- | --- |
| **1a** | -1031.081194 | 0.16416824 | -646908.34 |
| **1a'** | -1031.065581 | 0.16558224 | -646897.65 |
| **1** | -954.6021517 | 0.14132451 | -598931.35 |
| H_2_O | -76.44692295 | 0.00356367 | -47967.044 |
| **2a** | -1035.873659 | 0.24886109 | -649862.51 |
| **2a'** | -1035.864316 | 0.25157703 | -649854.94 |
| **2** | -959.3995379 | 0.2262734 | -601888.45 |

Table SI 21: Energies (E) and thermochemical corrections (G-E) in Hartree. The final Gibbs energies G are given in kcal mol^−1^ and include the standard state correction.

|  | E SMD(THF)-ωB97M-V7def2QZVPP | G-E CPCM(THF)-r2SCAN-3c | G |
| --- | --- | --- | --- |
| **1e** | -1525.898984 | 0.46462039 | -957222.66 |
| **1a** | -1031.080337 | 0.16424672 | -646907.75 |
| **1c** | -1527.04018 | 0.48208164 | -957927.82 |
| **1d** | -1525.885078 | 0.4644028 | -957214.07 |
| **1b** | -1029.915088 | 0.14540458 | -646188.37 |
| **1f** | -1525.87755 | 0.46304215 | -957210.21 |
| **H_2_** | -1.161876971 | -0.00127049 | -727.99608 |
| **6** | -495.9399632 | 0.29105937 | -311022.51 |
| **2e** | -1530.686338 | 0.55063917 | -960172.8 |
| **2a** | -1035.87223 | 0.24904502 | -649861.5 |
| **2c** | -1531.838979 | 0.5659382 | -960886.49 |
| **2d** | -1530.681862 | 0.54957136 | -960170.66 |
| **2b** | -1034.72096 | 0.23116532 | -649150.28 |
| **2f** | -1530.688078 | 0.5489833 | -960174.93 |
| **TS_1d/1f_** | -1525.882206 | 0.46581447 | -957211.39 |
| **TS_1d/1f_** | -1525.865262 | 0.46403369 | -957201.87 |
| **TS_1c/1d_** | -1527.013621 | 0.4787794 | -957913.23 |
| **TS_2c/2d_** | -1531.810254 | 0.56611493 | -960868.35 |
| **TS_2d/2f_** | -1530.668788 | 0.55030087 | -960162 |
| **TS_2d/2e_** | -1530.666901 | 0.54929094 | -960161.45 |

## 11.2 Cartesian Coordinates

**1a** (MeCN)

```
P       -6.473419000     -1.104942000      0.843315000
O       -7.795010000     -0.217769000      0.470129000
O       -6.822017000     -2.389273000      1.544235000
O       -9.412799000     -1.744956000      2.289825000
H       -5.950584000     -1.234529000     -0.453074000
C       -5.387195000     -0.025493000      1.780987000
C       -4.772180000      1.058274000      1.144870000
C       -5.205911000     -0.244153000      3.149489000
C       -3.973732000      1.923384000      1.884165000
C       -4.409525000      0.630384000      3.882354000
C       -3.795336000      1.710096000      3.250841000
H       -4.916415000      1.225085000      0.079624000
H       -5.684979000     -1.091441000      3.632087000
H       -3.490204000      2.764064000      1.395440000
H       -4.265775000      0.467075000      4.946354000
H       -3.171455000      2.388711000      3.825813000
C       -8.539642000      0.386151000      1.496178000
C       -9.345054000     -0.388552000      2.345587000
H       -8.575287000     -2.113604000      1.929901000
C      -10.152881000      0.276134000      3.272303000
H      -10.784437000     -0.321754000      3.923345000
C      -10.142128000      1.663147000      3.359570000
H      -10.777052000      2.155670000      4.090386000
C       -8.516362000      1.770777000      1.590825000
H       -7.873634000      2.326583000      0.914099000
C       -9.318877000      2.417722000      2.525154000
H       -9.301979000      3.500680000      2.595566000
```

**1a'** (MeCN)

```
O      -11.002391000      7.217143000      5.798627000
C      -10.346762000      9.900940000      5.440121000
C       -9.865382000      9.750815000      4.136902000
C       -9.883864000     10.949750000      6.238347000
C       -8.926767000     10.648184000      3.637124000
C       -8.942222000     11.843007000      5.735074000
C       -8.464814000     11.693768000      4.434843000
H      -10.257382000     11.069761000      7.252272000
H       -8.555722000     10.531215000      2.622897000
H       -8.582932000     12.656617000      6.358666000
H       -7.731628000     12.392769000      4.042088000
P      -11.569050000      8.741543000      6.114343000
O      -12.743570000      9.847018000      6.797715000
O      -12.647822000      8.545768000      4.764773000
C      -13.565731000      9.553161000      4.688543000
C      -13.618712000     10.305860000      5.860698000
C      -14.397133000      9.819246000      3.617466000
C      -14.503595000     11.359309000      5.999445000
C      -15.294649000     10.887169000      3.747854000
C      -15.346657000     11.643174000      4.917250000
H      -14.349078000      9.221836000      2.712231000
H      -14.539119000     11.938604000      6.917006000
H      -15.956270000     11.126659000      2.920772000
H      -16.049540000     12.467307000      4.995379000
H      -10.221933000      8.937387000      3.510130000
H      -11.170357000      6.909823000      4.894640000
H      -11.193846000      8.543126000      7.457891000
```

**1** (MeCN)

```
C        8.557021000      7.080287000      1.882113000
C        8.619973000      8.005116000      2.928739000
C        9.510180000      7.126353000      0.856599000
C        9.614368000      8.981287000      2.934002000
C       10.495908000      8.107864000      0.862142000
C       10.548049000      9.036640000      1.901454000
H        9.482509000      6.397057000      0.048150000
H        9.658122000      9.699931000      3.747872000
H       11.227890000      8.143434000      0.060037000
H       11.322557000      9.798764000      1.909747000
P        7.298759000      5.736756000      1.787000000
O        6.280085000      6.212844000      3.057060000
O        6.277015000      6.400301000      0.590804000
C        5.448438000      7.323094000      1.200589000
C        5.442405000      7.212791000      2.591205000
C        4.649906000      8.242721000      0.548089000
C        4.635538000      8.012046000      3.377215000
C        3.831832000      9.062126000      1.335721000
C        3.824008000      8.948393000      2.724974000
H        4.661579000      8.316399000     -0.534981000
H        4.641006000      7.912161000      4.458293000
H        3.196168000      9.797248000      0.851259000
H        3.183975000      9.596763000      3.315729000
H        7.898673000      7.961328000      3.739526000
```

H_2_O (MeCN)

```
O       -8.049676000     -1.119515000      0.764011000
H       -8.049676000     -0.362298000      1.360441000
H       -8.049676000     -1.876732000      1.360441000
```

**2a** (MeCN)

```
P       -1.567515000     -4.144191000     -2.947722000
O       -2.459545000     -3.367613000     -1.860492000
O       -2.015974000     -5.541022000     -3.290523000
O       -4.722193000     -5.382896000     -2.516889000
H       -1.520941000     -3.291271000     -4.068800000
C        0.063858000     -4.056051000     -2.189064000
C        0.724654000     -2.827665000     -2.086697000
C        0.661323000     -5.222386000     -1.706067000
C        1.981048000     -2.769298000     -1.493408000
C        1.918109000     -5.155938000     -1.110682000
C        2.575717000     -3.932327000     -1.004398000
H        0.261225000     -1.921411000     -2.470513000
H        0.145986000     -6.174373000     -1.799801000
H        2.497889000     -1.817365000     -1.413431000
H        2.385418000     -6.060855000     -0.733156000
H        3.558001000     -3.884138000     -0.542490000
C       -3.925541000     -3.112312000     -1.956059000
C       -4.679546000     -4.398918000     -1.471402000
H       -3.803263000     -5.608710000     -2.763955000
C       -4.020413000     -5.015375000     -0.232834000
H       -3.928819000     -4.295457000      0.585826000
H       -4.639984000     -5.848913000      0.112286000
H       -3.023179000     -5.398283000     -0.466511000
C       -4.127600000     -1.922019000     -1.025580000
H       -3.469719000     -1.107004000     -1.341041000
H       -5.159853000     -1.568346000     -1.081454000
H       -3.895878000     -2.173970000      0.012450000
C       -6.142901000     -4.077002000     -1.166610000
H       -6.669215000     -5.017967000     -0.980781000
H       -6.234021000     -3.452868000     -0.274524000
H       -6.631646000     -3.571435000     -2.004731000
C       -4.280618000     -2.723030000     -3.384973000
H       -3.704704000     -1.840176000     -3.680141000
H       -4.094321000     -3.532770000     -4.095547000
H       -5.342357000     -2.469872000     -3.445196000
```

**2a'** (MeCN)

```
P      -14.703884000      8.378542000     -6.677875000
O      -15.940680000      8.013679000     -7.727778000
O      -15.250282000      7.116424000     -5.633197000
C      -13.050200000      7.624429000     -6.951422000
C      -12.142773000      8.353231000     -7.728685000
C      -12.651048000      6.387497000     -6.427926000
C      -10.863964000      7.862345000     -7.979110000
C      -11.372768000      5.897807000     -6.678989000
C      -10.476686000      6.632446000     -7.453755000
H      -12.435725000      9.314586000     -8.144747000
H      -10.172337000      8.440293000     -8.585723000
H      -11.075148000      4.936777000     -6.268242000
H       -9.479883000      6.245815000     -7.648417000
C      -16.697409000      6.799779000     -7.446709000
C      -16.594051000      6.679330000     -5.899082000
H      -13.345345000      5.809605000     -5.828724000
C      -17.572531000      7.602060000     -5.168140000
H      -17.532033000      8.624419000     -5.557489000
H      -18.600511000      7.236775000     -5.254461000
H      -17.304064000      7.625190000     -4.107009000
C      -18.108871000      7.008013000     -7.970846000
H      -18.548289000      7.931367000     -7.587041000
H      -18.095546000      7.055889000     -9.064214000
H      -18.743594000      6.166379000     -7.674206000
C      -16.737273000      5.254586000     -5.384030000
H      -16.686357000      5.252612000     -4.290601000
H      -17.705896000      4.838476000     -5.680902000
H      -15.943413000      4.610671000     -5.769938000
C      -16.016381000      5.651625000     -8.185237000
H      -15.931024000      5.913165000     -9.244628000
H      -15.013644000      5.461491000     -7.791186000
H      -16.605873000      4.733406000     -8.105039000
O      -14.723810000      9.464031000     -5.419186000
H      -14.912928000      9.015948000     -4.580275000
H      -14.444365000      9.491740000     -7.529112000
```

**2** (MeCN)

```
P        1.084907000      6.871553000     -4.860722000
O        1.885572000      8.046454000     -3.983259000
O        1.920081000      5.598353000     -4.189119000
C       -0.537042000      6.867490000     -3.966521000
C       -0.998150000      7.969622000     -3.241186000
C       -1.368141000      5.753612000     -4.127350000
C       -2.265284000      7.945555000     -2.662445000
C       -2.627217000      5.724533000     -3.534081000
C       -3.078424000      6.822214000     -2.801926000
H       -0.359049000      8.839653000     -3.114490000
H       -2.616379000      8.804039000     -2.095804000
H       -3.258883000      4.847397000     -3.646334000
H       -4.064169000      6.803330000     -2.345076000
C        3.052824000      7.485725000     -3.290215000
C        2.644395000      6.012364000     -2.975393000
H       -1.027235000      4.897630000     -4.708327000
C        3.825754000      5.065800000     -2.834991000
H        3.464620000      4.065226000     -2.578647000
H        4.403090000      5.003695000     -3.759681000
H        4.483453000      5.408778000     -2.029286000
C        4.243289000      7.583655000     -4.240627000
H        4.344838000      8.621887000     -4.569998000
H        5.170116000      7.290547000     -3.738476000
H        4.105438000      6.949545000     -5.122264000
C        1.722393000      5.885301000     -1.766491000
H        1.287541000      4.881070000     -1.758849000
H        2.292585000      6.019052000     -0.841856000
H        0.911078000      6.615758000     -1.785619000
C        3.287248000      8.333865000     -2.051148000
H        4.091537000      7.897207000     -1.449490000
H        3.591390000      9.341853000     -2.348551000
H        2.386498000      8.406169000     -1.438028000
```

**1e** (THF)

```
B       -0.042895000     -0.308718000     -1.207129000
P       -2.688019000     -0.062309000     -0.775386000
O       -0.241247000      0.201878000      0.197988000
O       -1.575978000     -0.410569000     -1.772433000
O       -2.695225000      1.475402000     -0.279999000
H       -2.701636000     -0.843666000      0.387872000
C       -4.275671000     -0.235337000     -1.568294000
C       -4.390011000     -0.080434000     -2.953508000
C       -5.399773000     -0.512338000     -0.783980000
C       -5.639793000     -0.200236000     -3.549740000
C       -6.644691000     -0.630896000     -1.391452000
C       -6.763077000     -0.473960000     -2.770932000
H       -3.509684000      0.126122000     -3.555107000
H        1.966361000     -1.393796000      0.449268000
H       -5.737196000     -0.083310000     -4.624827000
H       -7.521102000     -0.849404000     -0.789004000
H       -7.736707000     -0.569699000     -3.243128000
C       -0.408894000      1.490297000      0.569152000
C       -1.652161000      2.114453000      0.402811000
C        0.859727000      2.899226000     -3.493187000
C        1.281216000      2.173226000     -4.770379000
C        2.021785000      0.880136000     -4.429974000
C        1.189103000     -0.019224000     -3.510980000
C        0.755188000      0.686253000     -2.210214000
C        0.022226000      1.990620000     -2.586908000
H        0.297364000      3.810295000     -3.739122000
H        1.759980000      3.218999000     -2.946525000
H        1.906721000      2.823033000     -5.396639000
H        0.383365000      1.930687000     -5.358933000
H        2.290564000      0.340266000     -5.348178000
H        2.964950000      1.130766000     -3.920709000
H        1.760567000     -0.926811000     -3.284442000
H        0.286961000     -0.348074000     -4.052940000
H        1.681837000      0.981763000     -1.680465000
H       -0.264517000      2.552992000     -1.691740000
H       -0.912840000      1.735426000     -3.109020000
C        1.636118000     -4.164629000      0.396746000
C        0.196658000     -4.168903000     -0.121528000
C       -0.367050000     -2.746412000     -0.209575000
C        0.503782000     -1.823877000     -1.080780000
C        1.950545000     -1.847263000     -0.554473000
C        2.522566000     -3.265550000     -0.467181000
H        2.036235000     -5.186712000      0.425910000
H        1.644410000     -3.789334000      1.431364000
H       -0.437589000     -4.793165000      0.522552000
H        0.178233000     -4.623584000     -1.123664000
H       -1.390910000     -2.789836000     -0.606932000
H       -0.437529000     -2.328151000      0.806575000
H        0.515064000     -2.268706000     -2.091757000
H        2.598242000     -1.224285000     -1.185491000
H       -5.303116000     -0.638728000      0.291805000
H        3.545657000     -3.242857000     -0.067905000
H        2.585323000     -3.689297000     -1.481102000
C        0.605855000      2.208538000      1.201627000
H        1.570162000      1.725356000      1.332462000
C        0.381811000      3.509152000      1.646785000
H        1.184591000      4.054641000      2.134313000
C       -1.890765000      3.404188000      0.845612000
H       -2.868783000      3.848777000      0.687458000
C       -0.863550000      4.107684000      1.472330000
H       -1.042681000      5.119895000      1.821094000
```

**1a** (THF)

```
B       -0.042895000     -0.308718000     -1.207129000
P       -2.688019000     -0.062309000     -0.775386000
O       -0.241247000      0.201878000      0.197988000
O       -1.575978000     -0.410569000     -1.772433000
O       -2.695225000      1.475402000     -0.279999000
H       -2.701636000     -0.843666000      0.387872000
C       -4.275671000     -0.235337000     -1.568294000
C       -4.390011000     -0.080434000     -2.953508000
C       -5.399773000     -0.512338000     -0.783980000
C       -5.639793000     -0.200236000     -3.549740000
C       -6.644691000     -0.630896000     -1.391452000
C       -6.763077000     -0.473960000     -2.770932000
H       -3.509684000      0.126122000     -3.555107000
H        1.966361000     -1.393796000      0.449268000
H       -5.737196000     -0.083310000     -4.624827000
H       -7.521102000     -0.849404000     -0.789004000
H       -7.736707000     -0.569699000     -3.243128000
C       -0.408894000      1.490297000      0.569152000
C       -1.652161000      2.114453000      0.402811000
C        0.859727000      2.899226000     -3.493187000
C        1.281216000      2.173226000     -4.770379000
C        2.021785000      0.880136000     -4.429974000
C        1.189103000     -0.019224000     -3.510980000
C        0.755188000      0.686253000     -2.210214000
C        0.022226000      1.990620000     -2.586908000
H        0.297364000      3.810295000     -3.739122000
H        1.759980000      3.218999000     -2.946525000
H        1.906721000      2.823033000     -5.396639000
H        0.383365000      1.930687000     -5.358933000
H        2.290564000      0.340266000     -5.348178000
H        2.964950000      1.130766000     -3.920709000
H        1.760567000     -0.926811000     -3.284442000
H        0.286961000     -0.348074000     -4.052940000
H        1.681837000      0.981763000     -1.680465000
H       -0.264517000      2.552992000     -1.691740000
H       -0.912840000      1.735426000     -3.109020000
C        1.636118000     -4.164629000      0.396746000
C        0.196658000     -4.168903000     -0.121528000
C       -0.367050000     -2.746412000     -0.209575000
C        0.503782000     -1.823877000     -1.080780000
C        1.950545000     -1.847263000     -0.554473000
C        2.522566000     -3.265550000     -0.467181000
H        2.036235000     -5.186712000      0.425910000
H        1.644410000     -3.789334000      1.431364000
H       -0.437589000     -4.793165000      0.522552000
H        0.178233000     -4.623584000     -1.123664000
H       -1.390910000     -2.789836000     -0.606932000
H       -0.437529000     -2.328151000      0.806575000
H        0.515064000     -2.268706000     -2.091757000
H        2.598242000     -1.224285000     -1.185491000
H       -5.303116000     -0.638728000      0.291805000
H        3.545657000     -3.242857000     -0.067905000
H        2.585323000     -3.689297000     -1.481102000
C        0.605855000      2.208538000      1.201627000
H        1.570162000      1.725356000      1.332462000
C        0.381811000      3.509152000      1.646785000
H        1.184591000      4.054641000      2.134313000
C       -1.890765000      3.404188000      0.845612000
H       -2.868783000      3.848777000      0.687458000
C       -0.863550000      4.107684000      1.472330000
H       -1.042681000      5.119895000      1.821094000
```

**1c** (THF)

```
B        0.816446000      4.473895000      0.162693000
P        1.510742000      1.642562000     -0.038752000
O        1.265123000      0.761820000      1.301057000
O        0.614430000      2.855089000     -0.197321000
O        1.033813000      0.441124000      3.976417000
H        2.865765000      1.929016000      0.169298000
C        1.308498000      0.548704000     -1.440758000
C        0.704987000      1.030636000     -2.606717000
C        1.772811000     -0.769863000     -1.367199000
C        0.562225000      0.183236000     -3.699914000
C        1.625260000     -1.606324000     -2.466811000
C        1.020693000     -1.130473000     -3.629592000
H        0.346111000      2.054758000     -2.650110000
H        2.237070000     -1.142222000     -0.457630000
H        0.090535000      0.549081000     -4.606913000
H        1.978611000     -2.631750000     -2.415686000
H        0.905295000     -1.789245000     -4.485648000
C        0.001394000      0.411391000      1.794802000
C       -0.054835000      0.255154000      3.183794000
C        0.682623000      4.439611000      4.186213000
C       -0.745432000      3.923467000      4.373240000
C       -1.678043000      4.491662000      3.301377000
C       -1.150469000      4.185516000      1.896070000
C        0.282665000      4.697972000      1.671556000
C        1.202474000      4.147771000      2.773805000
H        1.348114000      3.997576000      4.940707000
H        0.694199000      5.528175000      4.349557000
H       -1.114453000      4.171882000      5.377306000
H       -0.742771000      2.825370000      4.298453000
H       -2.691989000      4.087679000      3.429271000
H       -1.749967000      5.582572000      3.430114000
H       -1.821454000      4.613871000      1.137991000
H       -1.169582000      3.094426000      1.746140000
H        0.246234000      5.796122000      1.809552000
H        2.216902000      4.553488000      2.662740000
H        1.292081000      3.055004000      2.657210000
C        4.491038000      6.421685000     -1.534926000
C        4.087060000      6.729983000     -0.091842000
C        2.635542000      6.321910000      0.182955000
C        2.362761000      4.840477000     -0.138534000
C        2.793882000      4.557476000     -1.588351000
C        4.248789000      4.947224000     -1.865303000
H        5.543075000      6.686390000     -1.704350000
H        3.892148000      7.044016000     -2.216995000
H        4.231426000      7.797517000      0.123279000
H        4.748239000      6.175389000      0.591469000
H        2.385689000      6.533908000      1.230537000
H        1.964375000      6.946364000     -0.429468000
H        3.035300000      4.256658000      0.521255000
H        2.647742000      3.497394000     -1.846823000
H        2.133807000      5.123093000     -2.265387000
H        4.509924000      4.743950000     -2.912847000
H        4.912485000      4.325497000     -1.244869000
H        1.786554000      0.709449000      3.428891000
H        0.061008000      4.978002000     -0.662831000
C       -1.266363000     -0.097091000      3.772944000
H       -1.305439000     -0.210381000      4.852379000
C       -2.395859000     -0.284903000      2.983874000
H       -3.336279000     -0.554001000      3.455253000
C       -1.119115000      0.218309000      1.000860000
H       -1.060163000      0.340211000     -0.076185000
C       -2.326936000     -0.129486000      1.601690000
H       -3.207511000     -0.275244000      0.984623000
```

**1d** (THF)

```
B       -0.011027000      0.308306000     -2.607356000
P        2.614126000      1.237508000     -2.741207000
O        2.848437000     -0.321863000     -2.177553000
O        1.007443000      1.156044000     -2.218530000
O        4.403462000      1.325438000     -3.047480000
H        2.788426000      2.383939000     -1.960253000
C        2.240707000      1.534610000     -4.500299000
C        1.205397000      2.410471000     -4.842862000
C        2.952519000      0.873302000     -5.505687000
C        0.894736000      2.630305000     -6.181807000
C        2.606729000      1.063298000     -6.839149000
C        1.584412000      1.947416000     -7.179716000
H        0.645322000      2.926453000     -4.068748000
H       -0.661400000      1.511611000     -0.142138000
H        0.102434000      3.326455000     -6.441637000
H        3.145397000      0.526953000     -7.614977000
H        1.327813000      2.104633000     -8.223639000
C        4.127255000     -0.817761000     -2.335694000
C        5.006428000      0.142527000     -2.832314000
C       -0.596072000     -2.019514000     -5.751715000
C       -0.794496000     -3.339412000     -5.002850000
C        0.142644000     -3.435559000     -3.796658000
C       -0.033219000     -2.236244000     -2.860564000
C        0.183011000     -0.900444000     -3.609904000
C       -0.774389000     -0.819320000     -4.817513000
H       -1.296313000     -1.950660000     -6.594514000
H        0.419175000     -1.992450000     -6.175923000
H       -0.635022000     -4.189727000     -5.678231000
H       -1.836250000     -3.400371000     -4.653501000
H       -0.035750000     -4.370483000     -3.249343000
H        1.184910000     -3.463573000     -4.148467000
H        0.667195000     -2.313243000     -2.017924000
H       -1.048023000     -2.257023000     -2.435279000
H        1.205382000     -0.930380000     -4.015026000
H       -0.602022000      0.113548000     -5.371312000
H       -1.815313000     -0.790418000     -4.461767000
C       -3.298263000      2.386514000     -0.457244000
C       -3.345000000      2.310335000     -1.985286000
C       -1.970496000      1.960297000     -2.562786000
C       -1.424040000      0.639741000     -1.972538000
C       -1.385208000      0.737045000     -0.434526000
C       -2.759124000      1.086362000      0.144482000
H       -4.295673000      2.608050000     -0.056285000
H       -2.642647000      3.218678000     -0.159776000
H       -3.699275000      3.261890000     -2.403147000
H       -4.068107000      1.538469000     -2.287857000
H       -2.028120000      1.894668000     -3.658519000
H       -1.265042000      2.772647000     -2.330866000
H       -2.143435000     -0.153231000     -2.236196000
H       -1.025729000     -0.208632000     -0.005556000
H        3.764529000      0.197852000     -5.253476000
H       -2.700194000      1.169753000      1.237675000
H       -3.460833000      0.268155000     -0.076243000
C        6.338495000     -0.178436000     -3.049309000
H        7.030310000      0.563579000     -3.436017000
C        6.752604000     -1.483242000     -2.752375000
H        7.791656000     -1.755655000     -2.913675000
C        4.514771000     -2.106245000     -2.042330000
H        3.802709000     -2.830549000     -1.658786000
C        5.861162000     -2.433488000     -2.259340000
H        6.206169000     -3.438931000     -2.039246000
```

**1b** (THF)

```
P      -26.775161000      3.296693000      7.166319000
O      -25.807067000      2.742451000      8.390027000
O      -27.024776000      4.753978000      7.164486000
O      -25.807486000      2.738434000      5.944232000
C      -28.238899000      2.273637000      7.167730000
C      -29.497167000      2.882401000      7.169319000
C      -28.122632000      0.878145000      7.167417000
C      -30.640737000      2.090396000      7.170613000
C      -29.270750000      0.096776000      7.168712000
C      -30.527387000      0.702407000      7.170314000
H      -29.573960000      3.965402000      7.169468000
H      -31.620467000      2.558479000      7.171828000
H      -29.186703000     -0.985796000      7.168433000
H      -31.422601000      0.086948000      7.171305000
C      -24.622979000      2.246778000      7.863366000
C      -24.623152000      2.244557000      6.472133000
H      -27.144377000      0.404027000      7.166110000
C      -23.542694000      1.795490000      8.591189000
H      -23.555030000      1.805718000      9.676047000
C      -22.441339000      1.325666000      7.865580000
H      -21.573743000      0.957577000      8.404192000
C      -23.543111000      1.790968000      5.745402000
H      -23.555791000      1.797729000      4.660522000
C      -22.441538000      1.323443000      6.472169000
H      -21.574107000      0.953628000      5.934477000
```

**1f** (THF)

```
B        4.993276000      8.741771000     -5.109849000
P        2.496975000      7.620159000     -4.526694000
O        3.072128000      6.267591000     -3.807707000
O        3.425346000      8.807139000     -4.493174000
O        2.272773000      6.952575000     -6.003687000
C        0.925048000      8.035408000     -3.824655000
C       -0.059756000      7.043892000     -3.723159000
C        0.680009000      9.341782000     -3.386879000
C       -1.294315000      7.368390000     -3.178031000
C       -0.561438000      9.651499000     -2.843995000
C       -1.543465000      8.668926000     -2.739846000
H        0.137067000      6.031293000     -4.065850000
H        6.031944000      6.996154000     -3.139668000
H       -2.063398000      6.606780000     -3.094171000
H       -0.761231000     10.662177000     -2.501556000
H       -2.511548000      8.917255000     -2.314072000
C        3.310939000      5.295121000     -4.778571000
C        2.854847000      5.685766000     -6.030462000
C        4.929511000     12.251720000     -7.058847000
C        3.931230000     11.899677000     -8.164034000
C        4.120175000     10.453751000     -8.629098000
C        4.015587000      9.481408000     -7.449822000
C        5.012161000      9.804489000     -6.323846000
C        4.834408000     11.268642000     -5.886523000
H        4.765647000     13.281638000     -6.712703000
H        5.948861000     12.213570000     -7.472759000
H        4.033970000     12.592916000     -9.009544000
H        2.908159000     12.020253000     -7.775859000
H        3.381016000     10.203994000     -9.402893000
H        5.113483000     10.352631000     -9.092407000
H        4.164222000      8.447902000     -7.794392000
H        2.989794000      9.530037000     -7.050517000
H        6.021649000      9.722802000     -6.771246000
H        5.581657000     11.529999000     -5.125480000
H        3.848691000     11.380837000     -5.405830000
C        8.219074000      8.340497000     -2.021501000
C        8.369448000      9.402891000     -3.112134000
C        7.413640000      9.140952000     -4.281051000
C        5.942054000      9.038816000     -3.839676000
C        5.816839000      7.992721000     -2.718935000
C        6.767291000      8.255036000     -1.546380000
H        8.888621000      8.557087000     -1.178486000
H        8.522671000      7.363290000     -2.427141000
H        9.408278000      9.439154000     -3.468013000
H        8.144964000     10.391118000     -2.682427000
H        7.528631000      9.931907000     -5.033871000
H        7.703352000      8.197856000     -4.774320000
H        5.669253000     10.016813000     -3.398762000
H        4.782937000      7.955319000     -2.350416000
H        1.451890000     10.099916000     -3.471831000
H        6.661757000      7.471513000     -0.783276000
H        6.491506000      9.206274000     -1.065916000
H        5.108800000      7.583471000     -5.511679000
C        3.926283000      4.081419000     -4.570336000
H        4.283899000      3.793033000     -3.587600000
C        4.064393000      3.249543000     -5.688117000
H        4.540494000      2.281548000     -5.567741000
C        2.989944000      4.883281000     -7.140866000
H        2.635061000      5.205198000     -8.114127000
C        3.606678000      3.641591000     -6.944828000
H        3.729932000      2.975801000     -7.793182000
```

H_2_ (THF)

```
H        0.194083000     -1.902491000     -0.756842000
H       -0.037319000     -1.384191000     -0.278725000
```

**6** (THF)

```
B      -17.538790000     -4.735122000      3.849614000
H      -19.633847000     -3.087982000      3.745640000
C      -13.822808000     -3.573340000      4.638562000
C      -13.250102000     -4.985427000      4.780910000
C      -14.245388000     -5.914938000      5.478343000
C      -15.592158000     -5.944934000      4.745942000
C      -16.187245000     -4.513217000      4.601846000
C      -15.167684000     -3.596685000      3.907537000
H      -13.112364000     -2.926877000      4.107118000
H      -13.963469000     -3.138240000      5.639252000
H      -12.303288000     -4.961142000      5.335266000
H      -13.024559000     -5.385101000      3.780953000
H      -13.836850000     -6.931851000      5.543700000
H      -14.408712000     -5.565406000      6.508019000
H      -16.293434000     -6.599495000      5.280747000
H      -15.443339000     -6.381665000      3.745920000
H      -16.361667000     -4.143473000      5.627444000
H      -15.572021000     -2.578627000      3.829613000
H      -15.014543000     -3.953742000      2.877599000
C      -21.827534000     -4.483596000      4.780454000
C      -21.255178000     -5.895831000      4.638152000
C      -19.910224000     -5.872842000      3.907259000
C      -18.890495000     -4.956590000      4.601688000
C      -19.485230000     -3.524710000      4.745694000
C      -20.832074000     -3.554335000      5.477971000
H      -22.774400000     -4.507645000      5.334734000
H      -22.052896000     -4.083862000      3.780481000
H      -21.965739000     -6.542102000      4.106629000
H      -21.114734000     -6.330980000      5.638851000
H      -19.506155000     -6.891009000      3.829366000
H      -20.063160000     -5.515732000      2.877309000
H      -18.716320000     -5.326375000      5.627312000
H      -18.783838000     -2.870300000      5.280537000
H      -21.240357000     -2.537315000      5.543278000
H      -20.668938000     -3.903896000      6.507668000
H      -17.538821000     -4.734603000      2.641654000
```

**2e** (THF)

```
B        0.344607000      0.059927000     -5.832874000
P       -2.297916000     -0.116416000     -5.503105000
O        0.116256000      0.517681000     -4.447162000
O       -1.162810000      0.088329000     -6.517225000
O       -2.625809000      1.092567000     -4.525646000
H       -2.077522000     -1.243204000     -4.696428000
C       -3.831469000     -0.341899000     -6.403113000
C       -3.786988000     -0.700735000     -7.754334000
C       -5.060243000     -0.203776000     -5.747673000
C       -4.974248000     -0.914185000     -8.447447000
C       -6.240811000     -0.419360000     -6.449423000
C       -6.197973000     -0.773420000     -7.796990000
H       -2.830327000     -0.805424000     -8.257268000
H       -0.171793000     -2.248974000     -3.986703000
H       -4.942733000     -1.188222000     -9.497797000
H       -7.195978000     -0.307922000     -5.944863000
H       -7.123004000     -0.939412000     -8.342049000
C       -0.333112000      1.751607000     -3.887085000
C       -1.783420000      1.485126000     -3.354643000
C        2.265378000      1.293822000     -9.140811000
C        3.626099000      1.610231000     -8.516230000
C        3.459617000      2.212230000     -7.119180000
C        2.618445000      1.299403000     -6.220990000
C        1.240099000      0.982973000     -6.827678000
C        1.438362000      0.378821000     -8.230362000
H        2.400589000      0.833130000    -10.129137000
H        1.716135000      2.234440000     -9.299865000
H        4.197833000      2.289661000     -9.162281000
H        4.208990000      0.679733000     -8.437627000
H        4.443035000      2.399572000     -6.666402000
H        2.957476000      3.188190000     -7.206462000
H        2.503971000      1.755368000     -5.228730000
H        3.167439000      0.357201000     -6.061877000
H        0.731289000      1.945695000     -6.995689000
H        0.464247000      0.176753000     -8.696152000
H        1.954571000     -0.590021000     -8.148288000
C        0.799006000     -4.477448000     -5.382838000
C        0.771032000     -3.850168000     -6.777978000
C        0.128936000     -2.459602000     -6.742374000
C        0.845724000     -1.509362000     -5.762932000
C        0.858728000     -2.159781000     -4.366456000
C        1.497337000     -3.552920000     -4.383392000
H        1.292466000     -5.458143000     -5.410498000
H       -0.235513000     -4.651310000     -5.048696000
H        0.233411000     -4.504632000     -7.478074000
H        1.801817000     -3.759596000     -7.153693000
H        0.105549000     -2.030858000     -7.751403000
H       -0.924014000     -2.576396000     -6.437978000
H        1.903816000     -1.498184000     -6.085532000
H        1.388729000     -1.515248000     -3.654706000
H       -5.092872000      0.076382000     -4.698606000
H        1.478813000     -3.997306000     -3.378497000
H        2.556465000     -3.454755000     -4.667751000
C       -1.835984000      0.358206000     -2.330810000
H       -1.216932000     -0.491504000     -2.627451000
H       -1.477934000      0.725166000     -1.364860000
H       -2.872581000      0.030470000     -2.201996000
C        0.604484000      2.059911000     -2.712171000
H        0.265874000      2.923083000     -2.131103000
H        0.695467000      1.195459000     -2.049628000
H        1.597767000      2.287501000     -3.110898000
C       -2.505320000      2.715079000     -2.823035000
H       -3.482954000      2.420598000     -2.428785000
H       -1.933852000      3.168769000     -2.009351000
H       -2.659141000      3.459598000     -3.606738000
C       -0.322901000      2.941998000     -4.849035000
H        0.688271000      3.096583000     -5.228996000
H       -1.000012000      2.797752000     -5.694937000
H       -0.616303000      3.851170000     -4.316435000
```

**2a** (THF)

```
P        1.009517000      0.376357000     -1.077148000
O        0.121485000     -0.425296000     -2.149508000
O        0.566030000      1.779256000     -0.761184000
O       -2.150824000      1.592204000     -1.507517000
H        1.050757000     -0.455071000      0.061232000
C        2.642897000      0.269133000     -1.830716000
C        3.266933000     -0.972474000     -1.989261000
C        3.280842000      1.438564000     -2.249251000
C        4.527012000     -1.040173000     -2.572994000
C        4.541265000      1.362981000     -2.835896000
C        5.162301000      0.126769000     -2.997385000
H        2.771149000     -1.882155000     -1.657854000
H        2.792044000      2.399213000     -2.112613000
H        5.014936000     -2.002574000     -2.697105000
H        5.039587000      2.270660000     -3.163763000
H        6.147311000      0.071076000     -3.452539000
C       -1.343949000     -0.677635000     -2.054196000
C       -2.099025000      0.605680000     -2.547651000
H       -1.235015000      1.837385000     -1.268782000
C       -1.435915000      1.217501000     -3.786510000
H       -1.336462000      0.493495000     -4.600724000
H       -2.056973000      2.046763000     -4.138932000
H       -0.441826000      1.606345000     -3.549753000
C       -1.544775000     -1.874602000     -2.976633000
H       -0.885369000     -2.686725000     -2.656811000
H       -2.576484000     -2.229508000     -2.918679000
H       -1.313075000     -1.628980000     -4.016188000
C       -3.560301000      0.278085000     -2.857900000
H       -4.087744000      1.217257000     -3.048693000
H       -3.646600000     -0.349861000     -3.747864000
H       -4.051701000     -0.224208000     -2.019360000
C       -1.700989000     -1.055861000     -0.622734000
H       -1.125398000     -1.936210000     -0.319249000
H       -1.516689000     -0.239337000      0.080408000
H       -2.762880000     -1.308054000     -0.561297000
```

**2c** (THF)

```
B       -1.294088000     -0.761466000     -2.566786000
P        0.961592000      0.035674000     -1.220295000
O        1.449753000      1.298851000     -2.056469000
O       -0.569797000     -0.039547000     -1.306266000
O        3.812207000      0.106773000     -1.388923000
H        1.577248000     -1.124836000     -1.699092000
C        1.373384000      0.232692000      0.515541000
C        2.101547000     -0.759477000      1.175322000
C        0.915925000      1.356122000      1.211505000
C        2.378864000     -0.623028000      2.532181000
C        1.202858000      1.487773000      2.565483000
C        1.933614000      0.500375000      3.225094000
H        2.453353000     -1.633113000      0.631962000
H        0.342837000      2.124987000      0.698839000
H        2.944386000     -1.393918000      3.047339000
H        0.853677000      2.361649000      3.107628000
H        2.154704000      0.606790000      4.283397000
C        2.637022000      1.451971000     -2.935812000
C        3.901105000      1.397033000     -2.035908000
C       -3.622224000     -3.295971000     -0.483982000
C       -2.533135000     -4.203775000      0.092175000
C       -1.412909000     -4.425739000     -0.926671000
C       -0.836737000     -3.088921000     -1.405344000
C       -1.904127000     -2.152037000     -1.996029000
C       -3.037679000     -1.965824000     -0.973056000
H       -4.406645000     -3.121986000      0.265727000
H       -4.101487000     -3.808220000     -1.332469000
H       -2.960608000     -5.164279000      0.409620000
H       -2.110160000     -3.730508000      0.991692000
H       -0.623646000     -5.055399000     -0.492700000
H       -1.817871000     -4.972639000     -1.791754000
H       -0.041359000     -3.263507000     -2.144923000
H       -0.359084000     -2.593998000     -0.542135000
H       -2.337634000     -2.684203000     -2.864552000
H       -3.836883000     -1.347592000     -1.403476000
H       -2.646798000     -1.408597000     -0.106028000
C       -3.511985000      1.950729000     -5.385406000
C       -4.217760000      0.695693000     -4.867227000
C       -3.218517000     -0.301653000     -4.270045000
C       -2.348590000      0.313064000     -3.160784000
C       -1.673920000      1.588060000     -3.694186000
C       -2.668778000      2.593688000     -4.282117000
H       -4.243710000      2.668043000     -5.780648000
H       -2.854244000      1.672688000     -6.223202000
H       -4.796325000      0.224616000     -5.673989000
H       -4.938326000      0.987491000     -4.087819000
H       -3.758878000     -1.177568000     -3.887299000
H       -2.559488000     -0.671445000     -5.073972000
H       -3.034524000      0.628616000     -2.350284000
H       -1.086312000      2.063820000     -2.898222000
H       -0.952891000      1.301118000     -4.478645000
H       -2.142008000      3.476332000     -4.671373000
H       -3.336447000      2.949071000     -3.482200000
H        4.315301000      0.146485000     -0.565531000
H       -0.387305000     -0.987532000     -3.383935000
C        5.190032000      1.444899000     -2.858743000
H        5.269263000      2.371569000     -3.434804000
H        6.043945000      1.400310000     -2.174735000
H        5.254571000      0.593842000     -3.540850000
C        2.611693000      0.355002000     -3.993539000
H        3.322987000      0.596629000     -4.787883000
H        2.871787000     -0.625201000     -3.586942000
H        1.612346000      0.307148000     -4.435691000
C        3.909642000      2.503091000     -0.982089000
H        4.089897000      3.479334000     -1.440214000
H        2.968625000      2.539329000     -0.427454000
H        4.726446000      2.318474000     -0.274418000
C        2.414116000      2.812756000     -3.579062000
H        2.267746000      3.590000000     -2.825712000
H        3.267421000      3.080745000     -4.207263000
H        1.522973000      2.768649000     -4.211825000
```

**2d** (THF)

```
B        5.753808000     -3.278681000     -2.216990000
P        8.478786000     -3.156356000     -1.659538000
O        8.609841000     -3.244763000     -3.306500000
O        6.869300000     -2.563284000     -1.890785000
O        9.992384000     -3.973563000     -1.511430000
H        8.006753000     -4.072106000     -0.699992000
C        9.004858000     -1.582788000     -0.900547000
C        8.617908000     -0.358902000     -1.454404000
C        9.835054000     -1.598554000      0.222822000
C        9.075209000      0.833195000     -0.902195000
C       10.263227000     -0.402738000      0.793557000
C        9.892792000      0.814025000      0.226593000
H        7.965497000     -0.339470000     -2.321888000
H        3.789280000     -5.235081000     -1.793365000
H        8.784954000      1.780104000     -1.348942000
H       10.896071000     -0.423235000      1.676399000
H       10.238815000      1.746581000      0.664009000
C        9.772592000     -3.958675000     -3.830550000
C       10.818840000     -3.862695000     -2.680412000
C        3.187712000     -0.357853000     -3.021587000
C        2.788469000     -0.028579000     -1.581012000
C        2.684815000     -1.298830000     -0.733508000
C        3.988798000     -2.100937000     -0.778417000
C        4.396613000     -2.451853000     -2.227485000
C        4.490947000     -1.160623000     -3.062730000
H        3.290367000      0.565224000     -3.607435000
H        2.386578000     -0.947476000     -3.492077000
H        1.838086000      0.520398000     -1.563383000
H        3.548373000      0.634673000     -1.140980000
H        2.431912000     -1.044017000      0.304235000
H        1.865708000     -1.924445000     -1.118459000
H        3.887047000     -3.015676000     -0.177983000
H        4.793516000     -1.507745000     -0.317740000
H        3.583610000     -3.064363000     -2.652538000
H        4.752432000     -1.401641000     -4.102655000
H        5.309561000     -0.541119000     -2.667870000
C        4.454209000     -7.303765000     -3.552720000
C        5.419748000     -6.510720000     -4.436169000
C        5.432647000     -5.028986000     -4.050218000
C        5.780741000     -4.829021000     -2.556395000
C        4.805088000     -5.643147000     -1.681726000
C        4.792845000     -7.124275000     -2.071222000
H        4.475188000     -8.367201000     -3.823449000
H        3.428322000     -6.948584000     -3.732621000
H        5.148854000     -6.623244000     -5.494236000
H        6.434124000     -6.920754000     -4.319355000
H        6.146740000     -4.481257000     -4.679956000
H        4.439406000     -4.599129000     -4.250358000
H        6.785883000     -5.259202000     -2.407562000
H        5.071077000     -5.537127000     -0.621094000
H       10.149657000     -2.545430000      0.650952000
H        4.075465000     -7.673812000     -1.447704000
H        5.785413000     -7.555851000     -1.873207000
C       11.815477000     -5.014214000     -2.662046000
H       11.316465000     -5.972047000     -2.499762000
H       12.367027000     -5.057264000     -3.607286000
H       12.537035000     -4.857871000     -1.853988000
C        9.327263000     -5.389939000     -4.112568000
H        9.058726000     -5.911545000     -3.188772000
H        8.450449000     -5.367517000     -4.766263000
H       10.119022000     -5.951116000     -4.617991000
C       11.578224000     -2.532525000     -2.679305000
H       10.911774000     -1.672231000     -2.781362000
H       12.114987000     -2.436433000     -1.730514000
H       12.307948000     -2.505757000     -3.494579000
C       10.179584000     -3.263832000     -5.119433000
H        9.400119000     -3.398879000     -5.875692000
H       10.336143000     -2.193627000     -4.968230000
H       11.106142000     -3.704955000     -5.501464000
```

**2b** (THF)

```
P      -18.985036000      5.250129000      4.174947000
O      -18.165538000      5.914291000      2.940940000
O      -18.980279000      3.763433000      4.128133000
O      -18.186768000      5.998560000      5.365948000
C      -20.657838000      5.917568000      4.240949000
C      -21.636218000      5.326386000      3.431730000
C      -20.998028000      6.986552000      5.075996000
C      -22.936916000      5.817194000      3.444420000
C      -22.305733000      7.462992000      5.094011000
C      -23.272910000      6.884410000      4.275795000
H      -21.379279000      4.482798000      2.796686000
H      -23.691342000      5.360896000      2.810088000
H      -22.568890000      8.286827000      5.751238000
H      -24.291838000      7.261320000      4.289862000
C      -17.252197000      6.980120000      3.397122000
C      -16.909947000      6.560897000      4.866345000
H      -20.247117000      7.435857000      5.718581000
C      -15.860803000      5.459291000      4.951260000
H      -16.086523000      4.629715000      4.274405000
H      -14.870430000      5.854211000      4.706557000
H      -15.835921000      5.076170000      5.975347000
C      -16.055003000      6.968338000      2.463891000
H      -16.365469000      7.295042000      1.467246000
H      -15.294307000      7.664888000      2.831370000
H      -15.615518000      5.972147000      2.385723000
C      -16.554946000      7.723175000      5.772671000
H      -16.307802000      7.348870000      6.770259000
H      -15.678099000      8.244796000      5.375305000
H      -17.380131000      8.433418000      5.857329000
C      -17.995405000      8.306073000      3.300185000
H      -18.382048000      8.418072000      2.283144000
H      -18.834744000      8.358300000      3.998330000
H      -17.318045000      9.140126000      3.505368000
```

**2f** (THF)

```
B       -0.564875000     -0.619044000      4.837702000
P       -2.015096000     -3.003554000      3.986045000
O       -3.402964000     -2.203434000      3.916350000
O       -0.830874000     -2.072929000      4.120430000
O       -2.381444000     -3.990709000      5.191871000
C       -1.696553000     -4.041636000      2.563573000
C       -2.067112000     -5.390656000      2.555912000
C       -1.075087000     -3.474172000      1.443894000
C       -1.822498000     -6.164138000      1.426278000
C       -0.844884000     -4.253447000      0.316754000
C       -1.218213000     -5.596314000      0.307211000
H       -2.527260000     -5.837216000      3.431939000
H       -2.479746000      0.913189000      3.404516000
H       -2.100246000     -7.213959000      1.423230000
H       -0.366497000     -3.813417000     -0.553107000
H       -1.030653000     -6.204042000     -0.573530000
C       -4.456616000     -2.857748000      4.734355000
C       -3.650606000     -3.585835000      5.867838000
C        3.221952000     -1.380637000      5.950305000
C        2.943920000     -2.431869000      7.026794000
C        1.687859000     -2.072491000      7.823339000
C        0.480428000     -1.888821000      6.897996000
C        0.722790000     -0.849382000      5.790924000
C        2.012062000     -1.198583000      5.027322000
H        4.112914000     -1.657261000      5.369543000
H        3.446478000     -0.420061000      6.439182000
H        3.808248000     -2.535865000      7.696314000
H        2.795465000     -3.410016000      6.543926000
H        1.482404000     -2.844013000      8.578603000
H        1.868191000     -1.133473000      8.368878000
H       -0.401308000     -1.600948000      7.487046000
H        0.239222000     -2.860080000      6.435872000
H        0.904758000      0.114206000      6.304235000
H        2.230467000     -0.422048000      4.281951000
H        1.852665000     -2.132406000      4.464085000
C       -1.045272000      2.945017000      2.135646000
C        0.232140000      2.817559000      2.967946000
C        0.061490000      1.802365000      4.103689000
C       -0.387234000      0.417608000      3.606102000
C       -1.656607000      0.576186000      2.752828000
C       -1.486937000      1.578669000      1.607108000
H       -0.895700000      3.649918000      1.306928000
H       -1.845619000      3.360719000      2.766754000
H        0.523522000      3.797981000      3.369511000
H        1.052800000      2.484964000      2.313970000
H        1.002077000      1.717579000      4.663611000
H       -0.689882000      2.184979000      4.815005000
H        0.407952000      0.045590000      2.930676000
H       -1.967517000     -0.396009000      2.346326000
H       -0.769471000     -2.431827000      1.459520000
H       -2.420848000      1.675269000      1.036102000
H       -0.724249000      1.200997000      0.908682000
H       -1.577591000     -0.369393000      5.490303000
C       -3.268034000     -2.680718000      7.025718000
H       -2.798265000     -1.755690000      6.678535000
H       -4.151775000     -2.432586000      7.620551000
H       -2.557853000     -3.210913000      7.666277000
C       -5.356451000     -1.748782000      5.244235000
H       -4.785283000     -0.969350000      5.751999000
H       -5.896448000     -1.302000000      4.404878000
H       -6.090058000     -2.165328000      5.942118000
C       -4.303446000     -4.861664000      6.360088000
H       -3.694664000     -5.301240000      7.154929000
H       -5.289965000     -4.627993000      6.773685000
H       -4.421272000     -5.593362000      5.558263000
C       -5.222790000     -3.798365000      3.816504000
H       -5.585110000     -3.229524000      2.955712000
H       -4.600998000     -4.619964000      3.451811000
H       -6.085723000     -4.220210000      4.339212000
```

**TS_1d/1f_** (THF)

```
B       -0.103001000      0.313946000     -3.208576000
P        1.924578000      1.766752000     -3.875710000
O        1.898639000      0.001065000     -3.188102000
O        0.342775000      1.630195000     -3.667248000
O        3.072484000      2.094450000     -2.694176000
H        1.859713000      3.147067000     -4.152651000
C        2.657999000      1.250135000     -5.461031000
C        2.887581000      2.270151000     -6.392925000
C        3.003926000     -0.068099000     -5.786634000
C        3.448385000      1.978919000     -7.632361000
C        3.564748000     -0.352472000     -7.027080000
C        3.787413000      0.666756000     -7.950678000
H        2.627020000      3.298174000     -6.153812000
H        0.665944000      1.692145000     -0.837568000
H        3.619514000      2.777879000     -8.347883000
H        3.828882000     -1.377094000     -7.272667000
H        4.225253000      0.437651000     -8.918312000
C        2.907773000     -0.183456000     -2.306724000
C        3.592066000      1.000069000     -2.036407000
C       -2.289934000     -1.457306000     -5.978472000
C       -2.047767000     -2.921028000     -5.602723000
C       -0.610902000     -3.136212000     -5.121220000
C       -0.265467000     -2.193790000     -3.964632000
C       -0.484561000     -0.720877000     -4.347746000
C       -1.945391000     -0.525331000     -4.812593000
H       -3.334399000     -1.311077000     -6.283871000
H       -1.662589000     -1.195983000     -6.843604000
H       -2.267418000     -3.575513000     -6.456073000
H       -2.742216000     -3.203504000     -4.797182000
H       -0.464319000     -4.181335000     -4.818235000
H        0.081348000     -2.945262000     -5.955355000
H        0.775785000     -2.347763000     -3.652811000
H       -0.896745000     -2.442869000     -3.098019000
H        0.153548000     -0.511458000     -5.225511000
H       -2.121608000      0.520351000     -5.100191000
H       -2.623712000     -0.744314000     -3.974266000
C       -1.669209000      1.680776000      0.732646000
C       -2.642627000      1.285092000     -0.378052000
C       -1.943190000      1.261592000     -1.739811000
C       -0.724783000      0.300162000     -1.749668000
C        0.237665000      0.701267000     -0.616666000
C       -0.458073000      0.747701000      0.747195000
H       -2.173528000      1.669241000      1.707483000
H       -1.328504000      2.712850000      0.560888000
H       -3.492887000      1.979283000     -0.406503000
H       -3.051243000      0.286167000     -0.164576000
H       -2.662378000      0.986084000     -2.520566000
H       -1.593232000      2.277137000     -1.977129000
H       -1.106351000     -0.709697000     -1.523205000
H        1.077102000     -0.001031000     -0.568358000
H        2.828503000     -0.868977000     -5.078511000
H        0.255484000      1.059549000      1.521217000
H       -0.790568000     -0.267535000      1.011010000
C        4.666898000      1.044495000     -1.173395000
H        5.184186000      1.978526000     -0.977205000
C        5.051905000     -0.154355000     -0.559658000
H        5.891564000     -0.152427000      0.128515000
C        3.287592000     -1.372934000     -1.707324000
H        2.748075000     -2.291849000     -1.915488000
C        4.371521000     -1.341326000     -0.822706000
H        4.686013000     -2.260759000     -0.337846000
```

**TS_1d/1f_** (THF)

```
B        0.703756000      0.187451000      1.793426000
P       -1.002933000      0.528396000      0.359613000
O       -1.270096000      1.562502000     -1.001550000
O        0.539280000      0.870751000      0.452194000
O       -2.202780000      1.295608000      1.227801000
H       -0.789910000     -0.240338000      1.706466000
C       -1.492714000     -1.008813000     -0.461263000
C       -1.113023000     -1.254457000     -1.784821000
C       -2.230091000     -1.958630000      0.247869000
C       -1.454584000     -2.462482000     -2.382273000
C       -2.593184000     -3.152562000     -0.367254000
C       -2.197310000     -3.408550000     -1.677586000
H       -0.542310000     -0.515831000     -2.338831000
H        1.304329000     -0.182204000      4.561767000
H       -1.143881000     -2.663214000     -3.403442000
H       -3.175898000     -3.886146000      0.181938000
H       -2.468168000     -4.347557000     -2.152071000
C        2.964660000     -2.859911000      0.566006000
C        2.185980000     -4.017360000      1.195106000
C        1.616262000     -3.616750000      2.557693000
C        0.745263000     -2.363454000      2.440909000
C        1.519202000     -1.183009000      1.815207000
C        2.095314000     -1.604967000      0.451490000
H        3.344307000     -3.149524000     -0.422723000
H        3.841673000     -2.634115000      1.191262000
H        2.827195000     -4.902854000      1.292855000
H        1.355543000     -4.295592000      0.527850000
H        1.033276000     -4.443175000      2.985072000
H        2.445567000     -3.416126000      3.252539000
H        0.347337000     -2.085264000      3.424428000
H       -0.121325000     -2.596584000      1.804953000
H        2.380687000     -0.995967000      2.484977000
H        2.675581000     -0.782312000      0.014038000
H        1.268341000     -1.807437000     -0.245496000
C        1.931792000      2.387223000      5.458214000
C        2.133638000      3.030068000      4.084351000
C        2.051380000      1.986412000      2.965855000
C        0.713813000      1.208633000      3.011252000
C        0.524398000      0.577421000      4.398553000
C        0.608277000      1.621092000      5.516395000
H        1.966435000      3.151268000      6.245449000
H        2.760168000      1.689714000      5.653940000
H        3.100310000      3.549089000      4.044303000
H        1.354512000      3.789596000      3.922522000
H        2.174367000      2.472567000      1.989058000
H        2.887030000      1.278203000      3.077806000
H       -0.086272000      1.957085000      2.871743000
H       -0.440246000      0.053428000      4.445317000
H       -2.519563000     -1.769291000      1.278805000
H        0.490108000      1.139430000      6.495827000
H       -0.224625000      2.331711000      5.407022000
C       -2.844282000      3.310922000     -1.688223000
C       -3.929987000      4.085099000     -1.261319000
C       -2.323168000      2.395672000     -0.791049000
H       -4.361022000      4.810265000     -1.945085000
C       -4.465091000      3.941801000      0.017315000
C       -2.865095000      2.259145000      0.482361000
H       -5.307422000      4.554454000      0.323060000
C       -3.931099000      3.013068000      0.920608000
H       -4.333401000      2.887502000      1.920727000
H       -2.421158000      3.418227000     -2.681933000
```

**TS_1c/1d_** (THF)

```
B        2.195270000      0.338134000      2.947019000
P        1.278470000     -1.505259000      4.970342000
O        1.352111000     -3.087676000      4.537304000
O        2.140685000     -0.455859000      4.201581000
O       -0.125282000     -1.391603000      3.394040000
H        0.111770000     -1.066107000      5.611615000
C        2.339613000     -1.719381000      6.434584000
C        1.803616000     -1.479292000      7.699610000
C        3.679346000     -2.105051000      6.309543000
C        2.599354000     -1.620433000      8.836032000
C        4.469939000     -2.246229000      7.443341000
C        3.930509000     -2.003841000      8.707803000
H        0.763046000     -1.178428000      7.805889000
H        4.852699000     -0.297154000      3.505951000
H        2.177613000     -1.430183000      9.818767000
H        5.509786000     -2.544633000      7.343985000
H        4.551756000     -2.113934000      9.592389000
C        0.682732000     -3.541266000      3.409862000
C       -0.126009000     -2.590784000      2.788282000
C        0.554814000      3.692312000      4.346005000
C        0.575235000      4.517927000      3.058047000
C        1.853557000      4.250799000      2.261109000
C        2.023965000      2.757946000      1.963189000
C        1.994086000      1.898665000      3.237491000
C        0.719908000      2.199698000      4.046632000
H       -0.377900000      3.868975000      4.897879000
H        1.378690000      4.020977000      4.997081000
H        0.483523000      5.587504000      3.287027000
H       -0.296336000      4.247151000      2.443081000
H        1.847162000      4.826326000      1.326040000
H        2.719250000      4.599097000      2.844368000
H        2.961874000      2.593719000      1.417304000
H        1.210816000      2.432883000      1.292720000
H        2.855284000      2.206966000      3.861192000
H        0.723247000      1.631534000      4.986793000
H       -0.155879000      1.853904000      3.472694000
C        5.758439000     -1.820066000      1.336034000
C        4.387397000     -2.185328000      0.762892000
C        3.263066000     -1.705845000      1.684860000
C        3.337069000     -0.195034000      1.960061000
C        4.730673000      0.166637000      2.514159000
C        5.858559000     -0.315615000      1.596381000
H        6.557186000     -2.144713000      0.656657000
H        5.906677000     -2.359787000      2.283878000
H        4.315444000     -3.270022000      0.607690000
H        4.271335000     -1.712595000     -0.223847000
H        2.285893000     -1.964582000      1.251694000
H        3.336652000     -2.252158000      2.638464000
H        3.229841000      0.320572000      0.989744000
H        4.808716000      1.251826000      2.663226000
H        4.103046000     -2.290350000      5.325189000
H        6.835109000     -0.068508000      2.033570000
H        5.792458000      0.220506000      0.637721000
H        0.589323000     -0.431851000      2.580304000
H        1.056322000      0.117056000      2.100238000
C       -0.820346000     -2.938505000      1.633416000
H       -1.448543000     -2.204726000      1.136652000
C       -0.691197000     -4.240085000      1.140418000
H       -1.233398000     -4.523085000      0.242673000
C        0.825836000     -4.828538000      2.937476000
H        1.469874000     -5.537253000      3.449087000
C        0.118614000     -5.176161000      1.780937000
H        0.207501000     -6.182493000      1.383753000
```

**TS_2c/2d_** (THF)

```
B        2.460374000      0.321655000      3.059915000
P        1.395913000     -1.417917000      5.071462000
O        1.119432000     -3.016315000      4.835589000
O        2.441950000     -0.536018000      4.269036000
O        0.164811000     -1.118325000      3.710174000
H        0.478670000     -0.741036000      5.901745000
C        2.583432000     -1.800387000      6.443329000
C        2.250474000     -1.437917000      7.747371000
C        3.809115000     -2.433401000      6.206544000
C        3.125091000     -1.697527000      8.804106000
C        4.682574000     -2.694174000      7.256551000
C        4.341808000     -2.325928000      8.559462000
H        1.300842000     -0.943585000      7.948848000
H        5.077184000      0.038626000      3.941969000
H        2.854249000     -1.407333000      9.815708000
H        5.632854000     -3.184378000      7.061848000
H        5.025621000     -2.529025000      9.379287000
C        0.234182000     -3.461851000      3.756659000
C       -0.703309000     -2.244854000      3.507435000
C        0.338873000      3.487861000      4.326986000
C        0.376583000      4.326989000      3.048911000
C        1.732117000      4.190002000      2.354453000
C        2.073986000      2.723458000      2.071831000
C        2.025322000      1.846652000      3.335397000
C        0.664082000      2.020704000      4.030971000
H       -0.644816000      3.569820000      4.808570000
H        1.077721000      3.884400000      5.039694000
H        0.165014000      5.380647000      3.273128000
H       -0.415236000      3.980284000      2.367625000
H        1.743673000      4.769257000      1.421427000
H        2.511171000      4.617035000      3.004040000
H        3.066084000      2.665913000      1.607263000
H        1.359084000      2.324287000      1.332722000
H        2.787960000      2.245013000      4.032731000
H        0.634583000      1.444072000      4.964617000
H       -0.125566000      1.606058000      3.385129000
C        6.514250000     -1.142069000      1.856560000
C        5.318822000     -1.649871000      1.048708000
C        4.004213000     -1.422597000      1.801398000
C        3.793528000      0.046550000      2.197068000
C        5.023664000      0.561871000      2.974144000
C        6.334554000      0.333778000      2.215573000
H        7.447011000     -1.291231000      1.297169000
H        6.599446000     -1.730182000      2.783223000
H        5.442286000     -2.715041000      0.811196000
H        5.281308000     -1.111549000      0.089502000
H        3.164244000     -1.760025000      1.178550000
H        3.997401000     -2.049194000      2.706821000
H        3.737453000      0.625244000      1.259251000
H        4.906988000      1.629915000      3.200850000
H        4.083199000     -2.719388000      5.193309000
H        7.185060000      0.688194000      2.813241000
H        6.323173000      0.929811000      1.290503000
H        0.930876000     -0.563021000      2.684930000
H        1.498427000     -0.153388000      2.121943000
C       -1.272134000     -2.187319000      2.096491000
H       -0.482951000     -2.097599000      1.345901000
H       -1.853151000     -3.091219000      1.886409000
H       -1.938836000     -1.323998000      2.006858000
C        1.117449000     -3.791793000      2.561684000
H        1.905081000     -4.481697000      2.879575000
H        0.534771000     -4.273716000      1.770985000
H        1.588895000     -2.894079000      2.154929000
C       -1.836217000     -2.153469000      4.532505000
H       -2.587711000     -2.928053000      4.353196000
H       -1.470473000     -2.259060000      5.558350000
H       -2.317101000     -1.175530000      4.434804000
C       -0.475087000     -4.709835000      4.256645000
H        0.247197000     -5.522985000      4.376153000
H       -0.967220000     -4.536577000      5.215945000
H       -1.227031000     -5.025909000      3.526161000
```

**TS_2d/2f_** (THF)

```
B        0.277271000     -0.507594000     -6.445358000
P       -2.122969000     -0.082093000     -5.446057000
O       -0.629819000      0.312102000     -4.620261000
O       -1.127610000     -0.513802000     -6.683678000
O       -2.866008000      1.102010000     -4.504655000
H       -2.425249000     -1.277295000     -4.772810000
C       -3.568014000      0.052690000     -6.602346000
C       -3.454253000      0.593419000     -7.884227000
C       -4.817848000     -0.387219000     -6.157068000
C       -4.574304000      0.694964000     -8.707279000
C       -5.933046000     -0.306052000     -6.987034000
C       -5.814225000      0.241463000     -8.263100000
H       -2.486646000      0.931933000     -8.243629000
H        0.974344000     -1.606143000     -3.839356000
H       -4.475614000      1.123236000     -9.701189000
H       -6.895902000     -0.667148000     -6.635647000
H       -6.684825000      0.311511000     -8.909675000
C       -0.682231000      1.408281000     -3.686805000
C       -2.181707000      1.420833000     -3.267788000
C        1.774698000      1.474249000     -9.460882000
C        3.182877000      1.763919000     -8.937260000
C        3.156818000      2.083865000     -7.441064000
C        2.488067000      0.958006000     -6.647051000
C        1.064547000      0.673804000     -7.145559000
C        1.104108000      0.352130000     -8.662185000
H        1.810928000      1.209275000    -10.525878000
H        1.163323000      2.384942000     -9.376925000
H        3.635821000      2.591157000     -9.498795000
H        3.817048000      0.880193000     -9.104090000
H        4.175928000      2.258220000     -7.071665000
H        2.594657000      3.016595000     -7.281051000
H        2.473978000      1.212488000     -5.578806000
H        3.095563000      0.044642000     -6.741698000
H        0.470133000      1.597750000     -7.041077000
H        0.087292000      0.180484000     -9.039636000
H        1.665464000     -0.582476000     -8.820171000
C        1.576734000     -4.275611000     -4.348027000
C        0.824752000     -4.377024000     -5.677125000
C        0.056849000     -3.087774000     -5.982175000
C        0.981410000     -1.857304000     -5.985252000
C        1.718265000     -1.770508000     -4.630582000
C        2.497866000     -3.053515000     -4.329595000
H        2.151690000     -5.192180000     -4.162472000
H        0.847160000     -4.185577000     -3.528995000
H        0.139296000     -5.234842000     -5.661319000
H        1.547541000     -4.561796000     -6.486256000
H       -0.462492000     -3.177703000     -6.945131000
H       -0.724683000     -2.948046000     -5.219476000
H        1.767252000     -2.043186000     -6.744374000
H        2.391628000     -0.905983000     -4.609112000
H       -4.921263000     -0.806745000     -5.158205000
H        2.999233000     -2.971080000     -3.355938000
H        3.286906000     -3.182632000     -5.085728000
C       -2.518145000      0.345289000     -2.235016000
H       -2.097663000     -0.628627000     -2.502809000
H       -2.135749000      0.624170000     -1.248602000
H       -3.606445000      0.250080000     -2.167374000
C        0.277028000      1.122502000     -2.540770000
H        0.173135000      1.887211000     -1.763595000
H        0.095113000      0.140835000     -2.098277000
H        1.307187000      1.149713000     -2.910386000
C       -2.689625000      2.775718000     -2.801113000
H       -3.734119000      2.688074000     -2.486250000
H       -2.102760000      3.123892000     -1.944765000
H       -2.628070000      3.520640000     -3.597348000
C       -0.269060000      2.686095000     -4.415032000
H        0.727796000      2.545566000     -4.841530000
H       -0.964086000      2.921316000     -5.227515000
H       -0.229614000      3.534700000     -3.725318000
```

**TS_2d/2e_** (THF)

```
B        0.667155000      0.163171000      1.780197000
P       -1.213953000      0.388859000      0.408592000
O       -1.538337000      1.344655000     -0.946472000
O        0.352260000      0.768639000      0.478868000
O       -2.381249000      1.155702000      1.278420000
H       -0.990402000     -0.373356000      1.717143000
C       -1.585912000     -1.181749000     -0.439818000
C       -1.170432000     -1.405863000     -1.756214000
C       -2.266373000     -2.183939000      0.252821000
C       -1.420522000     -2.633288000     -2.361291000
C       -2.543692000     -3.399166000     -0.367401000
C       -2.112101000     -3.628126000     -1.671412000
H       -0.640094000     -0.630321000     -2.300019000
H        1.360498000     -0.167174000      4.541185000
H       -1.078342000     -2.812162000     -3.376801000
H       -3.084698000     -4.170570000      0.173159000
H       -2.312110000     -4.582556000     -2.150605000
C       -2.219379000      2.572851000     -0.611776000
C       -3.187136000      2.122258000      0.520405000
C        3.009540000     -2.798662000      0.518985000
C        2.261142000     -3.982852000      1.134842000
C        1.689401000     -3.614906000      2.505751000
C        0.785636000     -2.382948000      2.411099000
C        1.527407000     -1.174077000      1.797544000
C        2.109782000     -1.563719000      0.426793000
H        3.391068000     -3.065353000     -0.475532000
H        3.884305000     -2.561914000      1.143461000
H        2.922816000     -4.854865000      1.216661000
H        1.433575000     -4.270289000      0.467789000
H        1.129250000     -4.461389000      2.924577000
H        2.517423000     -3.403338000      3.198940000
H        0.386846000     -2.127779000      3.400375000
H       -0.078038000     -2.628401000      1.776502000
H        2.381954000     -0.970558000      2.471684000
H        2.668143000     -0.720674000     -0.001075000
H        1.283746000     -1.773577000     -0.268710000
C        1.808433000      2.453671000      5.409411000
C        1.912096000      3.104484000      4.028469000
C        1.880610000      2.052103000      2.915640000
C        0.612279000      1.165761000      3.006968000
C        0.519355000      0.529152000      4.400588000
C        0.554782000      1.582119000      5.512398000
H        1.806281000      3.221705000      6.193548000
H        2.697224000      1.827252000      5.578918000
H        2.830625000      3.701665000      3.955769000
H        1.067456000      3.795655000      3.889596000
H        1.930135000      2.542256000      1.933986000
H        2.775640000      1.416117000      3.001233000
H       -0.249997000      1.844583000      2.886575000
H       -0.399566000     -0.067599000      4.479003000
H       -2.578125000     -2.017078000      1.281650000
H        0.507744000      1.098188000      6.496726000
H       -0.336308000      2.222218000      5.428045000
C       -3.590362000      3.222592000      1.485560000
H       -2.722747000      3.643835000      1.998181000
H       -4.100934000      4.023653000      0.940692000
H       -4.281113000      2.825089000      2.235365000
C       -1.197189000      3.609763000     -0.141822000
H       -0.423400000      3.709180000     -0.909045000
H       -1.670807000      4.586004000      0.000664000
H       -0.714620000      3.313436000      0.794456000
C       -4.409875000      1.373842000      0.002988000
H       -5.112877000      2.062716000     -0.474800000
H       -4.127271000      0.601217000     -0.718791000
H       -4.917398000      0.896044000      0.846536000
C       -2.928382000      3.067881000     -1.862826000
H       -2.186787000      3.394385000     -2.598373000
H       -3.540024000      2.281766000     -2.311106000
H       -3.569170000      3.922609000     -1.621990000
```

# 12 References

[1]	L. Hintermann, T. T. Dang, A. Labonne, T. Kribber, L. Xiao, P. Naumov, "The AZARYPHOS family of ligands for ambifunctional catalysis: syntheses and use in ruthenium-catalyzed anti-Markovnikov hydration of terminal alkynes" *Chemistry – A European Journal* **15**, (2009): 7167.

[2]	Y. Soltani, L. C. Wilkins, R. L. Melen, "Stoichiometric and Catalytic C-C and C-H Bond Formation with B(C_6_F_5_)_3_ via Cationic Intermediates" *Angewandte Chemie International Edition* **56**, (2017): 11995.

[3]	L. E. Longobardi, T. C. Johnstone, R. L. Falconer, C. A. Russell, D. W. Stephan, "Hydroboration of Phosphaalkynes by HB(C_6_F_5_)_2_" *Chemistry – A European Journal* **22**, (2016): 12665.

[4]	A. Abiko, "Dicyclohexylboron trifluoromethanesulfonate" *Organic Syntheses* **79**, (2002): 103.

[5]	A. Yeganeh-Salman, I. Elser, K. L. Bamford, D. Ebanks, D. W. Stephan, "Probing B-X to B-H conversions and applications in C-F bond activation catalysis" *Dalton Transactions* **51**, (2022): 17962.

[6]	D. J. St Cyr, B. A. Arndtsen, "A new use of Wittig-type reagents as 1,3-dipolar cycloaddition precursors and in pyrrole synthesis" *Journal of the American Chemical Society* **129**, (2007): 12366.

[7]	D. J. St-Cyr, M. S. T. Morin, F. Bélanger-Gariépy, B. A. Arndtsen, E. H. Krenske, K. N. Houk, "Phospha-Münchnones: electronic structures and 1,3-dipolar cycloadditions" *The Journal of Organic Chemistry* **75**, (2010): 4261.

[8]	B. Fontal, H. Goldwhite, "Configurational stability in some 3-coordinate phosphorus compounds" *Tetrahedron* **22**, (1966): 3275.

[9]	E. H. Amonoo-Neizer, S. K. Ray, R. A. Shaw, B. C. Smith, "793. Sulphoxides. Part I. The oxidation of tervalent phosphorus compounds by dimethyl sulphoxide" *Journal of the Chemical Society*, (1965): 4296.

[10]	J. Gloede, H. Groß, "Umwandlung von Trimethylphosphit in o-Phenylenmethanphosphonat" *Zeitschrift für Chemie* **24**, (1984): 391.

[11]	M. T. Boisdon, C. Malavaud, B. Tangour, J. Barrans, "Phosphoranes monocycliques a ligand hydrogene i-etude de l'equilibre de formation" *Phosphorus and Sulfur and the Related Elements* **8**, (1980): 305.

[12]	U. Gellrich, "Reversible Hydrogen Activation by a Pyridonate Borane Complex: Combining Frustrated Lewis Pair Reactivity with Boron-Ligand Cooperation" *Angewandte Chemie International Edition* **57**, (2018): 4779.

[13]	A. K. Dash, R. F. Jordan, "Vinyl C−H Activation Reactions of Vinyl Esters Mediated by B(C_6_F_5_)_3_" *Organometallics* **21**, (2002): 777.

[14]	H. Baumgarth, G. Meier, C. N. von Hahmann, T. Braun, "Reactivity of rhodium and iridium peroxido complexes towards hydrogen in the presence of B(C_6_F_5_)_3_ or [H(OEt_2_)_2_][B{3,5-(CF_3_)_2_C_6_H_3_}_4_]" *Dalton Transactions* **47**, (2018): 16299.

[15]	G. M. Sheldrick, "Crystal structure refinement with SHELXL" *Acta Crystallographica Section C: Structural chemistry* **71**, (2015): 3.

[16]	P. Müller, "Practical suggestions for better crystal structures" *Crystallography Reviews* **15**, (2009): 57.

[17]	A. Thorn, B. Dittrich, G. M. Sheldrick, "Enhanced rigid-bond restraints" *Acta Crystallographica Section A: Foundations of Crystallography* **68**, (2012): 448.

[18]	SAINT, Bruker AXS GmbH, Karlsruhe (Germany), 2016.

[19]	G. M. Sheldrick, "SADABS-Bruker Nonius Scaling and Absorption Correction., Bruker AXS Inc, Madison WI (USA), 2012.

[20]	O. V. Dolomanov, L. J. Bourhis, R. J. Gildea, J. A. K. Howard, H. Puschmann, "OLEX2 : a complete structure solution, refinement and analysis program" *Journal of Applied Crystallography* **42**, (2009): 339.

[21]	G. M. Sheldrick, "SHELXT - integrated space-group and crystal-structure determination" *Acta Crystallographica Section A: Foundations of Crystallography* **71**, (2015): 3.

[22]	S. Grimme, "Exploration of Chemical Compound, Conformer, and Reaction Space with Meta-Dynamics Simulations Based on Tight-Binding Quantum Chemical Calculations" *Journal of Chemical Theory and Computation* **15**, (2019): 2847.

[23]	P. Pracht, F. Bohle, S. Grimme, "Automated exploration of the low-energy chemical space with fast quantum chemical methods" *Physical Chemistry Chemical Physics* **22**, (2020): 7169.

[24]	P. Pracht, S. Grimme, C. Bannwarth, F. Bohle, S. Ehlert, G. Feldmann, J. Gorges, M. Müller, T. Neudecker, C. Plett et al., "CREST-A program for the exploration of low-energy molecular chemical space" *The Journal of Chemical Physics* **160**, (2024).

[25]	S. Grimme, F. Bohle, A. Hansen, P. Pracht, S. Spicher, M. Stahn, "Efficient Quantum Chemical Calculation of Structure Ensembles and Free Energies for Nonrigid Molecules" *The Journal of Physical Chemistry A* **125**, (2021): 4039.

[26]	F. Neese, "The ORCA program system" *Wiley Interdisciplinary Reviews: Computational Molecular Science* **2**, (2012): 73.

[27]	F. Neese, "Software update: The ORCA program system—Version 5.0" *Wiley Interdisciplinary Reviews: Computational Molecular Science* **12**, (2022).

[28]	S. Grimme, A. Hansen, S. Ehlert, J.-M. Mewes, "r^2^SCAN-3c: A "Swiss army knife" composite electronic-structure method" *The Journal of Chemical Physics* **154**, (2021): 64103.

[29]	V. Barone, M. Cossi, "Quantum Calculation of Molecular Energies and Energy Gradients in Solution by a Conductor Solvent Model" *The Journal of Physical Chemistry A* **102**, (1998): 1995.

[30]	N. Mardirossian, M. Head-Gordon, "ωB97M-V: A combinatorially optimized, range-separated hybrid, meta-GGA density functional with VV10 nonlocal correlation" *The Journal of Chemical Physics* **144**, (2016): 214110.

[31]	F. Weigend, R. Ahlrichs, "Balanced basis sets of split valence, triple zeta valence and quadruple zeta valence quality for H to Rn: Design and assessment of accuracy" *Physical Chemistry Chemical Physics* **7**, (2005): 3297.

[32]	A. V. Marenich, C. J. Cramer, D. G. Truhlar, "Universal solvation model based on solute electron density and on a continuum model of the solvent defined by the bulk dielectric constant and atomic surface tensions" *The Journal of Physical Chemistry B* **113**, (2009): 6378.
